# Isotopic biographies reveal horse rearing and trading networks in medieval London

**DOI:** 10.1126/sciadv.adj5782

**Published:** 2024-03-22

**Authors:** Alexander J. E. Pryor, Carly Ameen, Robert Liddiard, Gary Baker, Katherine S. Kanne, J. Andy Milton, Christopher D. Standish, Bastian Hambach, Ludovic Orlando, Lorelei Chauvey, Stephanie Schiavinato, Laure Calvière-Tonasso, Gaetan Tressières, Stefanie Wagner, John Southon, Beth Shapiro, Alan Pipe, Oliver H. Creighton, Alan K. Outram

**Affiliations:** ^1^ Department of Archaeology and History, University of Exeter, Exeter, UK.; ^2^ School of History, University of East Anglia, Norwich, UK.; ^3^ Department of History, University of Southampton, Southampton, UK.; ^4^ School of Archaeology, University College Dublin, Dublin, Ireland.; ^5^ School of Ocean and Earth Sciences, University of Southampton, Southampton, UK.; ^6^ Centre for Anthropobiology and Genomics of Toulouse, Faculté de Médecine Purpan, Toulouse, France.; ^7^ Department of Earth System Science, University of California Irvine, Irvine, CA, USA.; ^8^ Department of Ecology and Evolutionary Biology, University of California Santa Cruz, Santa Cruz, CA, USA.; ^9^ Howard Hughes Medical Institute, University of California Santa Cruz, Santa Cruz, CA, USA.; ^10^ Museum of London Archaeology, London, UK.

## Abstract

This paper reports a high-resolution isotopic study of medieval horse mobility, revealing their origins and in-life mobility both regionally and internationally. The animals were found in an unusual horse cemetery site found within the City of Westminster, London, England. Enamel strontium, oxygen, and carbon isotope analysis of 15 individuals provides information about likely place of birth, diet, and mobility during the first approximately 5 years of life. Results show that at least seven horses originated outside of Britain in relatively cold climates, potentially in Scandinavia or the Western Alps. Ancient DNA sexing data indicate no consistent sex-specific mobility patterning, although three of the five females came from exceptionally highly radiogenic regions. Another female with low mobility is suggested to be a sedentary broodmare. Our results provide direct and unprecedented evidence for a variety of horse movement and trading practices in the Middle Ages and highlight the importance of international trade in securing high-quality horses for medieval London elites.

## Teaser

Isotopic tooth chemistry reveals international movements of medieval horses from elite horse cemetery site.

## INTRODUCTION

The significance of horses to life in medieval England cannot be understated. Procuring high-quality horses for labor, war, travel, and tournaments was of paramount importance. Historical sources indicate that the King and other high-ranking elites expended great effort and resources to breed, train, and otherwise acquire the best horses in England and abroad (*1*–*3*), but to date, insight into horse-trading and mobility has only been possible in a limited way through documentary sources, which are patchy and inconsistent (*1*). To address this lacuna, the project “Warhorse: The Archaeology of a Military Revolution?” (AH/S000380/1) conducted the first ever systematic and integrated archeologically based study of the physical remains, material culture, and landscapes of horse breeding and training in medieval England. Here, we present a case study from this project on the origins and early-life mobility of medieval horses found buried in the exceptional horse cemetery site of Elverton Street, located in the City of Westminster, London, England. Horse burials are rare in the medieval period and horse cemeteries are virtually unknown. While isolated articulated skeletons are occasionally found, horse bones were usually deposited disarticulated and commingled with other faunal material in middens, waste pits, and on sites associated with the processing of animal by-products. Even the carcasses of prestige animals from the royal stud network were routinely sold for their skins (*4*). The only comparable medieval site in the U.K. is Jennings Yard in Windsor (*5*), where the partial horse carcasses of at least eight individuals with evidence of skinning were found discarded in a gulley, whereas Elverton Street produced over 70 whole or partial horses. Like Windsor, Elverton Street is located close to a site of royal power, lying near the principal seat of government in medieval England centered on the palace-abbey complex of Westminster. This paper therefore considers the extent to which animals interred in this unusual context are also of special interest and highlights how isotopic biographies of individual horses can identify not only points of origin but also details of in-life movement in a way that could not be reconstructed from any other source.

### The Elverton Street site

**Fig. 1.**
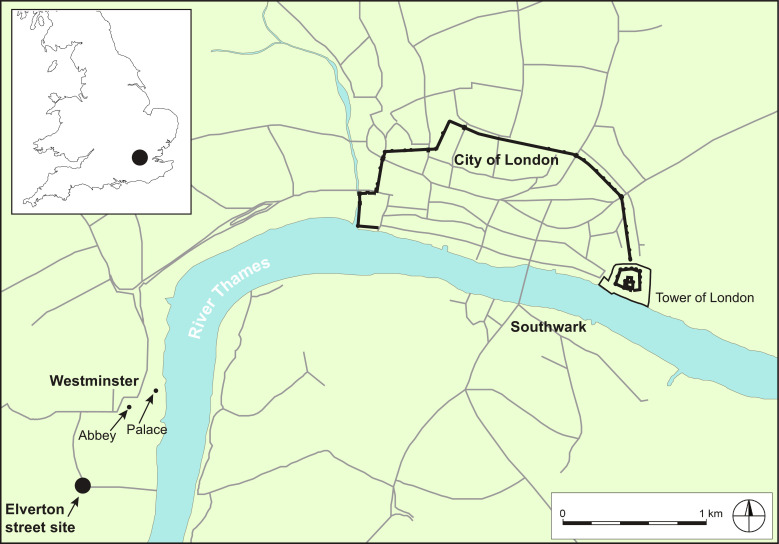
Location of the Elverton Street horse cemetery in late medieval London. Gray lines indicate major roads.

The Elverton Street site (longitude and latitude of −0.133 and 51.495, respectively) was unearthed during commercial excavations at two adjacent sites, 1 and 17 Elverton Street, by Museum of London Archaeology; site codes: ELV94 and EVT95) in the mid-1990s in advance of housing redevelopment (*6*). Within historic London, the site was positioned on the corner of Horseferry Road on the floodplain of the River Thames in a marshy area with very little archeology (*6*). Located ~2.5 km southwest of the walled city of London (Fig. 1), this was a sparsely populated area in the later medieval and Tudor periods, situated well beyond the fringes of the urban zone (text S1, section 1). The medieval royal-ecclesiastical complex of Westminster lies ~0.75 km to the northeast. In the late medieval period, the area occupied by Elverton Street was common pasture within the manor of Westminster, used for grazing and occasionally fairs, banquets, combats, and tournaments (*6*).

Excavations at Elverton Street revealed the site to be an unusual example of a horse burial ground. Across the entire site, 197 pits were found, of which 56 (28%) contained animal remains (*6*). The burial pits contained almost exclusively horse bones, representing articulated examples of both partial and nearly complete skeletons buried in small groups of between 1 and 8 individuals per pit (*6*). The excavation stratigraphy shows that the burial pits would have been clearly visible on the surface while the cemetery was in use, as there is little evidence for intercutting of the pits or any reopening or reuse of pits once closed (*7*). Twelve radiocarbon dates on individual horse bones are available for the Elverton cemetery, including 2 dates measured in the 1990s (*6*) and 10 further dates reported here (text S1, section 1.2). The only artifacts directly associated with the horse remains were two horseshoes, of Clark’s “Type 4,” attributable to the mid-14th to 16th centuries AD (*8*). Postmedieval activity at the site, as well as historical records of land use around adjacent Tuthill Fields, indicate that from 1605, the area of the site was the location of a sand quarry, which postdates all activity associated with the burial pits (*6*, *7*). A simple Bayesian model run in Oxcal (*9*, *10*) using the 12 radiocarbon dates and a terminus *ante quem* of 1605 returns a calibrated date range of AD 1425 to 1517 at 95.4% probability (text S1, section 1.2), although as a large number of burial pits remain undated, it is possible that the cemetery was used more widely throughout the late medieval and early Tudor period (i.e., between the 14th and 16th centuries).

## RESULTS

### Defining a “local” London ^87^Sr/^86^Sr range

The City of London lies on bedrock geology belonging to the Eocene epoch Thames Group formation, comprising marine-derived clay, silt, sand, and gravel (*11*). Close to the River Thames, including the Elverton Street site which is located less than 800 m from the present-day river channel, this bedrock is capped by Quaternary alluvial deposits. Strontium isotope ratios in dentine and cementum from 10 Elverton horses indicate a local bioavailable ^87^Sr/^86^Sr range at the burial site spanning 0.709064 to 0.710008 ($\bar{x}$ = 0.709564; σ = 0.000261; text S1, section 2.1). This matches previously reported bioavailable strontium ranges for London of ~0.709 to 0.710 based on plants (*12*), archeological humans dating from the Roman to postmedieval eras (*13*, *14*), and modeling studies (*15*, *16*).

Large areas of England, as well as parts of Wales and Scotland, also show biosphere ^87^Sr/^86^Sr ratios coincident with the local London range between 0.709 and 0.710 (*12*, *15*). Mature horses arriving in London from these areas will thus be indistinguishable from animals born and raised locally, rendering horse-trading networks within these areas essentially invisible in strontium isotope terms. This biosphere equifinality means that it is also impossible to identify locally London-born horses with any certainty. Conversely, the fact that the U.K. strontium isoscape varies mostly within a relatively tight range of 0.708 to ~0.711 within an ~200-km distance of London means that any enamel ^87^Sr/^86^Sr ratios falling outside this range are a good indicator for long-distance mobility of the horses concerned.

### Two M2-M3 pairs from Horse 7

**Fig. 2.**
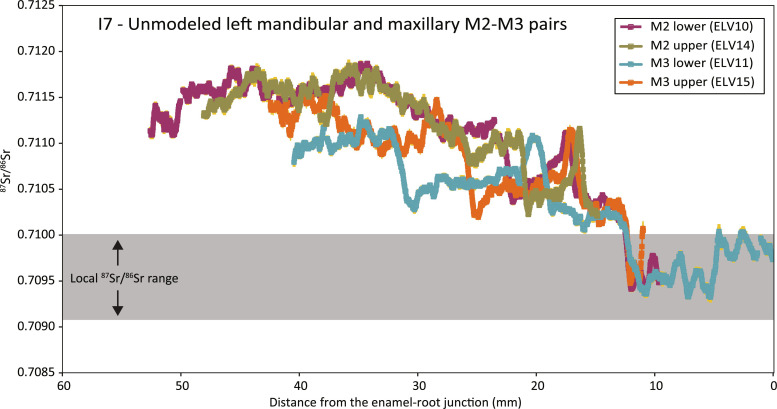
Strontium isotope profiles for mandibular and maxillary M2-M3 pairs from Individual 7. Data are shown without the enamel growth rate modeling proposed by Bendrey *et al.* (*17*) and aligned arbitrarily to the rapid change in ^87^Sr/^86^Sr ratios at ~12 mm from the enamel-root junction. The lines represent a moving 10-point mean average of the raw laser ablation data. Uncertainty is represented by the SE shown as yellow shading around the lines, although this is hidden almost everywhere by the thickness of the lines used to show the mean ^87^Sr/^86^Sr data.

**Fig. 3.**
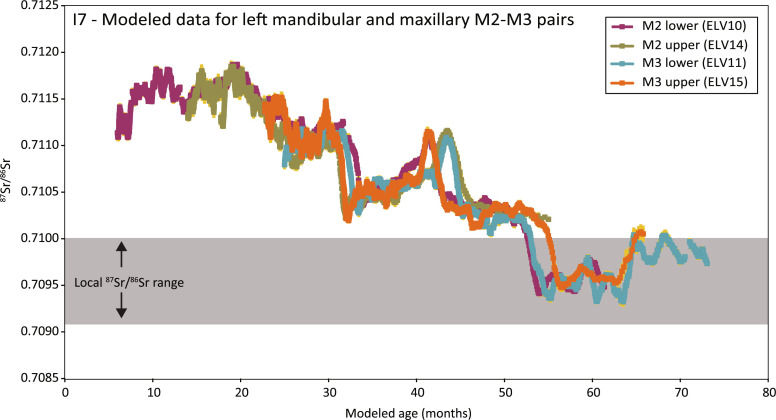
Strontium isotope profiles for mandibular and maxillary M2-M3 pairs from Individual 7 aligned using the exponential model of tooth growth by Bendrey *et al.* (*17*). Tooth wear is not reflected in this model and the predicted age in months is illustrative only. The model assumes that the mandibular M2 commenced growth at 6 months of age. Growth start times for all other teeth and tooth growth intervals for all teeth were then varied systematically to obtain the best possible fit. It is immediately obvious that applying Bendrey *et al*.’s (*17*) model assuming exponentially decreasing tooth growth rates results in a markedly improved alignment between the profiles. See the caption to Fig. 2 for an explanation of the error depiction.

The opposing M2-M3 pairs sampled from the left mandible and left maxilla of Horse 7 (four teeth in total) produced similar ^87^Sr/^86^Sr profiles recording the same mobility events, demonstrating temporal overlap as expected between the teeth. The speed of enamel mineralization in horses is known to be nonlinear (*17*). Comparing profiles between the four molars from Horse 7 shows that they cannot be directly aligned by wiggle matching due to profile compression in the basal ~10 to 15 mm of each profile (Fig. 2). Critically, though, the strontium isotope maxima and minima of each mobility event remain the same in all sampled teeth, despite the different degrees of time averaging at different stages of enamel mineralization. Applying a simple model of exponentially slowing tooth growth goes some way toward accounting for the nonlinearity effects [equation 4 from (*17*)], producing a reasonable alignment between the four strontium isotope profiles and demonstrating that the upper and lower M2s started and finished mineralizing before both M3s, as expected (Fig. 3) (*18*). In combination, and acknowledging the small discrepancies between them, the four ^87^Sr/^86^Sr profiles reflect (i) the different but overlapping formation times of each molar; (ii) the progressively decreasing speed of tooth formation, resulting in units of time being compressed into a shorter length of enamel near the enamel-root junction; (iii) differences in tooth wear and thus crown height available for sampling in each molar; and (iv) averaging effects during enamel mineralization and specific enamel geometry at the sampling location in each molar.

### Elverton enamel ^87^Sr/^86^Sr profiles

**Fig. 4.**
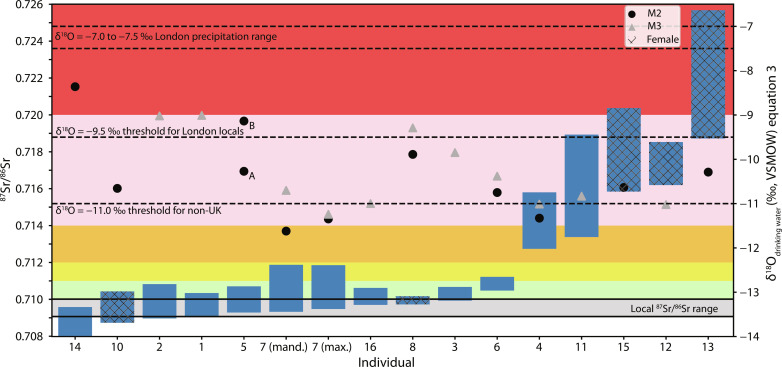
Strontium and oxygen isotope data for 15 Elverton horses. Blue bars indicate the strontium isotope range measured in each individual. Background shading correlates to the strontium isotope zoning used in Fig. 6A. Black dots and gray triangles indicate estimated δ^18^O_drinking water_ values calculated from the tooth enamel data using equation 3 (*29*). For similar plots showing ^87^Sr/^86^Sr with respect to δ^18^O_drinking water_ calculated using equation 4 (*30*) and δ^13^C, see text S1, section 2.5.

**Fig. 5.**
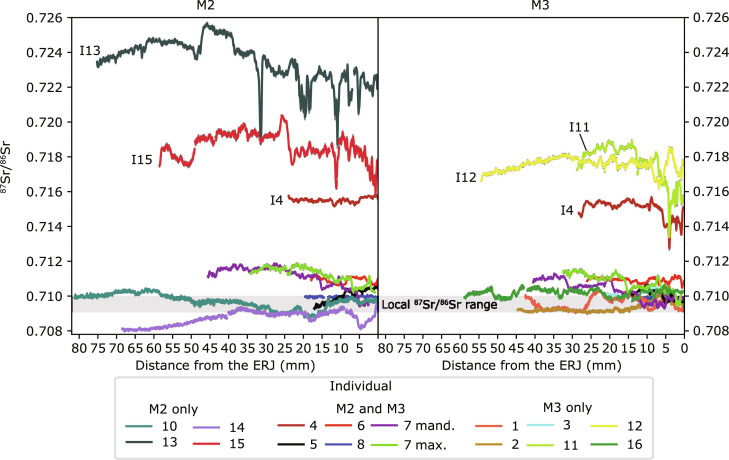
Strontium isotope profiles of the Elverton horses. The lines represent a 10-point mean moving average of the raw laser ablation data. Uncertainty is represented by the SE shown as dark gray shading around the lines, although this is mostly hidden by the thickness of the lines used to show the mean ^87^Sr/^86^Sr data. The local biosphere ^87^Sr/^86^Sr range for London is indicated by the bar-shaded gray.

Enamel ^87^Sr/^86^Sr profiles from 22 teeth representing 15 horses span an exceptionally wide range from 0.70798 to 0.72567 (Figs. 4 and 5 and data S2). Individual 8 is the only sampled individual to have an ^87^Sr/^86^Sr profile consistent with remaining within London for the whole period captured by the sampled enamel. All other horses spent at least some time during the enamel formation interval outside of London, including six individuals that show no overlap with the “local” bioavailable ^87^Sr/^86^Sr London range in any part of their enamel profiles. Four of these latter individuals (Horses 11, 12, 13, and 15), including three of the five identified females, produced exceptionally radiogenic ^87^Sr/^86^Sr profiles almost entirely above 0.716. In Horse 13, the ^87^Sr/^86^Sr profile rises as high as 0.72567 which, to our knowledge, is the most radiogenic signature yet measured in any archeological human or fauna found in the U.K. from any period. One further individual (Horse 4) also produced very high ^87^Sr/^86^Sr ratios falling between 0.714 and 0.716 for a substantial part of both its M2-M3 profiles. Meanwhile, 4 of the 15 sampled horses (Individuals 1, 2, 5, and 10) show substantial overlap in their ^87^Sr/^86^Sr profiles with the local London range but also some mobility across more radiogenic geologies with values up to 0.7110. Horse 14 is the only individual to spend long periods on geologies less radiogenic than the local London range, with ^87^Sr/^86^Sr ratios of 0.7080 being the lowest measured in this study. Strontium isotope ratios in this range are consistent with the chalkland geologies of south and east England, indicating a potential origin for this horse. Strontium isotope profiles for all other horses either coincide with the local London range or show more radiogenic values, definitively ruling out the chalkland geologies as potential places of origin for all individuals apart from Horse 14.

Geologies that could impart such highly radiogenic signatures above 0.714 are rare within the mainland U.K., where the total ^87^Sr/^86^Sr range falls mostly between 0.707 and 0.715 (*15*, *19*). Biosphere ratios between 0.7115 and 0.7130 can be found only in southwest England, discrete parts of the Midlands, and parts of Wales and Scotland (*12*, *20*). Biosphere ratios above 0.7132 are observed even more rarely in restricted areas of the landscape such as the Malvern Hills (0.714), other areas close to the English-Welsh border (~0.716), and in the Lake District (*15*, *21*, *22*), with some isolated patches of highly radiogenic geologies producing ratios of up to 0.7165 in Wales (*23*), between 0.7165 and 0.7182 in Scotland (*19*, *22*) and, exceptionally, up to 0.7287 on parts of Dartmoor centered on rubidium-rich granites (*24*). Further localized areas of highly radiogenic deposits may remain unrecognized in Britain (*24*); in addition, the so-called forest effect can potentially raise ^87^Sr/^86^Sr ratios by up to 0.003 to 0.004 in areas of ancient forest, which will have affected larger areas in the medieval period compared with today (*25*). It is unlikely, however, that these factors would have extended the total British biosphere range in any meaningful way. This point is demonstrated by two large-scale studies of enamel ^87^Sr/^86^Sr from humans: The first indicates a U.K.-wide range of 0.7078 to 0.7165 based on samples dating from Neolithic to 19th century AD (*26*); the second indicates a wider range of 0.7064 to 0.7205 based on samples spanning the Late Roman to early medieval periods, but the latter contained just seven entries >0.714 for the U.K. and all were considered to be first-generation immigrants from Scandinavia [see (*27*) and source publications cited therein]. Meanwhile, a recent large diachronic analysis of U.K. archeological faunal remains dating from the Neolithic to late medieval periods produced a similar total range between 0.7082 and 0.7172 (*28*). The reduced range of ^87^Sr/^86^Sr ratios observed in human and faunal enamel compared to U.K. biosphere values reflects the geographically limited extent of geologies at the margins of the total isoscape range and demonstrate a de facto range for tissues of mobile British mammalian species consuming food and water from disparate sources during the period of enamel formation. In this context, the Elverton horse assemblage stands out for both the exceptional range in Sr isotope ratios within the sample set and for the highly radiogenic individuals at the upper end of the range.

### Elverton enamel δ^18^O data

The enamel carbonate data represent a temporally averaged signal approximating an annual mean average of δ^18^O and δ^13^C, respectively (see Materials and Methods). The δ^18^O_enamel_ results were converted from Vienna Pee Dee Belemnite (VPDB) to the Standard Mean Ocean Water (SMOW) isotopic scale, and then calibrated from carbonate to a phosphate enamel equivalent (text S1, section 2.5). Mean δ^18^O_drinking water_ was estimated using two horse-specific enamel—drinking water relationships calculated respectively by Delgado Huertas *et al*. (*29*), referred to here as equation 3, and the revised equation proposed recently by Pederzani *et al*. (*30*), referred to here as equation 4. Across the data range of interest for this analysis, equation 4 produces systematically lower δ^18^O_drinking water_ estimates than equation 3 and directly affects how the Elverton data are interpreted. Our analysis therefore uses δ^18^O_drinking water_ estimates from both these equations for comparative purposes (further discussion of the differences between these equations is given in text S1, section 2.5).

Elverton horse δ^18^O_drinking water_ ranges between −8.4 and −11.6 per mil (‰) when using equation 3 (*29*), and −8.8 to −12.6‰ when using equation 4 (*30*). These ranges are substantially lower than recent mean annual δ^18^O_precipitation_ in and near London which averages −7.0 to −7.5‰ (*15*, *31*) and also extend lower than the seasonal precipitation isotope range for this region which varies between an average of −4.8‰ (σ = 1.9) in summer and −8.3‰ (σ = 2.1) in winter for 41 years of available data (*32*); for comparison, the OIPC average is −7.1‰ with a modeled seasonal range of −3.0 to −9.9‰ (*31*, *33*, *34*). Many horse δ^18^O_drinking water_ estimates are also lower than mean annual δ^18^O_precipitation_ for the whole of Britain, which varies between approximately −4.5 and −7‰ across south and west Britain and −7 and −9‰ in east and north Britain, respectively (*15*, *26*). Rare instances of spring waters with δ^18^O_groundwater_ values between −9 and −10‰ have been reported (*26*), although it is unlikely that any Elverton horse could have consumed water exclusively from one of these sources. Furthermore, well-mixed British groundwaters show a similar lower limit to that observed in mean annual δ^18^O_precipitation_ of about −9‰ (*26*, *35*). Regardless of which equation is used to calculate horse δ^18^O_drinking water_, our results therefore indicate that Elverton horses consumed waters across a notably lower range of oxygen isotope values than that defined for both modern London δ^18^O_precipiation_ and contemporary U.K. groundwaters more generally.

### Elverton enamel δ^13^C data

Elverton horses had mean δ^13^C of −13.3‰ (2σ = 0.9‰), indicating that all individuals consumed a pure C3 diet over the approximately 1 year of enamel growth captured by the samples. Outlier analysis using a threshold of median ±1.5* interquartile range (IQR) for identifying outliers indicated that no individuals are statistically outlying in δ^13^C, although Individual 16 is notably enriched and Individuals 12 and 14 are marginally depleted in ^13^C relative to all other horses (text S1, section 2.5).

## DISCUSSION

### Interpreting the δ^18^O data

The low horse δ^18^O_enamel_ and associated δ^18^O_drinking water_ values are unlikely to be a consequence of cooler climates relative to today. The Elverton horse burials coincide temporally with the early phase of glacier expansion associated with the Little Ice Age beginning approximately 1300 AD but show a maximum potential overlap of just 50 years with the start of the Little Ice Age climate (approximately 1550 to 1900), a period of generally cooler and drier conditions across the north Atlantic region although with some localized variability in the seasonality of these impacts (*36*, *37*). Consequently, any Elverton horses born between 1550 and 1600 AD could potentially show lower δ^18^O_enamel_ and δ^18^O_drinking water_ values relative to modern comparators due to cooler mean annual temperatures. A relative increase in winter-time precipitation as a share of total annual rainfall could also cause the same isotopic effect; however, these impacts would not apply to any horses that predate 1550, as do all of the currently radiocarbon-dated horse bones from Elverton. In addition, diachronic studies suggest that quantitative impacts of climatic fluctuations over the past 4000 years on human δ^18^O_enamel_ in the U.K. are relatively small, at most perhaps 0.5‰ (*26*) and the same pattern likely holds true for other large mammalian species such as horses too. Meanwhile, physical processes such as transpiration from plant leaves and evaporation can only raise the δ^18^O of metabolic water sources rather than lowering it, and there are no known cultural practices that can decrease δ^18^O_drinking water_ or δ^18^O_enamel_ relative to their climate-linked values (*38*).

Horses typically consume 20 to 80 liters of water daily depending on activity rates, pregnancy, or lactation (*39*). Even in an extreme example where a horse drank exclusively from a single restricted water source managed by humans (e.g., a well- or rainwater-fed trough), the requirement for horses to consume large volumes of water daily ensures a well temporally averaged signal with no known mechanism for lowering δ^18^O_enamel_ values relative to precipitation/groundwater baselines at the point of origin. Our estimates of δ^18^O_drinking water_ are therefore likely to be reasonable indicators of the precipitation/groundwater context during the mineralization interval due to (i) the drinking habits of the horses themselves, (ii) attenuation caused by natural mixing in the (large volume) groundwater/drinking water pools available to the horses to consume, and (iii) by the temporal averaging introduced by our enamel sampling methodology (see Materials and Methods).

Given these points, it is appropriate to consider migration when interpreting the δ^18^O_enamel_ data. δ^18^O_enamel_ data has routinely been used in humans to identify potential migrants to Britain throughout the prehistoric and historic periods [for example, see, (*40*–*43*)], including analyses of immigration to London in the Roman period (*44*) and medieval period (*13*), respectively. These studies have typically used relatively large reference datasets to identify expected local population-level δ^18^O_enamel_ ranges and used outlier analysis to identify potential migrants; calibration from δ^18^O_enamel_ to estimated δ^18^O_drinking water_ followed by comparison to contemporary isoscape base maps is either not undertaken or is used as supporting analysis to further interpret the data. This approach is unfortunately not viable in the present study because our dataset is relatively small (15 individuals) and we lack comparative data from other British horses, medieval or otherwise, from which to calculate a horse-specific local population baseline. It is therefore necessary to make direct comparisons between our calibrated horse δ^18^O_drinking water_ data and modern isoscape base maps. This method introduces extra unavoidable uncertainties regarding the relevance of modern isoscapes to archeological contexts, given the potential for changing climates, atmospheric circulation patterns, shifting moisture sources, shifting seasonality of precipitation, and other such factors which could all affect the local meteoric baseline, as well as the uncertainties associated with calibration from δ^18^O_enamel_ to δ^18^O_drinking water_ itself. We note, however, that previous studies researching migration to London and Britain more generally (*13*, *40*–*44*) all consistently identify a local London δ^18^O_drinking water_ value of around −7‰ and a British-wide lower limit of around −9‰, collectively spanning from the prehistoric to the medieval era. This strongly suggests that these reference values are also appropriate when considering the Elverton horses.

Intrapopulation behavioral and physiological differences of individual horses and interlaboratory differences in analytical procedures require that a ±2‰ interpretative buffer is used when separating likely local and nonlocal individuals (*38*, *45*). A ±2‰ buffer also mitigates against the uncertainties introduced when estimating δ^18^O_drinking water_ values from δ^18^O_enamel_ data (*46*). Assuming the modern water reference dataset is appropriate for the medieval context being studied herein, relative to local mean δ^18^O_precipitation_ of −7.0 to −7.5‰, δ^18^O_drinking water_ values of >−9.5‰ in the Elverton dataset should be regarded as consistent with local London origins; for origins throughout the whole U.K., the equivalent lower baseline is >−11‰ (i.e., 2‰ lower than −9‰, the bottom of the U.K. precipitation/groundwater range). Horses with estimated δ^18^O_drinking water_ falling below these ranges may therefore be considered potentially of non-London or non-British origin respectively.

### Identifying local London and domestic British horses

Combining the oxygen and strontium isotope results indicates that a maximum of six individuals, and potentially just three or fewer individuals, were born and raised in or near the city of London or the nearby chalk uplands (Fig. 4). Horses 1, 2, and 14 each show clear overlap with local ranges in both isotope systems. Notably, Horse 14 produced the highest δ^18^O and one of the lowest δ^13^C measurements in the dataset, suggestive of relatively warm and wet climates compared to those experienced by other horses found at Elverton. Horse 14 is also the only individual to show ^87^Sr/^86^Sr values consistent with British chalklands, found widely across southeast Britain. The mobility profile from its unworn M2 is suggestive of repeated movements throughout tooth growth (9 months to 3 years) between the chalklands and surrounding territories, potentially including visits to London (data S2). Horse 8 may also be a local, with a strontium profile falling at the upper limits of the local range and estimated δ^18^O_drinking water_ around the lower limits of the expected range. Meanwhile, two further horses, Individuals 5 and 10, have strontium isotope profiles that overlap substantially with the local London range and could potentially also be locally born, although this would require these horses to be consuming waters substantially more depleted in ^18^O than expected for a London context and therefore seems unlikely.

Rather, including Horses 5 and 10, there are collectively four horses (Individuals 3, 5, 6, and 10) that have ^87^Sr/^86^Sr profiles mostly above 0.7092 and entirely below 0.7113 and estimated δ^18^O_drinking water_ in the range −9 to −11‰, i.e., lower than the current U.K. δ^18^O_precipitation_/δ^18^O_groundwater_ range but not sufficiently so as to confidently indicate an international origin. Lithologies conferring ^87^Sr/^86^Sr ratios in the appropriate range can be found widely both within the U.K. and abroad and the data overall are therefore not geographically specific for these individuals. These four individuals are therefore plausibly of non-London British origin, but origins internationally cannot be ruled out and become more likely if δ^18^O_drinking water_ is calculated using equation 4 instead of the more conservative equation 3.

### Identifying non-British horses

Five horses (Individuals 4, 11, 12, 13, and 15), including three of the five females, have exceptionally radiogenic ^87^Sr/^86^Sr profiles almost entirely above 0.713 and up to 0.72567 and predicted δ^18^O_drinking water_ values (calculated using equation 3) either just above or just below the −11‰ threshold used to separate U.K. and non-U.K. natives. Individual 13 produced the most radiogenic strontium values yet detected in the British archeological faunal record, ranging up to 0.72567 and only rarely falling below 0.7220. British biosphere strontium isotope ratios this high have only previously been found in parts of northeast Dartmoor, centered on granite outcrops enriched in rubidium with ratios between 0.7143 and 0.7287. Similarly, high ratios might also occur on other granite outcroppings of the Cornubian batholith including southwest Dartmoor, parts of Bodmin moor, and other locations in central and west Cornwall (*24*). These locations could theoretically account for the very high ^87^Sr/^86^Sr profiles in the Elverton horses including Horse 13; however, the δ^18^O data show that Horse 13 consumed drinking waters much too depleted in ^18^O to have come from southwest Britain (equation 3 = −10.3‰; equation 4 = −11.0‰), where instead some of the most isotopically enriched (in ^18^O) precipitation observed across the whole U.K. is recorded (*15*). In combination, the isotopic data therefore definitively rule out any possibility of Horse 13 originating anywhere in Britain.

**Fig. 6.**
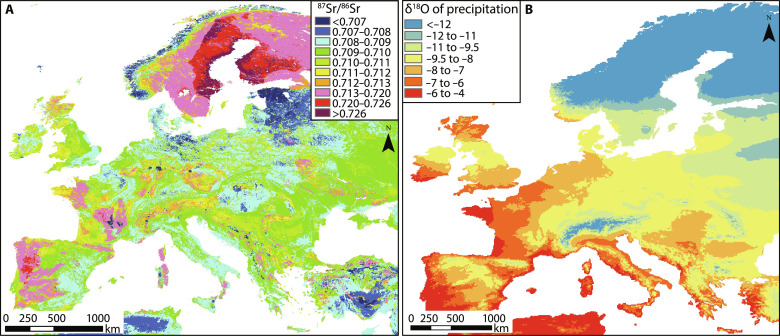
Biosphere strontium and δ^18^O_precipitation_ maps of Europe. (**A**) Bioavailable ^87^Sr/^86^Sr isoscape using data from (*16*). (**B**) European mean annual δ^18^O_precipitation_ based on the OIPC v3.2 database (*31*, *33*, *34*); grid resolution is 5 arc min. Maps prepared using ArcMap 10.8.1.

Within Europe, biosphere ^87^Sr/^86^Sr ratios above 0.720 are rare (Fig. 6) and mostly associated with Precambrian or Paleozoic lithologies composed of schists, gneisses, and other felsic igneous rocks such as granites (*16*, *19*, *47*, *48*). Cross-referencing these locations against areas with appropriate δ^18^O_precipitation_ values suggests that south Sweden, south Finland, the Danish island of Bornholm, and the Central/Southern Alps are the most viable source locations for Horse 13 in Europe based on current base-mapping data. Of these, Bornholm Island seems unlikely, as Horse 13 shows evidence of regular mobility during tooth growth with Sr variation between 0.719 and 0.726, whereas mobility on Bornholm Island would have been difficult without encountering substantially less radiogenic lithologies (*49*). South Sweden, south Finland, and the Alps are all plausible source locations, from both chemical and cultural perspectives [see (*50*, *51*) and references therein]. In particular, crystalline rocks in the Western and Central Alps are known for producing highly radiogenic signatures (*48*, *52*), and the area was active during the late medieval period in terms of horse breeding and trading (*53*–*55*).

Female Individuals 12 and 15 also produced exceptionally radiogenic Sr isotope profiles almost entirely above 0.7165 and up to 0.7203 (data S2), ratios known in the U.K. only from specific parts of the Cairngorm Mountains in Scotland or southwest England. Like horse 13, δ^18^O_drinking water_ values for these individuals are well outside the range for the southwest convincingly ruling it out as a place of origin. Meanwhile, Scottish geologies producing such highly radiogenic ^87^Sr/^86^Sr ratios are highly localized and mostly located close to geologies with much lower bioavailable signatures (*19*), which are not evidenced in the tooth Sr profiles of either horse despite signs of mobility during tooth formation. Origins in Scotland would additionally require both horses to be at the margins of the U.K. population range for δ^18^O. We therefore consider Scottish origins to be extremely unlikely for Individuals 12 and 15. The same arguments and interpretation apply to two male horses (Individuals 4 and 11); although Scottish origins are theoretically possible based on the isotopic data, this is very unlikely given the consistently highly radiogenic ^87^Sr/^86^Sr profiles, low δ^18^O_drinking water_ estimates, and evidence for mobility during tooth formation in both individuals.

It is much more likely that Individuals 4, 11, 12, and 15 all had international origins in areas where strongly radiogenic bioavailable ^87^Sr/^86^Sr ratios are found more widely and in climates substantially colder than the present-day U.K.. Possible source locations for these individuals are south Sweden, Lithuania, the Pyrenees mountains, or more radiogenic parts of the Western and Central Alps, the southwest Bohemian Massif, or the Carpathian Mountains (*16*, *19*, *47*, *48*, *50*), but this is not an exhaustive list. It is additionally conceivable that Horses 11, 12, and 15 (found in three different burial pits) all came from the same source region, and even from the same farm or breeder or stud, given the isotopic similarity of these individuals.

Two other individuals, Horses 7 and 16, produced strontium profiles well within the U.K. range, but both consumed drinking waters very unlikely to be of British origin (~−11 to −11.6‰ using equation 3; −11.5 to −12.6‰ using equation 4), suggesting international origins in a cooler climate. The ^87^Sr/^86^Sr profile for Horse 7 records a series of movements throughout the tooth growth interval across a range of different lithologies. The combined Sr and δ^18^O_enamel_ data are suggestive of cool-climate origins somewhere in continental Europe, for example, southwest Norway, the Alps, the Bohemian Massif uplands, or the Carpathian Mountains. Then, between approximately 2.5 and 4+ years of age, the ^87^Sr/^86^Sr profiles record at least four mobility events between different lithologies, ending in a location isotopically indistinguishable from London with ^87^Sr/^86^Sr below 0.710, from ~4.5 years old. The distances covered during these mobility events are unknown and may relate either to mobility close to the birth location, mobility within continental Europe before Horse 7 was transported to London, or conceivably the isotopic data may have recorded the entire journey from birth location, through various trading networks to the moment when Horse 7 arrived in London, followed by a period of initial mobility within or around the city itself. Whichever, the ^87^Sr/^86^Sr profiles attest that Horse 7 visited a number of different lithologies during tooth growth, potentially including repeated visits to the same location months apart, but certainly not including any return to its place of birth.

In summary, the combined isotopic data indicate that five of the 15 sampled horses are of diverse, non-British origin (Individuals 4, 11, 12, 13, and 15), and at least two more are also very likely to be of non-British origin (Individuals 7 and 16) (Table 1). As many as 12 of the 15 sampled horses were plausibly nonlocal to London, coming from disparate sources and with most individuals arriving in London only after the sampled teeth were fully grown. This equates to older than ~3 years of age for M2s and ~4.5 years for M3s. Evidence for mobility from mares as well as stallions or castrates suggests that mares maintained value as imports, either for breeding or other service capacities. This is supported by evidence of bit wear on some individuals (e.g., Horse 10), potentially suggesting dual roles for both breeding and use. The isotopic results and unique mobility histories of each individual horse are further summarized in text S1, section 3.

**Table 1.** Summary of Elverton horse mobility and likely origins.

Horse no.	Sex	Origin	Approximate age when mobility begins (if recorded)	Inferred mobility
1	M	Britain	Unknown	Mobile (within SE England?) after ~2.5 years
2	M	Britain	3.5 years	Sedentary, then mobile (within SE England?) after ~3.5 years
3	M	Britain	Unknown	-
4	M	International	3.5 years	Sedentary, then mobile after 3.5 years
5	M	Britain	Unknown	Mobile after ~2 years.
6	M	Britain	Unknown	Mobile
7	M	Probably international	1.5–2 years?	Born abroad, journey to London recorded in enamel profile?
8	F	Britain	Sedentary	Potentially resident in London region throughout recorded period
10	F	Britain	Unknown	Mobile
11	M	International	Unknown	Regular mobility from ~3 years
12	F	International	3 years	Sedentary, then mobile after ~3 years
13	F	International	1.5 years	Sedentary, then mobile after 1.5 years
14	M	Britain	<12 months	Origin in English chalk geologies; mobile in SE England between 9 months to 3 years
15	F	International	<12 months	Mobile from an early age
16	M	Probably international	Unknown	Mobile

### Sedentism reveals aspects of medieval horse breeding and training practices

Horses 2 (M3), 4, and 8 (M2-M3 pairs) all show periods of exceptional stability in their ^87^Sr/^86^Sr profiles related to periods of immobility (e.g., restriction to specific pastures) during certain phases of the horses’ lives (text S1, section 2.4). Horse 8 is a more than 20-year-old female whose M2-M3 pair showed a high degree of wear. The strontium data from Horse 8 describes two relatively short snapshots in time ~6 to 12 months long and approximately 18 to 24 months apart, with a stable ^87^Sr/^86^Sr profile just within the upper end of the local London range. Given the similarity between the M2 and M3 data, it is reasonable to suggest that Horse 8 remained immobile during the first 4 to 5 years of life, as might have occurred for a stabled horse or pastured breeding mare living on a stud with minimal mobility or dietary variation needed beyond rich pasture. Medieval horse management of this type is suggested for mares in the hippiatric treatise by Jordanus Rufus in the 13th century (*56*).

Data for Horses 2 and 4 similarly suggest that both remained immobile for the first approximately 4 years and between 2.5 and 3.5 years of life respectively before a recorded period of mobility (data S2). Horses 12, 13, and, to a degree, 15 (all with highly radiogenic profiles) also show periods of relative stability in the earlier parts of their ^87^Sr/^86^Sr profiles, which preceded the start of more mobile lifestyles beginning at various points between approximately 18 months and 3.5 years of age (*18*) (text S1, section 3). On the basis of this patterning, our analyses suggest a mobility signature related to the breeding, rearing, and trading of horses, following guidance in hippiatric treatises, and as recorded in royal documents (*56*–*58*). Early 14th-century accounts from royal keepers in England suggest that horses resided at the “king’s stud farms through until their second or third year, at which point they would either be broken and trained or sent elsewhere to be sold” (*57*). The Elverton horses seemingly match this pattern, with birth at a stud in one location, turnout after weaning on the same or another park before formal training began between 1.5 and 3 years, at possibly yet another locale, at which point horses would be ready for either sale or service between 3 and 6 years old. Given the diverse and international origins of the Elverton horses, it is interesting that the human-controlled mobility trajectories of horses born and bred in different places appear to have been similar.

### The horses of Elverton and London: Isotope biographies in historical context

The varied origins and movements of the Elverton Street horses reflect the diversity of London’s late medieval and Tudor horse population. Historical sources provide further context for the isotopic biographies of these specimens within the contemporary horse trade. Levels of horse ownership among medieval Londoners were likely low. While the capital was a burgeoning city, it was not so large that it could not be traversed on foot, making riding largely unnecessary (*8*). There was a far greater need for mounts ridden by those undertaking journeys outside the city, including for trade and pilgrimage. For these purposes, horses could be hired from hackneymen or from the inns that served the arterial routes connecting London to its hinterland (*8*). Additional to these were the pack and cart animals that transported commodities and foodstuffs into and across the city. A further notable group of horses was associated with the royal household and the secular and ecclesiastical elite, all of whom maintained urban palaces or houses provided with their own stables accommodating a range of horses, from elite mounts to riding and baggage animals. While the total number of horses in the late medieval city is unknown, demand for their services was clearly heavy as by the middle of the 14th century, it was noted by contemporaries that acquiring horses near London was prohibitively expensive (*59*). London was therefore not so much a place where horses were (necessarily) born, but one where they worked and died. As only one individual from Elverton (Horse 8) could potentially have been resident in London throughout the tooth growth interval, it is within this context that we can seek to understand these horses’ biographies.

The historical evidence for breeding and trading suggests that individual animals were drawn from a wide geographical area; by the early 16th century, there was a nationwide “flow” of animals from breeding areas all over Britain, especially from the north and west (*2*). This pattern of movement comprised a series of distinct stages, with foals and juvenile animals tending to pass through a succession of different places as they were reared and trained before entering their working lives. Prime landscapes for rearing young horses existed to the north of London in the Chiltern Hills and to the south on the North Downs (*2*). From such places, stock could be brought directly to the capital for work or more probably for sale, at locations such as the celebrated Smithfield market with its weekly horse fair (*60*). Isotopic evidence for the operation of this system is clear: A transition from an origin landscape to a high-mobility service lifestyle typically occurs around 3 to 4 years old, with at least 7 of the 15 investigated horses arriving in London after tooth mineralization completed at ~5 years of age.

In addition to native stock, documentary sources attest that certain horses were sometimes imported from abroad, especially in elite contexts. The most celebrated centers of medieval horse breeding from which mounts were exported to England were southern Italy, Lombardy, via Alpine trade networks, and Spain (*1*). While we do have a broad outline understanding of which areas of Europe horses were sourced from, specific documentary references to the procurement of these animals are sporadic and often anecdotal. The principal documentary source for understanding royal horse breeding and training networks is the “Equitium Regis” (king’s stud) series, held by The National Archives (TNA), although this information is at its most detailed in the late 13th and 14th centuries. These stud accounts often indicate horses obtained by gift or purchase, and while the balance of these come from England, foreign imports can sometimes be identified, including through their names.

In the 16th century, the efforts made by Henry VIII to introduce foreign bloodlines, including from the Low Countries, to improve native stock are especially well-known (*2*). The royal stable in the 16th century was particularly cosmopolitan: In 1547, the 153 horses in the charge of Sir Anthony Brown, the Master of the King’s Horses, comprised 49 coursers, 13 stallions, 24 hobbies or geldings, 5 barbaries, and 6 jennets, the two latter types clearly of North African and Spanish origin, respectively. The remainder comprised 56 horses and mules that acted as baggage and riding animals (*61*). While the latter were probably drawn from similar areas to other non-elite horses, the higher-status mounts were either supplied from royal studs, gifted, or otherwise acquired from external sources. By the mid-Tudor period, the royal studs at Warwick, Malmesbury, Tutbury, Eskermayne, and an additional unnamed site in Wales contained a high proportion of breeding mares from Flanders, while the gifting of prestige animals, especially those from Iberia, the Mediterranean, and the Middle East, was a familiar part of royal exchange networks [(*61*) p. 39497)].

The combination of historical sources and isotopic analysis allows us to make some important points about the origins of the non-British horses from the Elverton Street cemetery. First, the nature of the isotopic methods means that it is far easier to rule out particular international breeding grounds than to designate definite places of origin for particular specimens. It is notable that, on the basis of strontium and oxygen values, none of the non-British horses (especially Individuals 4, 11, 12, 13, and 15) seem to have originated in the prime European horse breeding centers of Spain or southern Italy. On similar grounds, the Low Countries also appear an unlikely origin point, although it is important to bear in mind that not all horses traded through this region when the market was booming in the 15th and 16th centuries will have originated there, instead passing through the trading network from much further away. Given that several of the potential origin points proposed above for the non-British individuals were not renowned horse breeding landscapes or zones that traded regularly with London, one location that stands out as particularly credible in historical terms for the origins of some horses is the fringe of the Central Alpine region, where Swiss merchants were particularly active in the international horse trade (*53*, *54*) and where breeding grounds are well attested (*55*).

Last, moving to the potential significance of the geographical context of Elverton Street in historic London, the fact that the site lies immediately adjacent to Horseferry Road, and in the vicinity of the royal-ecclesiastical complex of Westminster, requires consideration (text S1 and fig. S1). The ferry across the River Thames is first mentioned directly in 1513 but must have existed much earlier, not least because it connected the Archbishop of Canterbury’s complex at Lambeth on the east bank to the royal palace and abbey at Westminster on the west bank (*62*). The cemetery setting, on the edge of an open area of common pasture immediately adjacent to a busy thoroughfare between Lambeth and Westminster, could suggest that it accommodated animals that expired on or close to this routeway, including pack or cart horses provisioning the Westminster complex. Perhaps more likely, but not incompatible with this suggestion, is a more direct connection with the royal complex of Westminster itself. Detailed records of the royal keepers of horses from the 14th century frequently note the name, date, and place of death of individual animals, and it is instructive that Westminster Palace features on a regular basis. For example, the palfrey Bausan de Cornwall died there on 16 October 1343 (*63*), while the destrier Grissel-Pomele de “Came” died together with an unnamed hackney on 14 January 1347 (*64*). While such records do not exist for the time when the Elverton cemetery was most intensively used, it is not without interest that these dates overlap with the earliest possible phases of activity (*6*) and so leave open the possibility that some of the burials may be horses from the royal household.

Westminster Palace was also a noted and busy royal tournament venue, where jousts were regularly held, while the pasture that adjoined the Elverton Street site was the venue for other tournaments and combats, although any link between the horses found at Elverton Street and these facilities can only be a matter of conjecture. Westminster was a venue for lavish courtly jousting tournaments that are well documented from the mid-15th century through to the early part of Henry VIII’s reign, to be superseded by an extravagant new purpose-built tiltyard immediately to the north at Whitehall (on the present-day site of Horse Guards) in the early 1530s (*65*–*67*). While any connection between the Elverton Street site, which was active in this very period, and a tournament venue might seem speculative, the case is potentially strengthened by the stature of the horses, many of which were above average in height for medieval English horses (*4*, *6*). Withers heights, calculated from the greatest length of long bones (*68*, *69*), averaged 1392.2 cm [13.2 hands high (hh)], significantly higher than the average from late medieval horses (13 hh) (text S1, section 1.3). The assemblage also included three of the tallest recorded horses known from late medieval England, ranging between 1.47 and 1.60 m (14.2 to 15.3 hh). Documentary evidence supports the idea that horses selected for the joust were the tallest and strongest of the medieval destriers (*70*), and there is a notable absence of horses below 12 hh at Elverton, in contrast with other late medieval assemblages (text S1, section 1.3). The Elverton horses were also some of the most robust late medieval horses documented, based on the ratio of the greatest length to the smallest width of the diaphysis of the metapodia. A number of fused lower thoracic and lumbar vertebrae identify at least some of the Elverton horses as mounts, with additional pathologies such as spavin and splints common to working and aged animals. Unusual bit wear recorded on the second lower premolar of Horse 5 is consistent with heavy use of a curb bit, typically used on highly trained elite horses from the 14th century onward (*71*).

The Elverton Street site and its associated horse assemblage are characterized by a number of unusual features: (i) the exceptionally unusual form of cemetery deposition, (ii) its elite location close to the royal complex of Westminster, (iii) the high proportion of exogenous animals imported from abroad, and (iv) the presence of some unusually large and robust horses within the assemblage. While none of these features are necessarily unique to high-status horses, in combination, they suggest strongly that the Elverton horses are elite animals owned by elite households, including possibly the royal household itself. The isotopic data presented here provide direct evidence linking specific horses to the long-distance horse-trading networks that supplied the medieval London elite and provide a baseline understanding against which future studies of non-elite horse-trading in London and Britain more broadly can be assessed.

## MATERIALS AND METHODS

### Sampled teeth

Twenty-two M2 and M3 molars from 15 individuals were selected for strontium (^87^Sr/^86^Sr), oxygen (δ^18^O), and carbon (δ^13^C) isotope analysis, prioritizing individuals with M2 and M3 pairs that were still in the maxillae or mandibles (five pairs). Details regarding the osteology and archeological context of these samples are given in text S1 and data S1. Samples were derived from 10 different burial pits, from trench 1/4 of the 1 Elverton Street excavation (ELV94) (*6*, *7*). Included in this sample set was a double-sample from Individual 7, with M2-M3 tooth pairs sampled from both the left mandible and left maxilla, respectively. The sampled horses were found to range from 3 to 4 years to over 20 years in age, with a majority of prime-aged adults between ages 8 and 14. By comparison, most of the horses from the wider Elverton cemetery were 10 to 20 years old, with just seven less than 5 years old (*6*). Enamel mineralization intervals in horses are 7 to 37 months for M2 and 21 to 55 months for M3, equating to periods of 2.5 and ~3 years, respectively (*18*). Because horse teeth wear progressively throughout life, the actual period represented in the isotopic data will be shorter in all adult individuals. In parallel with the isotopic analysis, teeth from 14 of the 15 sampled horses were sexed by aDNA screening at the Centre for Anthropobiology and Genomics, Toulouse. The procedure followed (*72*) and consisted of extracting ancient DNA from 200 to 480 mg of tooth powder, constructing triple-indexed DNA libraries, and processing shallow shotgun sequencing data through the Zonkey computational pipeline (*73*). Endogenous DNA preservation levels (0.74 to 94.1%; median = 4.6%) and the sequence data produced (408,391 to 946,136; median = 647,966) were sufficient to confirm the taxonomic status of the 14 remains as *Equus caballus*, identifying 9 males (stallions or castrated geldings) and 5 mares (data S1). Deeper sequencing aiming at whole-genome characterization is ongoing for those specimens showing relatively high DNA preservation, which may enable future investigation of phylogenetic affinities between the Elverton horses and modern breeds.

### Strontium isotope analysis

Strontium isotope analysis was undertaken following the procedures detailed in (*74*). For each tooth, an enamel-dentine strip approximately 3 mm wide running from the crown tip to the root was removed by hand using a diamond-encrusted circular saw. Strontium isotope ratios were measured along the midline of the enamel cross section from occlusal surface to root using laser ablation multicollector inductively coupled plasma mass spectrometry (LA-MC-ICPMS). A small length of dentine was also analyzed from a subset of teeth to estimate the bioavailable ^87^Sr/^86^Sr ratio at the burial site. Strontium isotope ratios were measured using a Thermo Scientific Neptune MC-ICP mass spectrometer coupled to a New Wave 193-nm Ar-F excimer laser ablation system (UP193FX) at the National Oceanography Centre, University of Southampton. Before data collection, the enamel and dentine targeted for analysis were pre-ablated to remove surface contaminants. The laser settings used for data collection were as follows: a laser beam diameter of 150 μm, repetition rate of 15 Hz, and tracking speed of either 15, 20, or 30 μm s^−1^. The ablated sample was swept from the laser cell using helium gas, which was then mixed with nitrogen and argon gas flows before entering the plasma ion source. The ^87^Sr/^86^Sr ratio was measured in static collection mode with an integration time of 1.049 s using a tuned mass spectrometer setup designed to minimize oxide production (monitored as ^254^(UO)+/^238^U+) through careful control of plasma conditions (*75*). Following data collection, an on-peak gas blank correction for ^86^Kr interference was applied to all masses, then ratios were corrected for mass fractionation using an ^86^Sr/^88^Sr ratio of 0.1194 according to an exponential mass fractionation law (*76*). ^89^Y was monitored as a proxy for rare earth element contamination and any data showing high concentrations were disregarded (*77*). Isobaric interference from ^87^Rb was corrected by using the natural ^87^Rb/^85^Rb ratio of 0.385617 (*78*).

Repeat analyses bracketing the archeological samples of an in-house enamel standard prepared from a pig fed exclusively marine foods and measured by thermal ionization mass spectrometry (TIMS) to 0.70906 showed an average offset of +149 ± 118 parts per million (ppm; 1σ) for the laser ablation analyses over the TIMS values. This is well within the precision of individual measurements of ~200 to 600 ppm and the total variation within the teeth of >23,900 ppm and is therefore considered irrelevant to our interpretation of the isotopes. The ^84^Sr/^86^Sr ratio fell close to the expected value of 0.0565 in almost all parts of the horse enamel profiles (text S1, section 2.3; and data S3). Any data that showed large deviations were discarded as part of the data reduction process and are not included here.

Enamel isotopic datasets were interpreted with reference to strontium and oxygen isoscape base maps for mainland U.K. (*12*, *15*, *24*) and continental Europe [see, e.g., (*16*, *31*, *47*, *48*)]. When differences were observed between the datasets, national base maps were preferred over global summaries, as the former typically incorporate more data and analyses specific to a particular region than do the international summaries.

### Oxygen and carbon isotope analysis

Enamel δ^18^O was measured as a marker of long-distance mobility, to assist with identifying horses potentially originating outside of western Europe—including the U.K., Iberia, France, and the Low Countries—which all show common δ^18^O_precipitation_ values of about −4 to −7‰ (*31*). Although precipitation in some regions bordering the Mediterranean Sea also shares these values, other parts of the continent, including central and eastern Europe and Scandinavia, show substantially lower values, and horses growing up in these regions should be potentially distinguishable from locally born British horses.

Sampling of enamel carbonates took place after the strontium isotope profiles had been measured. Our goal was to target enamel sections corresponding to the geographic origins of the horses and where possible before periods of notable mobility identified in the teeth based on the ^87^Sr/^86^Sr data. This strategy was designed to avoid measuring a broader average of δ^18^O_enamel_ across all the different geological (and potentially climatic) contexts where the horses had lived during tooth growth. Accordingly, we attempted to collect a single powdered enamel sample from each tooth drilled from a 30-mm interval and running parallel to the axis of growth (text S1 and fig. S5); an approximation of 1 year’s worth of growth when sampled from the upper, earlier-forming part of the tooth. Sampling locations were always located immediately adjacent to the strontium isotope sample region and correlated with periods of stability in the ^87^Sr/^86^Sr profile (data S2). In some teeth, the degree of fluctuations in the ^87^Sr/^86^Sr profile meant that a full 30-mm interval associated with a single geological context was not available for sampling. In these cases, we sampled across the maximum possible distance that correlated with a period of stable ^87^Sr/^86^Sr values, being at least 10 mm in every case (data S1 and S2). One tooth from Individual 5 was exceptionally sampled twice in an attempt to explore the strontium isotope variability already detected in the tooth. Tooth mineralization is a complex process, with the rate of mineralization in horses slowing progressively between the occlusal surface and the enamel-root junction (*17*). Despite standardizing the sampling methodology (including sampling location) between individuals as far as possible, some variation proved unavoidable, and while all our samples are time-averaged the actual temporal interval integrated within each carbonate sample will differ between individuals, representing a limitation of the enamel carbonate dataset.

For each tooth, a diamond-tipped burr was used to remove overlying cementum and clean the enamel surface before sampling. About 6 mg of enamel powder was then drilled using a 1-mm diamond-tipped drill bit from the selected sampling interval along the growth axis of the tooth cusp (text S1 and fig. S5). Enamel powders were pre-treated by soaking in 0.1 M acetic acid at room temperature for 10 min to remove any exogenous carbonates, and then rinsed with deionized water and freeze-dried (*79*). Between 0.5 and 0.6 mg of powder was then reacted with 106% phosphoric acid at 90°C using a Kiel IV carbonate preparation system at the National Oceanography Centre, University of Southampton. The resulting CO_2_ was analyzed using a Thermo Scientific MAT253 dual inlet isotope ratio mass spectrometer. Isotope ratio data are reported as δ values in per mil with reference to the VPDB isotopic standard for δ^18^O and δ^13^C. Replicates of IAEA (International Atomic Energy Agency) standards NBS18 and NBS19 as well as an in-house laboratory standard made from Upper Paleolithic mammoth tooth enamel show that the precision is better than ±0.2‰ for δ^18^O and better than 0.1‰ for δ^13^C.

### Statistical analysis

Data reduction and analysis, including calculation of statistics mentioned in the text (means, SDs, IQRs, SEs), were undertaken using Microsoft Excel and IBM SPSS Statistics 29.

## Supplementary Materials

### This PDF file includes:

Text S1 Figs. S1 to S11 Table S1 Data S2 and S3 Legend for data S1 References
