# Supplementary material for: Isotopic biographies reveal horse rearing and trading networks in medieval London
Source: Sci Adv. 2024 Mar 22;10(12):eadj5782. doi: 10.1126/sciadv.adj5782 (PMC10959406; doi:10.1126/sciadv.adj5782)
Supplement: Supplementary file 1 — Text S1 Figs. S1 to S11 Table S1 Data S2 and S3 Legend for data S1 References [file sciadv.adj5782_sm.pdf]

Science Advances
AAAS

Supplementary Materials for

Isotopic biographies reveal horse rearing and trading networks in medieval London

Alexander J. E. Pryor *et al.*

Corresponding author: Alexander J. E. Pryor, alex.pryor@exeter.ac.uk

Sci. Adv. **10**, eadj5782 (2024)
DOI: 10.1126/sciadv.adj5782

The PDF file includes:

Text S1
Figs. S1 to S11
Table S1
Data S2 and S3
Legend for data S1
References

Other Supplementary Material for this manuscript includes the following:

Data S1

Supplementary Text S1: Further information regarding the Elverton Street site horse assemblage and methods of analysis

Section 1: Further information

1.1 Sample provenance

The Elverton Street site is composed of two adjoining sites evaluated by Museum of London Archaeology during commercial rescue excavations in advance of redevelopment of the area for housing. Three trial trenches were excavated within the property of 1 Elverton Street (Site Code: ELV94), situated at the east end of Elverton Street, in the angle with Horseferry Road, in 1994. Three further trial trenches were excavated on the second site, 17 Elverton Street (Site Code: EVT95), which lies a little further west, in 1995–96. Both sites yielded large numbers of animal burial pits with an unusually high proportion of horse bones, including many articulated examples.

Figure S1: Elverton Street location map. (A) Location of the Elverton Street Excavations (in red), within William Morgan's 1682 Map of London (reproduced from (*7*); base map taken from "*Morgan's Map of the Whole of London in 1682", accessed from* http://www.british-history.ac.uk/no-series/london-map-morgan/1682/map. (B) Plan of 1 Everton Street excavations showing location of Trench 1/4 (reproduced from (*7*), and (c) Location of urban London within Britain and Western Europe.

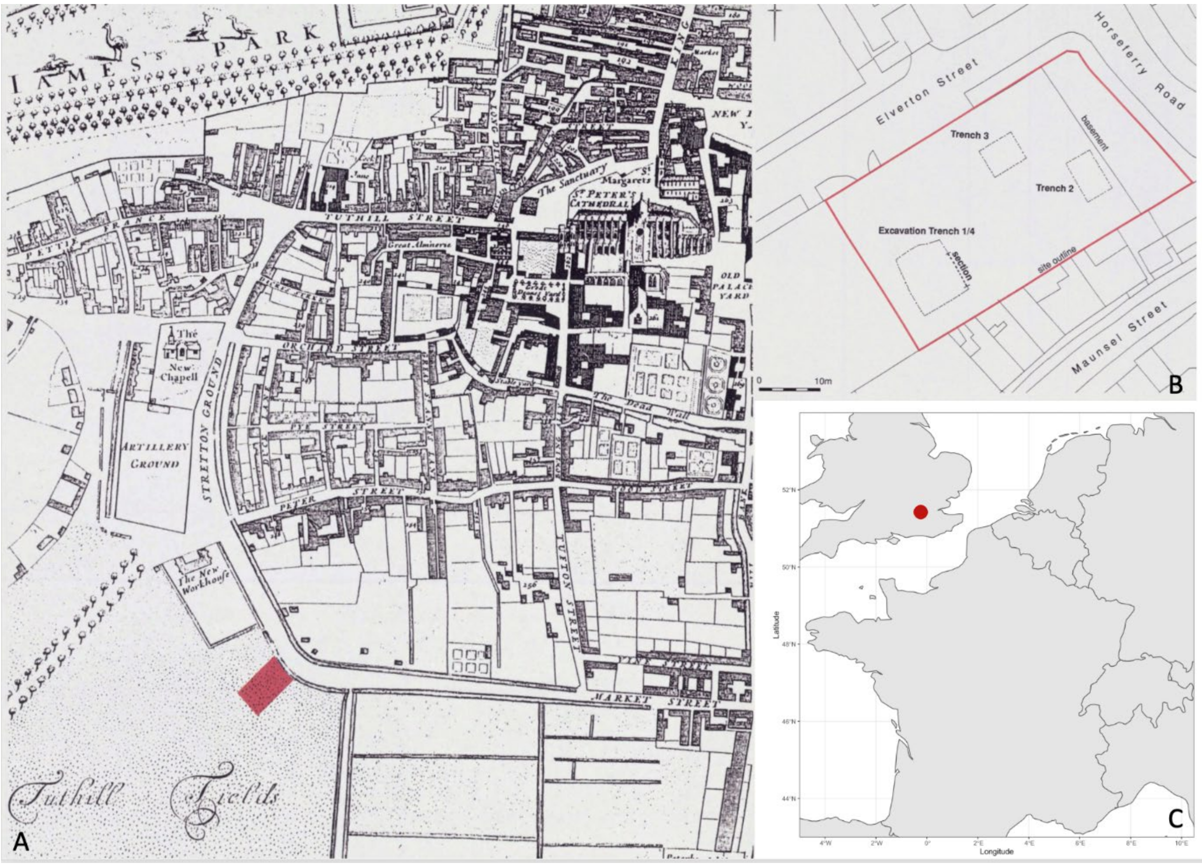

Figure S2: Site plan for Trench 1/4 (reproduced from (*7*)). Annotations indicate the location of individual samples selected for isotope analysis. Some sampled teeth originated from burial pits 7 and 10, which are not shown on this plan.

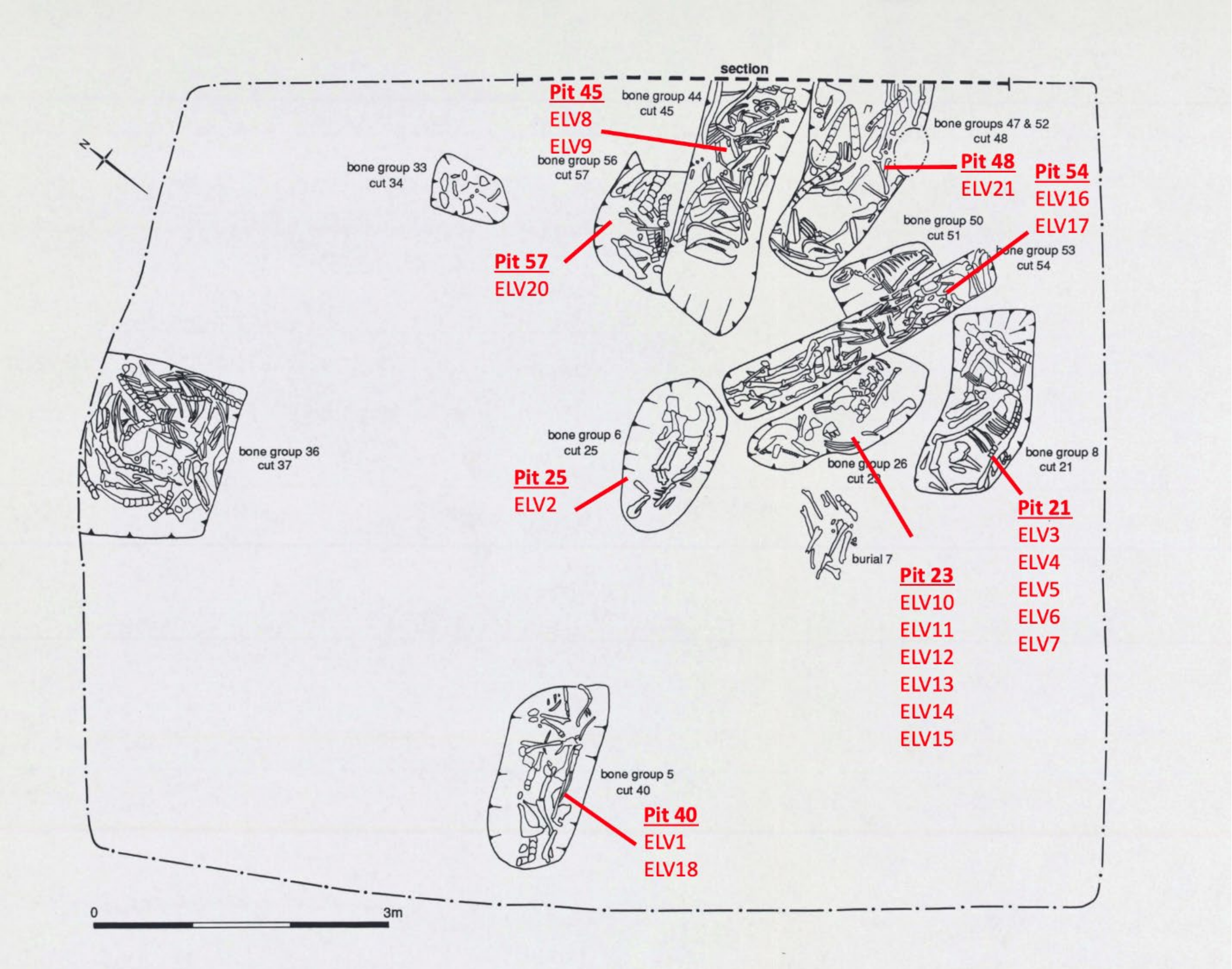

Fig 6 Plan of Burial Pits in Excavation Trench 1/4

1.2 Dating the Elverton horse assemblage

Twelve radiocarbon dates on individual horse bones are available for the Elverton cemetery including two dates measured in the 1990s (*6*) and ten further dates reported here (Table S1). The dated bones were recovered from burial pits 7, 21, 48 and 98. While both Roman and early medieval (1050–1300 AD) pottery was recovered from various of the burial pits at Elverton, the abraded nature of the finds suggest that they are residual and derived from the spoil excavated and then redeposited during the digging of the pits (*6*). The only artefacts directly associated with the horse remains were two horseshoes, of Clark's 'Type 4', attributable to the mid-14th to 16th centuries AD (*8*). Postmedieval activity at the site, as well as historical records of land use around adjacent Tutill Fields indicate that from 1605 the area of the site was the location of a sand quarry, which postdates all activity associated with the burial pits (*6, 7*). Several of the radiocarbon dates show bimodal calibrated probability distributions spanning an interval of almost 200 years, although a simple Bayesian model with a *terminus ante quem* of 1605 indicates a clear phase of cemetery use between AD 1425–1517 at 95.4% probability (Table S1; Figure S3; the Oxcal code used to generate the model is given below). The dated bones derive from four separate burial pits (pits 7, 21, 48 and 98) while the teeth analysed for strontium and oxygen in the present analysis derive from a total of ten different pits. Therefore, as a large number of burial pits remain undated, it is possible that the cemetery was used more widely throughout the late medieval and early Tudor period than the current dating evidence suggests (i.e. between the 14th –16th centuries).

Methods for measuring the ten radiocarbon dates reported here followed well-established standard procedures. Following mechanical cleaning, samples of 200mg of bone were crushed to ~1 mm powder and decalcified overnight at room temperature, in measured aliquots of 1N HCl calculated as just sufficient to dissolve the entire samples if all of the weight was hydroxyapatite.The samples were then washed with ultrapure MQ water, gelatinized at 60°C and pH 2 overnight, ultrafiltered to select the high molecular weight fraction (>30kDa) using precleaned Vivaspin 15 Turbo PES devices, and freeze-dried overnight. Aliquots of ~2mg of collagen were sealed under vaccum in quartz tubes with CuO oxidizer and Ag wire getter and combusted at 900°C for 3 hours, converted to graphite by hydrogen reduction with a Fe powder catalyst, and 14C was measured on a National Electrostatics 0.5MV spectrometer at the University of California Irvine's Keck Carbon Cycle AMS facility. Collagen aliquots of ~0.7mg were flash-combusted in tin foil cups and %C, %N, and δ^{13}C and δ^{15}N were measured with a Fisons NC1500 Elemental Analyzer coupled to a Finnigan Delta Plus isotope ratio mass spectrometer to a precision of <0.1‰ and <0.2‰, respectively.

Table S1: Radiocarbon dates from the Elverton horse cemetery

Date code	Element	Pit #	Sample name	^{14}C age (BP)	±	>30kDa collagen yield(%)	d^{15}N (‰)	d^{13}C (‰)	%N	%C	C/N (atomic)	Unmodelled (95.4% probability) From	to	Modelled (95.4% probability) From	To	A
Start (modelled)														1424	1465	
UCIAMS_278429	left metatarsal	48	#51 WEST01 Ages9174	385	15	10.8	6.1	-21.8	16.3	43.7	3.1	1452	1619	1452	1492	124.4
UCIAMS_278430	right metatarsal	7	#52 WEST11 Ages9184	395	15	8.9	5.9	-22.1	16.2	43.3	3.1	1448	1615	1450	1487	118.7
UCIAMS_278431	right metacarpal	21	#53 WEST12 Ages9185	425	15	6.2	5.7	-21.6	16.3	43.6	3.1	1439	1474	1442	1474	92.6
UCIAMS_278432	left metacarpal	21	#54 WEST13 Ages9186	400	15	5.9	5.6	-22.2	15.9	42.8	3.1	1446	1614	1448	1484	114
UCIAMS_278433	left metacarpal	21	#55 WEST15 Ages9188	370	15	10.4	5.5	-22.0	16.1	43.3	3.1	1457	1624	1455	1499	97.9
UCIAMS_278434	left metacarpal	48	#56 WEST34 Ages9197	365	20	12.7	6.6	-22.2	15.7	42.2	3.1	1457	1631	1454	1499	92.2
UCIAMS_278435	petrous	21	#57 WEST66 Ages9239	390	15	1.9	3.9	-22.7	15.2	42.2	3.2	1450	1618	1451	1491	123.1
UCIAMS_278436	petrous	21	#58 WEST68 Ages9241	395	15	4.0	7.5	-22.7	15.3	43.5	3.3	1448	1615	1450	1487	118.7
UCIAMS_278437	petrous	21	#59 WEST69 Ages9242	415	15	4.5	5.3	-22.3	15.3	42.9	3.3	1441	1481	1445	1477	100.7
UCIAMS_278438	petrous	7	#60 WEST72 Ages9245	430	15	3.8	4.7	-22.6	15.3	42.9	3.3	1437	1471	1441	1473	89.9
UB-4064 Pit 48		48		466	23							1420	1457	1435	1470	60
UB-4063 Pit 98		98		338	19							1481	1636	1456	1505	35.2
End (modelled)														1460	1516	
1605												...	1605.05			
														1604	1605	

Figure S3: Radiocarbon dates from the Elverton horse cemetery modelled in Oxcal (*9*, *10*) as a single phase with a *terminus ante quem* of 1605.

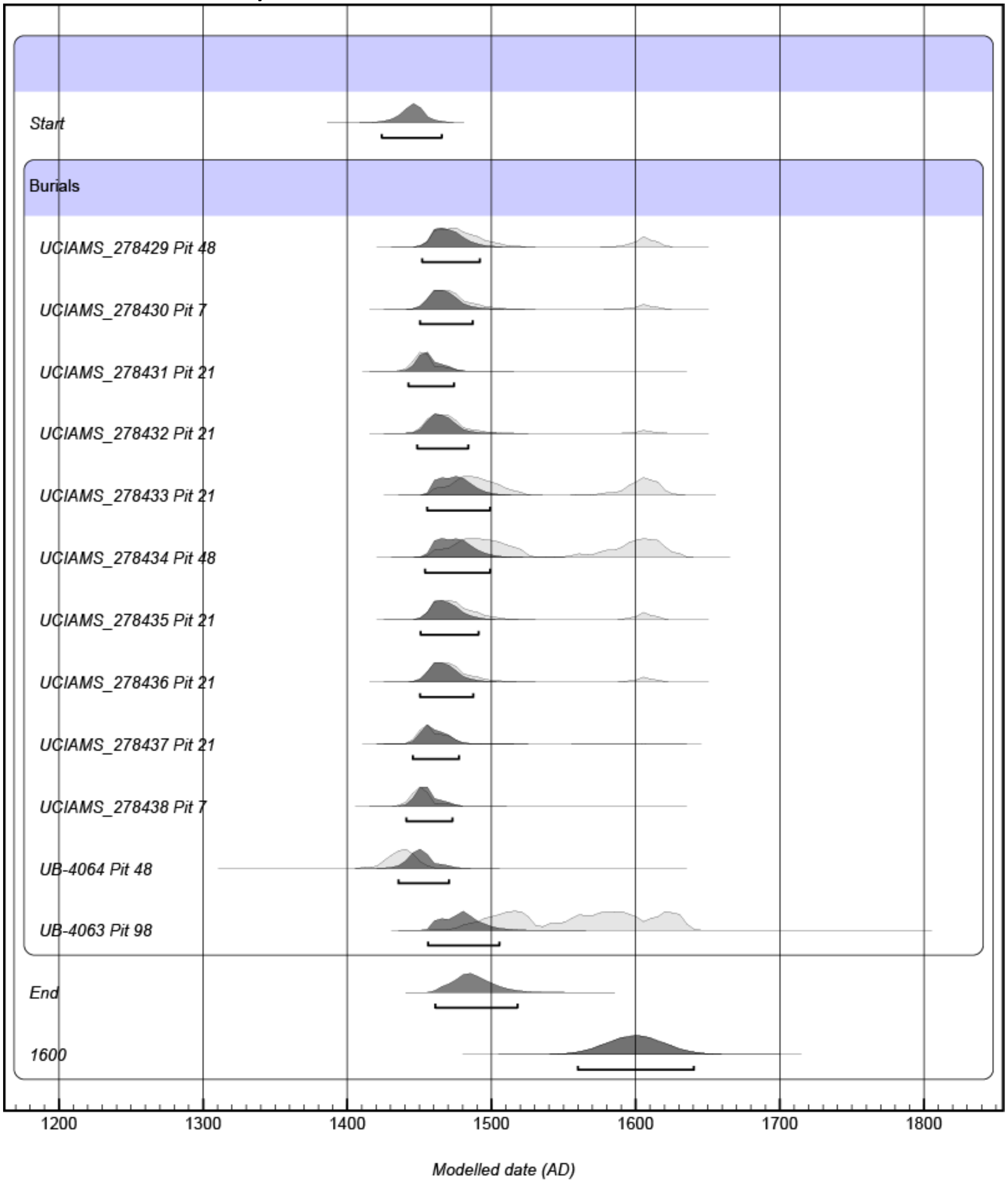

Model input used to calibrate the Elverton dates, using Oxcal version 4.4 and the IntCal calibration curve

```
Plot()
 {
  Sequence()
  {
   Boundary("Start");
   Phase("Burials")
   {
    R_Date("UCIAMS_278429 Pit 48", 385, 15);
    R_Date("UCIAMS_278430 Pit 7", 395, 15);
    R_Date("UCIAMS_278431 Pit 21", 425, 15);
```

```
   R_Date("UCIAMS_278432 Pit 21", 400, 15);
   R_Date("UCIAMS_278433 Pit 21", 370, 15);
   R_Date("UCIAMS_278434 Pit 48", 365, 20);
   R_Date("UCIAMS_278435 Pit 21", 390, 15);
   R_Date("UCIAMS_278436 Pit 21", 395, 15);
   R_Date("UCIAMS_278437 Pit 21", 415, 15);
   R_Date("UCIAMS_278438 Pit 7", 430, 15);
   R_Date("UB-4064 Pit 48", 466, 23);
   R_Date("UB-4063 Pit 98" , 338, 19);
  };
  Boundary("End");
  Before("1605", 1605);
 };
};
```

1.3 Zooarchaeological assessment of the horse assemblage

The Elverton horse assemblage was reanalysed to distinguish individual animals for sampling, and pathologies reassessed to interpret in-life use. Teeth were measured for age-at-death based on cheek tooth crown height (*80*) and bones were measured for size (*68*). Two horses were under six years (horses 10, 14), five between 6–10 (2, 12, 13, 15, 16), four between 10–15 (1, 4, 7, 11), and four were over 16 years (3, 5, 6, 8) (Data S1). Withers heights, calculated from Greatest Length (GL) of long bones (*69*) ranged from 1232.60–1617.03 cm (12–15.3 hands high, hh), averaging 1392.28 cm (13.2 hh), relatively higher than the average from late medieval horses (13 hh) (*58*), including three of the tallest recorded horses from late medieval England (1.47–1.60 m, 14.2–15.3 hh), from the over forty horses where height was measurable. The larger-than-average withers heights calculated from the Elverton horse bones is clearly visible (Figure S4) and also highly significant when compared against bones from both other late medieval horses from an urban-only context (p = 0.000238 (Mann-Witney U test, with Monte Carlo permutation p = 0.0002)), and also horses from late medieval urban and rural contexts combined (p = 6.4948^{-9} (Mann-Whitney U test, with Monte Carlo permutation p = 0.0001))(data from (*4*)). Though taller horses were present at Elverton, there was a high degree of size variability, with 38.1 cm difference in height from the smallest to largest horses. Slenderness indices of metapodia (SD/GL * 100) reiterate this variation, 10.33 to 16.73 on third metacarpals (average 14.77) and on third metatarsals 10.22–19.39 (average 12.24), also above average for both elements for late medieval horses (*58*). In summary the Elverton Street horses were on average notably taller and more robust than horses from other late medieval assemblages in England.

Figure S4: Withers heights calculated from Elverton horse bones compared against a reference dataset of other late medieval horse bones and also those derived only from urban contexts, using data from (*4*).

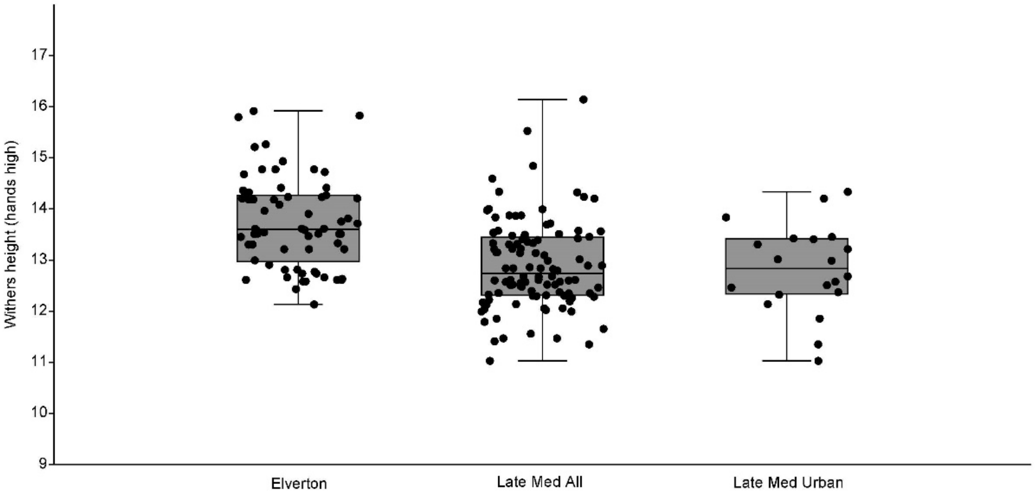

The Elverton horses suffered a number of pathologies, revealing relatively hard work, with ossification of the interosseous ligaments of the metapodials (splints) the most common (N=19), followed by bone spavin (fusion of the hock joints, N=16), fusion of the vertebrae of the lower back, N=9 (8 lumbar, 1 thoracic), osteoarthritis (OA) of various joints hind- and forelimb (N=12), ossified haematomas of the third metatarsals (N=2) and tibia (N=1), OA of the atlantoaxial joint (2), fusion of proximal interphalangeal (N=1) and distal interphalangeal joints (N=1), and a healed fractured rib.

Fusion of the vertebrae of the lower back is related to the weight bearing and concussion of the rider on the horse's back (*81*). Horses from other medieval sites in London show varying degrees of fusion of the lumbar vertebrae (*82*). That the lumbar vertebrae are more frequently affected in medieval populations, rather than the thoracic vertebrae, may be related to saddle design of the 15th century, which shifts the rider's weight more toward the lumbar spine more than earlier saddles.

Bit wear was identified on the second lower premolars (LP2s) of horses 1, 5, 8 and 10 (*83*). Horse 5 (male) has bit wear that is suggestive of heavy use of a curb bit. The entire anterior cusp of the tooth is worn away. This unusual wear is indicative of curb bits used on elite, especially war and tournament horses, after the 14th century (*71*). Bit wear on two of the mares (8, 10) further corroborates the idea that mares were valuable for use under saddle or in harness and breeding.

1.4 Sampling strategy

Figure S5: Tooth ELV16 sampled for strontium, oxygen and carbon isotope analysis. The strontium sample comprises a single enamel slice running from occlusal surface to tooth base. Each strip was then turned on its side to expose the enamel cross-section, which was then analysed. The powdered enamel carbonate sample was subsequently drilled from a 30 mm strip immediately adjacent to the strontium column, after removal of the overlying cementum. Further description of the sampling procedure is given in the Methods.

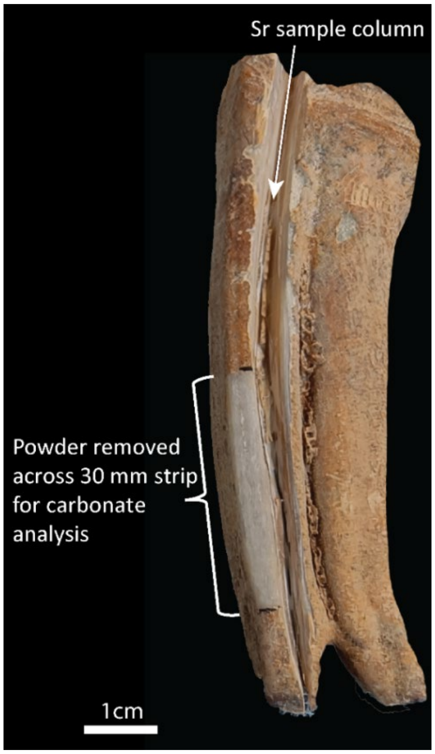
Sr sample column
Powder removed across 30 mm strip for carbonate analysis
1cm

Section 2: Results – further information

2.1 Strontium isotope ratio measurements of dentine and cementum

Strontium isotope measurements of tooth dentine and cementum were collected along lines approximately 1–4 mm long placed at random in 12 teeth. Two teeth were analysed in more than one location. The results clustered mostly between 0.709 and 0.710 and showed relatively flat lines as expected for dentine and cementum tissues that have reached isotopic equilibrium with strontium in the burial environment (Figure S6). Cementum measurements in two teeth, ELV17 and ELV18, showed more radiogenic values than all others. These individuals were found in different pits, and could potentially indicate that the burial matrix near these teeth had a different composition compared to that surrounding all other analysed teeth. For example, teeth from these individuals may have been buried deeper in a lower-lying geological layer characterised by a $^{87}Sr/^{86}Sr$ ratio that is distinct from overlying sediments. This seems unlikely, however, as ELV16 also comes from the same pit as ELV17 and produced dentine values consistent with others from across this site. Furthermore, sediments with values > 0.710 are not predicted to occur in the London region and are not known from previous studies (see main text). A more likely explanation is therefore that cementum tissues in ELV17 and ELV18 were only partially equilibrated with local sediments, and it is noteworthy that the enamel of these individuals was among the most radiogenic of all samples measured requiring a very large change in $^{87}Sr/^{86}Sr$ to reach local values. Cementum data from ELV17 and ELV18 was therefore omitted when calculating the local baseline value.

Strontium isotope ratios in dentine and cementum from the ten remaining teeth (total of 12 laser tracks) indicated a local bioavailable $^{87}Sr/^{86}Sr$ range at the burial site spanning 0.709064 to 0.710008 (n = 1,315 individual integrations; $\bar{x}$ = 0.709564; σ = 0.000261; the median was 0.709620; the interquartile range was 0.000488; the total dataset spanned the range 0.709064–0.710008).

Figure S6: Strontium isotope profiles from short tracks measured in tooth dentine and cementum of the Elverton horses. The $^{87}Sr/^{86}Sr$ data are shown as a 10-pt mean running average of the individual laser ablation measurements.

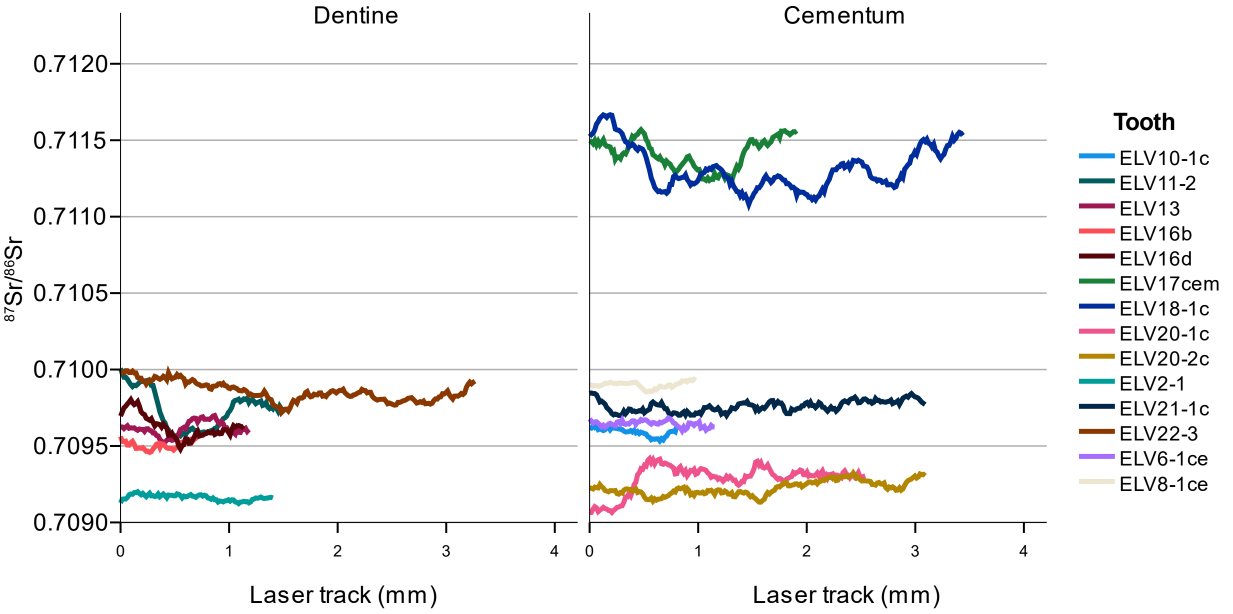

2.2 Defining the 'local' London range

The City of London rests on bedrock geology belonging to the Thames Group formation, dating to the Eocene epoch and comprising marine-derived clay, sand, silt and gravel (*11*). In locations close to the River Thames, including the Elverton Street cemetery which is located less than 800 m from the present-day

river channel, this bedrock geology is capped by quaternary alluvial deposits. $^{87}Sr/^{86}Sr$ ratios in dentine and cementum from ten Elverton horses indicate a local strontium isotope range at the burial site spanning 0.709064 to 0.710008. This range matches closely with previously reported bioavailable strontium ranges for the London region. People born and raised in the Greater London area dating from the Roman to post-medieval eras have previously been found to have $^{87}Sr/^{86}Sr$ ratios mostly within the range of 0.709–0.710 (*13, 14*), correlating with the expected $^{87}Sr/^{86}Sr$ range for the Eocene bedrock based on measured plants (*12*) and modelling studies (*16*). A small but distinct population subset has also been recognised with less radiogenic $^{87}Sr/^{86}Sr$ ratios between 0.708–0.709 (*13*). London is bounded on three sides by Cretaceous-period chalklands which are present to the south (14 km from Elverton), west (30 km from Elverton) and north (28 km from Elverton), with expected $^{87}Sr/^{86}Sr$ ratios between 0.7079–0.7086 (*12, 15*). This has led to a suggestion that lower-than-expected $^{87}Sr/^{86}Sr$ ratios in Londoners could reflect consumption of foods (particularly grain) produced in the London hinterland on chalklands and transported to the city for sale and consumption, resulting in final enamel values slightly less radiogenic than could be produced based on the London clays alone (*13*).

When interpreting the Elverton data the local range was based only on values for the city of London itself, and excluding the surrounding chalk deposits. This was done to ensure that potential horse mobility between the city and hinterlands would be distinguishable, although the impact of foods brought into the city for consumption is clearly an important consideration when interpreting the Elverton horse data, just as for the medieval humans previously analysed. In the current analysis, the local range for Elverton was therefore pegged to 0.709064–0.710008, equating to the min-max values recorded in the tooth cementum and dentine.

2.3 Enamel strontium results - Data quality control

The laser ablation measurements of Elverton horse tooth enamel demonstrated very low Y content, and overall excellent reproducibility in all samples of the expected $^{84}Sr/^{86}Sr$ ratio of 0.0565 (*84*). This is shown statistically in Data S1, and in dataplots of the full, reduced data output for individual teeth in Data S3. Occasional instances are apparent where short sections of some enamel profiles have diverged slightly from the expected $^{84}Sr/^{86}Sr$ ratio (e.g. ELV15 between 30–32 mm from the enamel root junction (ERJ)). Where the $^{87}Sr/^{86}Sr$ ratio shows no distinguishable difference from the enamel immediately above/below, the profile sections were retained. Where unexpected $^{84}Sr/^{86}Sr$ ratios coincided with increases in ^{89}Y, ^{88}Sr, and/or distinct changes in the $^{87}Sr/^{86}Sr$ ratio, these data were rejected during data reduction and cleaning and are not reported here (e.g. ELV10 between 0–3 mm from the ERJ).

The enamel profile of one tooth, ELV22 showed unexpected fluctuations in the $^{84}Sr/^{86}Sr$ ratio (Data S3). The profile for this tooth was collected during two separate analytical sessions in December 2021 (0–14 mm from the ERJ) and in December 2022 (14–58 mm from the ERJ) respectively. The problem likely relates to the formation of calcium dimers and argides during the December 2022 analytical session, which are known to particularly impact the $^{84}Sr/^{86}Sr$ ratio but have no effect on the accuracy of $^{87}Sr/^{86}Sr$ ratios outside of their normal level of precision (*75, 77*). We note that throughout the profile of ELV22, the presence of Y remains low (indicated by very low voltage readings), the total absolute deviation of $^{84}Sr/^{86}Sr$ from the expected value remains small throughout the sample (indeed, the total deviation remains less than that observed in parts of other Elverton teeth), while the measured $^{87}Sr/^{86}Sr$ ratio shows no evidence of co-variance with the recorded $^{84}Sr/^{86}Sr$ ratio. We can also report that measurements of the in-house pig enamel standards bracketing the analyses remained within usual tolerance limits. Therefore, following these checks, $^{87}Sr/^{86}Sr$ data from ELV22 were retained.

2.4 Evidence for periods of sedentism in some Elverton horses

Horses 2 (M3), 4 and 8 (M2–M3 pairs), all show periods of exceptional stability in their $^{87}Sr/^{86}Sr$ profiles which stands out as unusual when compared with profiles from other Elverton individuals (Data S2). These highly stable sections indicate either that mobility occurred but was restricted to very isotopically homogenous parts of the landscape, or that Horse 2, 4 and 8 were essentially immobile (e.g. confined to

specific pastures) during certain phases of their life. Both these possibilities require careful consideration, yet we argue here that the highly stable $^{87}Sr/^{86}Sr$ profile sections represent genuine immobility. Horse 8 is a 20+ years old female whose teeth showed a high degree of wear. The strontium data from Horse 8's M2–M3 pair therefore describe two relatively short snapshots in time ~6–12 months long and approximately 18–24 months apart, with a stable $^{87}Sr/^{86}Sr$ profile just within the upper end of the local London range. It is unlikely that any geological context could be sufficiently homogenous as to create the profile observed in horse 8 if movements were occurring, even if mobility was limited to a 1-day's walking radius around a central location. Horses typically consume 2–3 % of their bodyweight in food and between 20–80 litres of water daily depending on activity rates, pregnancy, or lactation (*39*), and even small-scale movements are likely to have resulted in small shifts in dietary sources of food and water being consumed, expressed as detectable fluctuations or wiggles in the $^{87}Sr/^{86}Sr$ profile. Such wiggles are indeed observed in Horse 8 (and Horses 2 and 4) and most likely reflect small changes in dietary Sr intake, but they are smaller in magnitude than those seen in other individuals.

The highly stable $^{87}Sr/^{86}Sr$ profile from Horse 8 can be directly compared with similarly aged individuals Horses 5 and 6, whose M2–M3 pairs displayed comparable levels of tooth wear but are both males. Both these male individuals show markedly more variation in their equally short $^{87}Sr/^{86}Sr$ profiles, providing snapshots in time up to two years apart as seen in Horse 8. A single M3 from Horse 3, another 20+ years male, also shows the same pattern of increased variability relative to the female Horse 8. Any inferences drawn from these comparisons must remain tentative because it is obvious that the Elverton assemblage comprises horses of diverse origins, and the degree of geological variability, and hence strontium isoscape variability, near where different individuals grew up remains unknown and may have differed in each case; the magnitude of noise in the laser ablation data — manifested as small fluctuations in the $^{87}Sr/^{86}Sr$ profile — can also potentially vary between samples due to factors such as enamel strontium concentration, beam intensity and various aspects of tuning in the mass spectrometer setup (*75*). Nevertheless, sections of the $^{87}Sr/^{86}Sr$ profiles from Horses 2, 4 and 8 are highly stable compared with both other Elverton horse data and also laser ablation analyses of other species at other sites, and therefore stand out as unusual. Thus, we consider the highly stable $^{87}Sr/^{86}Sr$ profiles observed in horses 2, 4 and 8 to be incompatible with mobility even within a 'daily home-range', and instead argue that these plateaus in the $^{87}Sr/^{86}Sr$ profiles should be understood as periods of immobility (e.g. restriction to specific pastures) during certain phases of the horses' lives.

2.5 Enamel oxygen and carbon

Calibration of $\delta^{18}O_{carbonate}$ to $\delta^{18}O_{drinking\ water}$

Horse $\delta^{18}O_{carbonate}$ data were first converted from the Pee Dee Belemnite (PDB) to the Vienna Standard Mean Ocean Water (VSMOW) reference scales, and then to an equivalent enamel $\delta^{18}O_{phosphate}$ value:

Equation 1:

$$\delta^{18}O_{VSMOW} = 1.03092\ \delta^{18}O_{PDB} + 30.92$$

Kim *et al*. 2015 (*85*)

Equation 2:

$$\delta^{18}O_{phosphate} = 0.98\ \delta^{18}O_{carbonate} - 8.5$$

Iacumin *et al*. 1996 (*86*)

The isotopic composition of drinking water / local precipitation consumed by the Elverton horses during tooth growth was estimated using two different relationships:

Equation 3:

$$\delta^{18}O_{enamel} = 0.71\ \delta^{18}O_{precipitation} + 22.61$$

Delgado Huertas *et al*. 1995 (*29*)

Equation 4:

$$\delta^{18}O_{enamel} = 0.62\ \delta^{18}O_{precipitation} + 22.14$$

Pederzani *et al*. 2021 (*30*)

The more recent equation proposed by Pederzani et al. (2021) is based on an almost identical calibration dataset to that used by Delgado Huertas (1995), with the exception that it adds two further calibration datapoints based on data from recent Mongolian horses presented in (*87*). Whilst it is good practice to use all available relevant data for calculating quantitative calibration relationships, the two additional datapoints are outliers in the calibration dataset and therefore exert a disproportionate amount of influence over the calculated relationship (Figure S7). They also fall well outside the range of enamel and precipitation values relevant for the present study. For $\delta^{18}O_{precipitation}$ values between ~–9 and +1 ‰ the two equations produce results that are within margins of error (Figure S8). The Pederzani et al. equation also produces systematically lower estimates of $\delta^{18}O_{precipitation}$ for precipitation values of ~–6 ‰ or lower due to a reduced slope relative to the Delgado Huertas et al. equation. As there is currently no way to assess whether one equation is more representative or 'correct' than the other, we have calibrated our Elverton horse data using both equations and considered both sets of outputs as part of our analysis.

Figure S7: Calibration data converting horse $\delta^{18}O_{phosphate}$ to $\delta^{18}O_{precipiation}$ (Equation 4). The two datapoints displayed in orange indicate the extra data from (*87*) used to generate Equation 4 by (*30*).

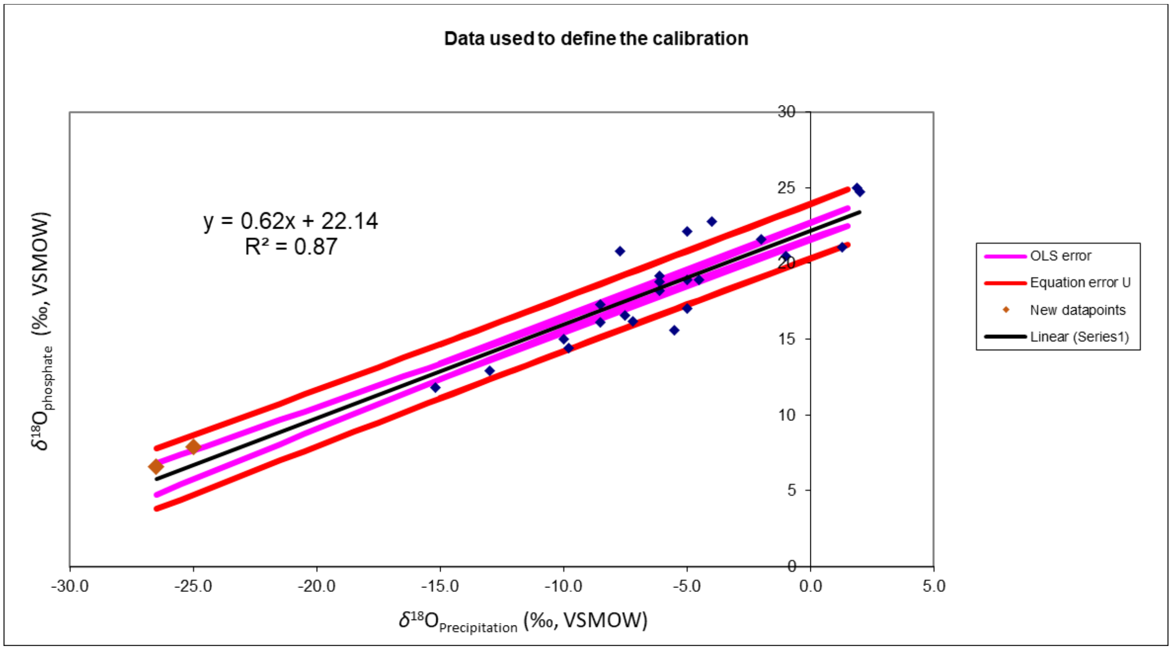

Figure S8: Plot comparing the $\delta^{18}O_{precipitation}$ - $\delta^{18}O_{enamel}$ quantitative relationships for horses given by (*29*) and (*30*). The error envelope for Equation 4 (30) is also marked.

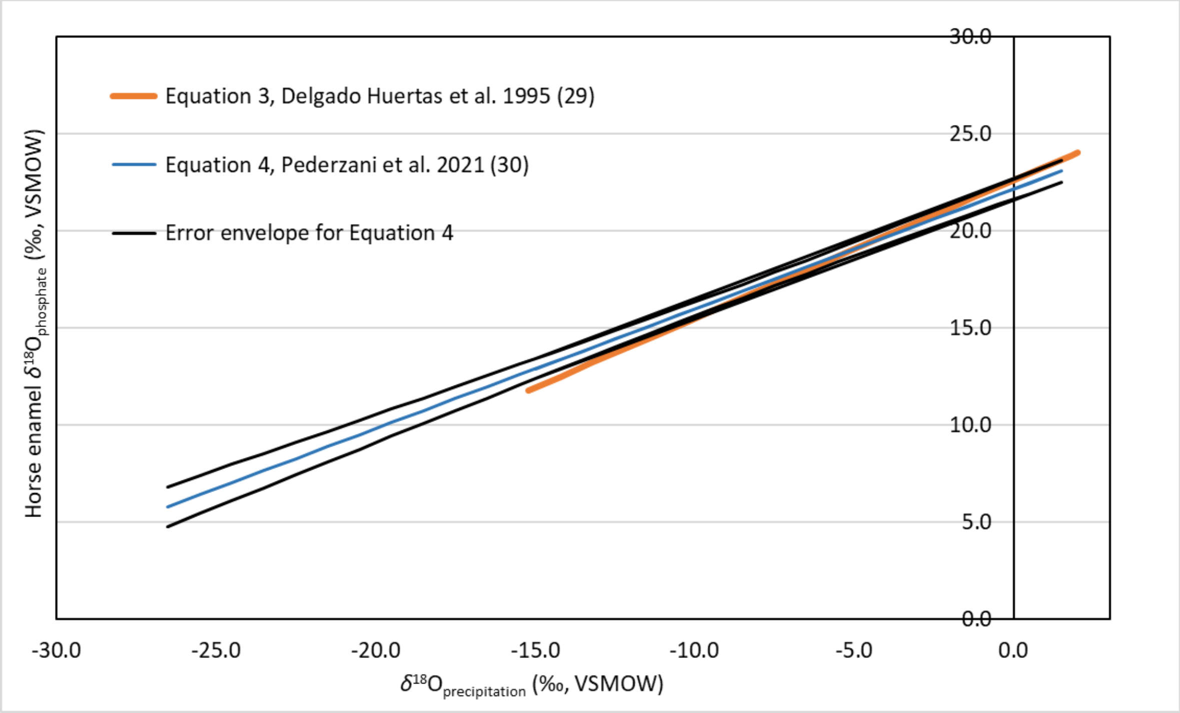

Enamel $\delta^{13}C$

Elverton horses had mean $\delta^{13}C$ of –13.3 ‰ (2σ = 0.9 ‰), indicating that all individuals consumed a pure C3 diet over the approximately one year of enamel growth captured by the samples. Outlier analysis using a threshold of median ± 1.5* IQR for identifying outliers indicated that no individuals are statistically outlying in $\delta^{13}C$, however individual 16 is notably enriched and individuals 12 and 14 are marginally depleted in ^{13}C relative to all other horses (Figure S9). Interestingly, Horse 14 also has high $\delta^{18}O$, and is the only Elverton horse potentially born and raised on the chalklands surrounding London or in south or east England more generally. Meanwhile, Horses 12 and 16 both produced $\delta^{18}O$ values showing they originated in relatively cold places, but clearly had very different diets, and it is possible that Horse 16 lived in a more arid environment than all other analysed horses (*88*).

Figure S9: Enamel carbonate $\delta^{18}O$ and $\delta^{13}C$ data for the Elverton horses. Datapoints are colour-coded and labelled according the individual horses. Lines indicate datapoints measured on teeth from the same individual.

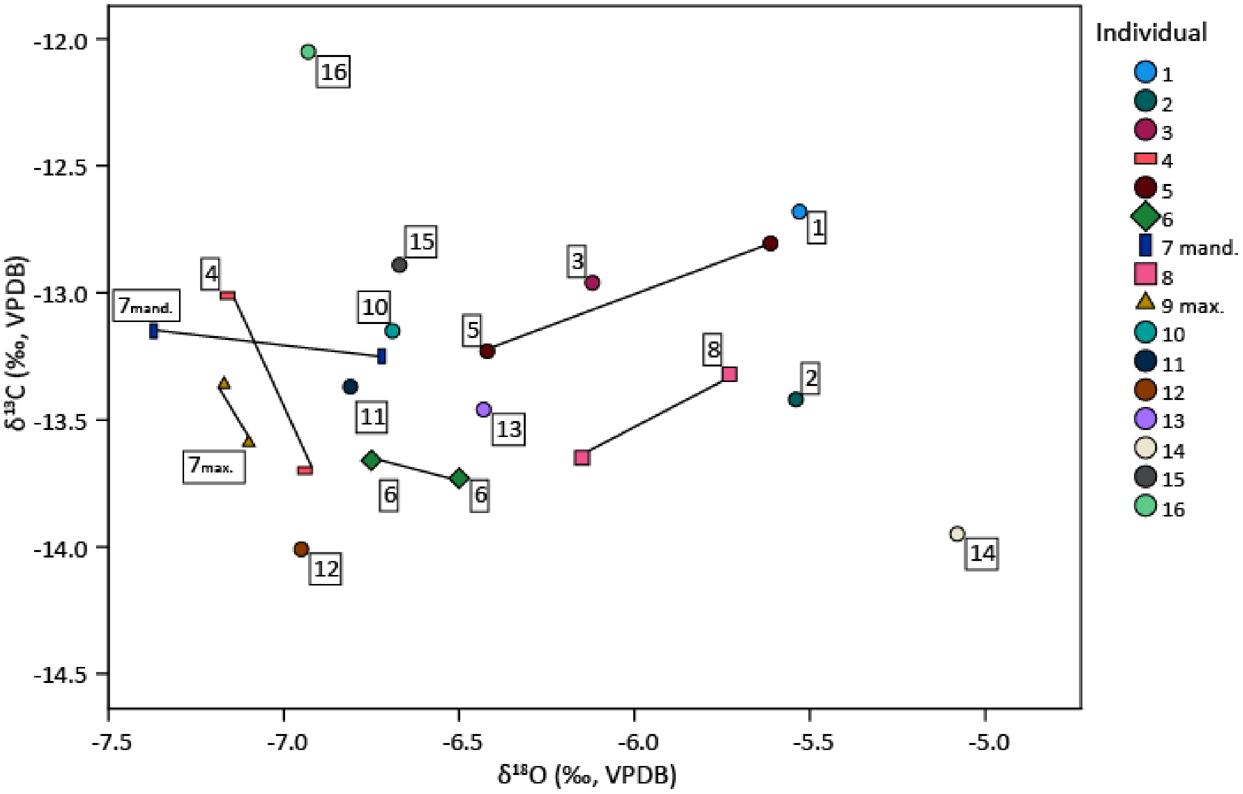

Figure S10: Strontium and oxygen isotope data for 15 Elverton horses showing $\delta^{18}O_{drinking\ water}$ estimates calculated using Equation 4 proposed recently by (*30*). Blue bars indicate the strontium isotope range measured in each individual. Background shading correlates to the strontium isotope zoning used in Figure 6A of the main paper.

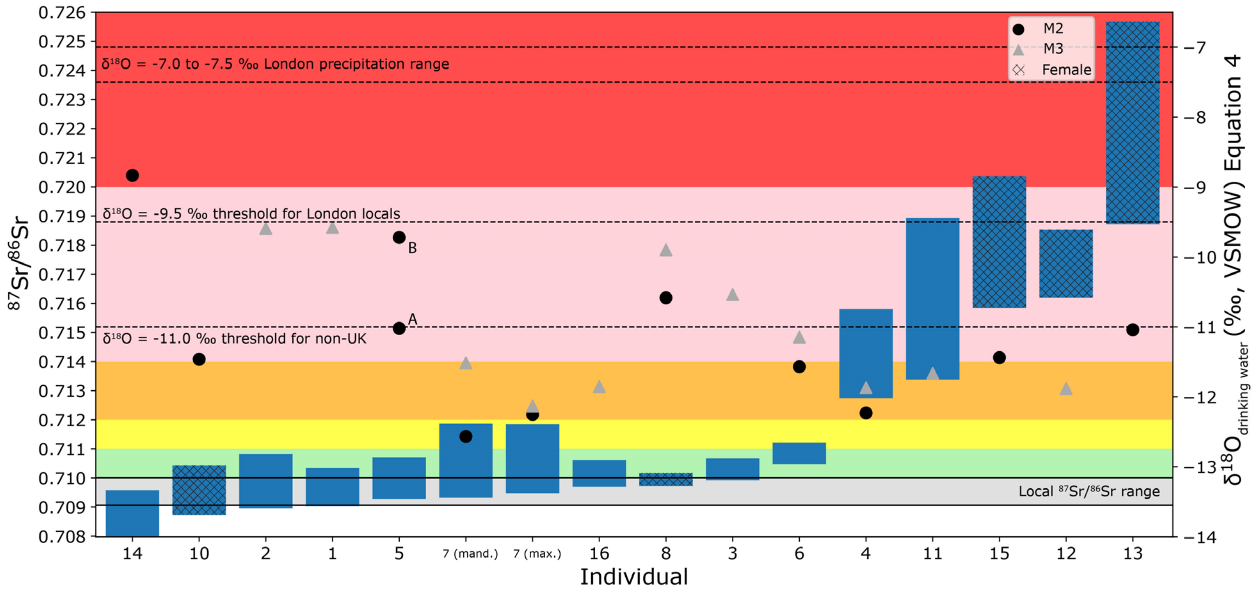

Figure S11: Strontium and enamel carbon isotope data for 15 Elverton horses. Blue bars indicate the strontium isotope range measured in each individual. Background shading correlates to the strontium isotope zoning used in Figure 6A of the main paper.

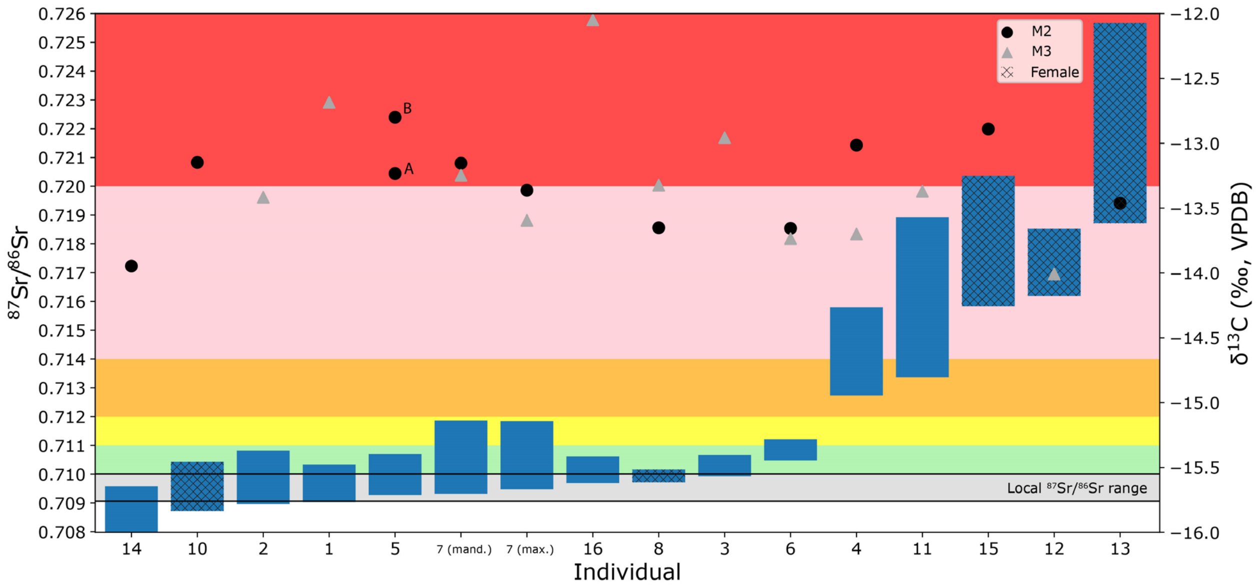

Section 3. Summary of results and interpretation for each Elverton horse

Horses 1 and 2 are each represented by a single M3 with $^{87}Sr/^{86}Sr$ profiles falling mostly within the local London range and almost identical $\delta^{18}O_{drinking\ water}$ values just about consistent with deriving from the local London range (Equation 3). Horse 1 was clearly mobile throughout the period captured via sampling (~2.5–4.5 yrs of age), with strontium isotope values consistent with origins and movements within London, or southern Britain more generally. Horse 2 shows a different pattern, being restricted to one very specific location until around 3.5 years of age, after which the $^{87}Sr/^{86}Sr$ profile rises and plateaus briefly indicating that the horse had moved to a different location just outside the local London Sr-isotope range. A sharply oscillating saw-tooth $^{87}Sr/^{86}Sr$ profile is then observed for the last 6 mm of enamel growth, indicating continuous regular movements between chemically-distinct locations. Such a pattern might indicate a horse confined to stables or single paddock for early-years training, before entering a life of service characterised by regular and habitual mobility between geographically varied locations. In summary, the data are therefore consistent with a locally-born and raised individual that subsequently moved outside the city, returning periodically once its working life had commenced. Nonetheless, given the isotopic equifinality between London and many areas of southern Britain respectively, non-local origins, mobilities and a later arrival to London still cannot be definitively excluded for horse 2 or indeed horse 1 too.

The M2–M3 pair from **Horse 4** produced $^{87}Sr/^{86}Sr$ profiles that occupy a clearly different and distinct range compared to all other investigated Elverton horses. Based on tooth growth rates of (*18*), Horse 4 remained essentially immobile until approximately 3.5 years of age after which this individual started to encounter several different and chemically distinct geologies. While Horse 4 is highly unlikely to have grown up in Britain, its origins are also clearly different to all other imported horses, while still coming from a region experiencing rainfall with markedly lower $\delta^{18}O$ values compared to Britain.

Horses 3, 5, 6 and 10 have $^{87}Sr/^{86}Sr$ profiles mostly above 0.7092 and entirely below 0.7113, values which can be found widely both within the UK and abroad and while they are therefore not geographically specific, they are consistent with British origins. Horses 3, and 5 have estimated $\delta^{18}O_{drinking\ water}$ in the range –9 to –11 ‰ (i.e. within 2‰ of the lower range limit for UK rainfall) regardless of whether Equation 3 or Equation 4 are used. Meanwhile, for horses 6 and 10, calibrating their $\delta^{18}O$ results with the more conservative Equation 3 produces drinking water estimates within 2 ‰ of the UK range while Equation 4 produces $\delta^{18}O_{drinking\ water}$ estimates below the –11 ‰ threshold, and would therefore be considered potentially of non-British origins. Parsimoniously, these four individuals are plausibly of non-London British origin, but origins internationally cannot be ruled out and become more likely if $\delta^{18}O_{drinking\ water}$ is calculated using Equation 4 instead of the more conservative Equation 3.

Horse 8 has a strontium profile falling at the upper limits of the local London range and estimated $\delta^{18}O_{drinking\ water}$ around the lower limits of the expected range, and may be of local origins. The strontium data from Horse 8 describes two relatively short snapshots in time ~6–12 months long and approximately 18–24 months apart, with a stable $^{87}Sr/^{86}Sr$ profile just within the upper end of the local London range. Given the similarity between the M2 and M3 data, it is reasonable to suggest that Horse 8 remained immobile during the first 4–5 years of life, as might have occurred for a stabled horse or pastured breeding mare living on a stud with minimal mobility or dietary variation needed beyond rich pasture (*89*).

Horses 7 and 16 produced strontium profiles well within the UK range, but both consumed drinking waters with low $\delta^{18}O$ very unlikely to be of British origin (~–11 to –11.6 ‰ using Equation 3; –11.5 to –12.6 ‰ using Equation 4). Meanwhile, the $^{87}Sr/^{86}Sr$ profile for Horse 7 records a series of movements throughout the tooth growth interval across a range of different lithologies. The combined Sr and $\delta^{18}O_{enamel}$ data are suggestive of cool-climate origins somewhere in continental Europe, for example in southwest Norway, the Alps, the Bohemian Massif uplands or the Carpathian Mountains. Then, between approximately 2.5 and 4+ years of age, the $^{87}Sr/^{86}Sr$ profiles record at least four mobility events between different lithologies,

ending in a location isotopically indistinguishable from London with $^{87}Sr/^{86}Sr$ below 0.710, from ~4.5 years old. The distances covered during these mobility events are unknown and may relate either to mobility close to the birth location, mobility within continental Europe in the period prior to Horse 7 being transported to London, or conceivably the isotopic data may have recorded the entire journey from birth location, through various trading networks to the moment when Horse 7 arrived in London, followed by a period of initial mobility within or around the city itself. Whichever, the $^{87}Sr/^{86}Sr$ profiles attest that Horse 7 visited a number of different lithologies during tooth growth, potentially including repeated visits to the same location months apart, but certainly not including any return to its place of birth.

Horses 11, 12 and 15 produced highly radiogenic signatures almost entirely above 0.715 and rising as high as 0.720. In the UK values in this range are restricted almost entirely to the Cairngorm Mountains in Scotland or the Cornubian batholith in southwest England. As for Horse 13, neither of these possibilities seems likely or even possible given the consistently highly radiogenic signatures throughout the horse profiles, combined with rather low $\delta^{18}O_{\text{drinking water}}$ values. Possible source locations for these individuals are therefore south Sweden, Lithuania, the Pyrenees mountains, or more radiogenic parts of the Western and Central Alps, the southwest Bohemian Massif or the Carpathian Mountains, but this is not an exhaustive list. It is also conceivable that horses 11, 12 and 15 (found in 3 different burial pits) all came from the same source region, and even from the same farm or breeder or stud, given the isotopic similarity of these individuals. In this context it is noteworthy that Horse 15 (a female) shows evidence of a mobile lifestyle throughout the period of M2 growth (18 months to ~3 years), while horse 12 (also a female) shows a more 'classic' pattern of a rather stable $^{87}Sr/^{86}Sr$ profile until at least three years of age, after which more evidence for mobility is seen, likely reflecting a horse turned out on pasture before entering a period of more active training and use.

Horse 13 produced the most radiogenic strontium values yet detected in the British archaeological faunal record, ranging up to 0.72567 and only rarely falling below 0.7220. British biosphere values this high have only previously been found in parts of northeast Dartmoor, centred around granite outcrops enriched in rubidium which have produced values between 0.7143–0.7287. SW Britain can, however, be ruled out given that Horse 13 consumed drinking waters highly depleted in ^{18}O.

Rather, based on the highly distinctive Sr profile and low $\delta^{18}O_{\text{drinking water}}$ values, south Sweden, south Finland or the Alps seem the most plausible source locations for Horse 13, from both chemical and cultural perspectives (*50, 51 and references therein*). In particular, crystalline rocks in the Western and Central Alps are known for producing highly radiogenic signatures (*48, 52*), and the area was active during the medieval period as a region for horse breeding and trading (*53-55*). It is noteworthy that the M2 $^{87}Sr/^{86}Sr$ profile of Horse 13 is relatively stable during the first half of tooth growth (approximately 6–18 months of age (*18*)) but then becomes much more variable in the latter part (approximately 18 months to 3 years of age). This conforms to the 'classic' pattern, whereby a young horse is turned out on pasture in the early years of their life to mature before more entering a period of more active training and use beginning around 2–3 years of age.

Horse 14 produced the highest $\delta^{18}O$ and one of the lowest $\delta^{13}C$ measurements in the dataset, suggestive of relatively warm and wet climates compared to those experienced by other horses found at Elverton. Horse 14 is also the only individual to show $^{87}Sr/^{86}Sr$ values consistent with British chalklands, found widely across southeast Britain, and the mobility profile from its unworn M2 is strongly suggestive of repeated movements throughout tooth growth (9 months–3 years) between the chalklands and surrounding territories, potentially including visits to London.

Data S2: Enamel strontium isotope profiles of individual horses

$^{87}Sr/^{86}Sr$ profiles for individual Elverton horses. The red lines indicate the 10-pt mean running average of individual laser ablation measurements (not shown). Measurement uncertainty is estimated as the standard error, shown on the dataplots as dark grey shading around the red lines; in many places, the standard error is less than the width of the red line and is therefore not visible. The 'local' London $^{87}Sr/^{86}Sr$ baseline is indicated by light grey shading. Black bars indicate the part of the profile selected for enamel carbonate analysis ($\delta^{18}O$ and $\delta^{13}C$); carbonate samples were collected from enamel immediately adjacent to that analysed for Sr isotope analysis wherever possible.

Horses with mobility within the range >0.714

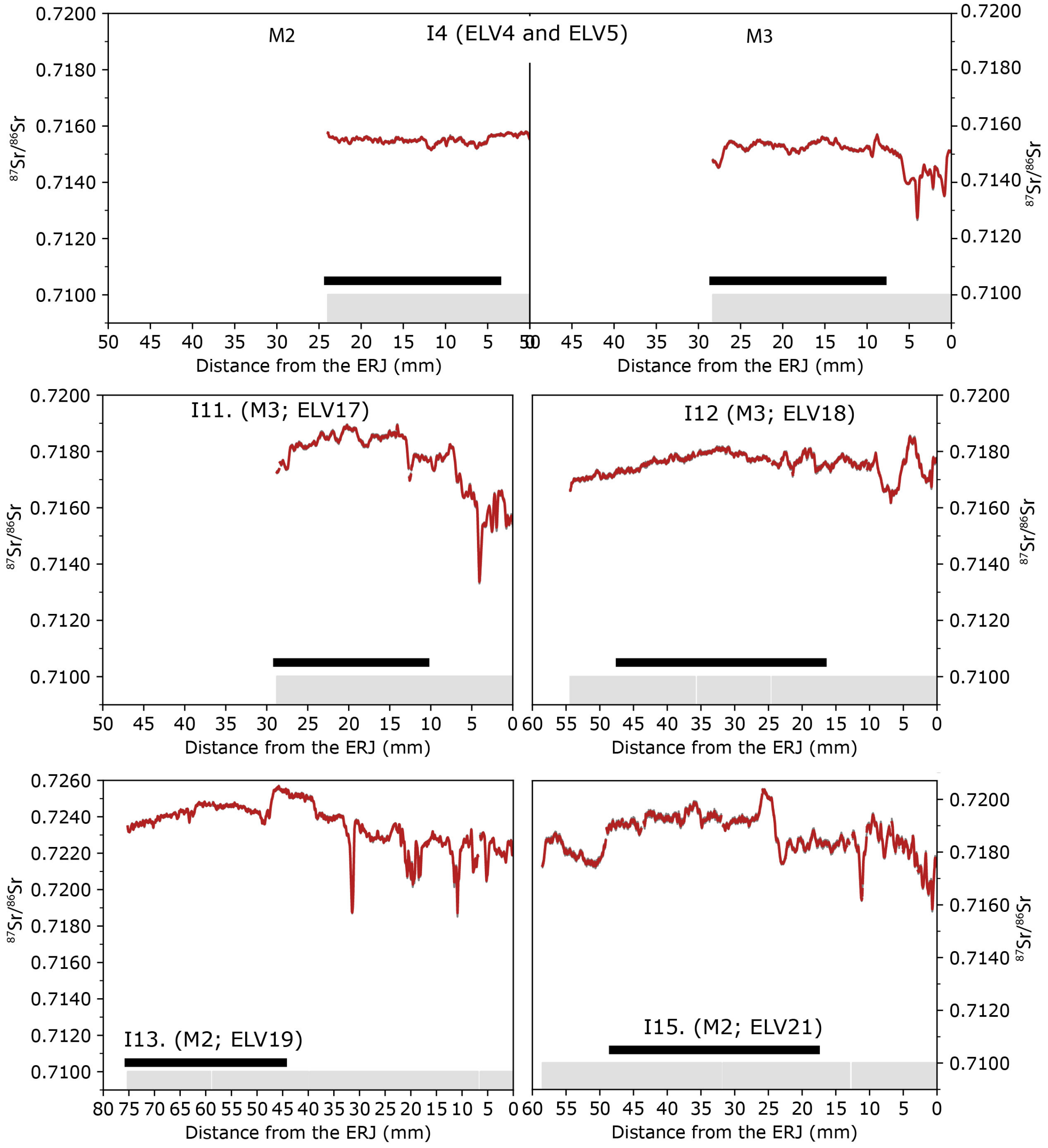

Horse M2-M3 pairs with mobility within the range 0.708-0.713

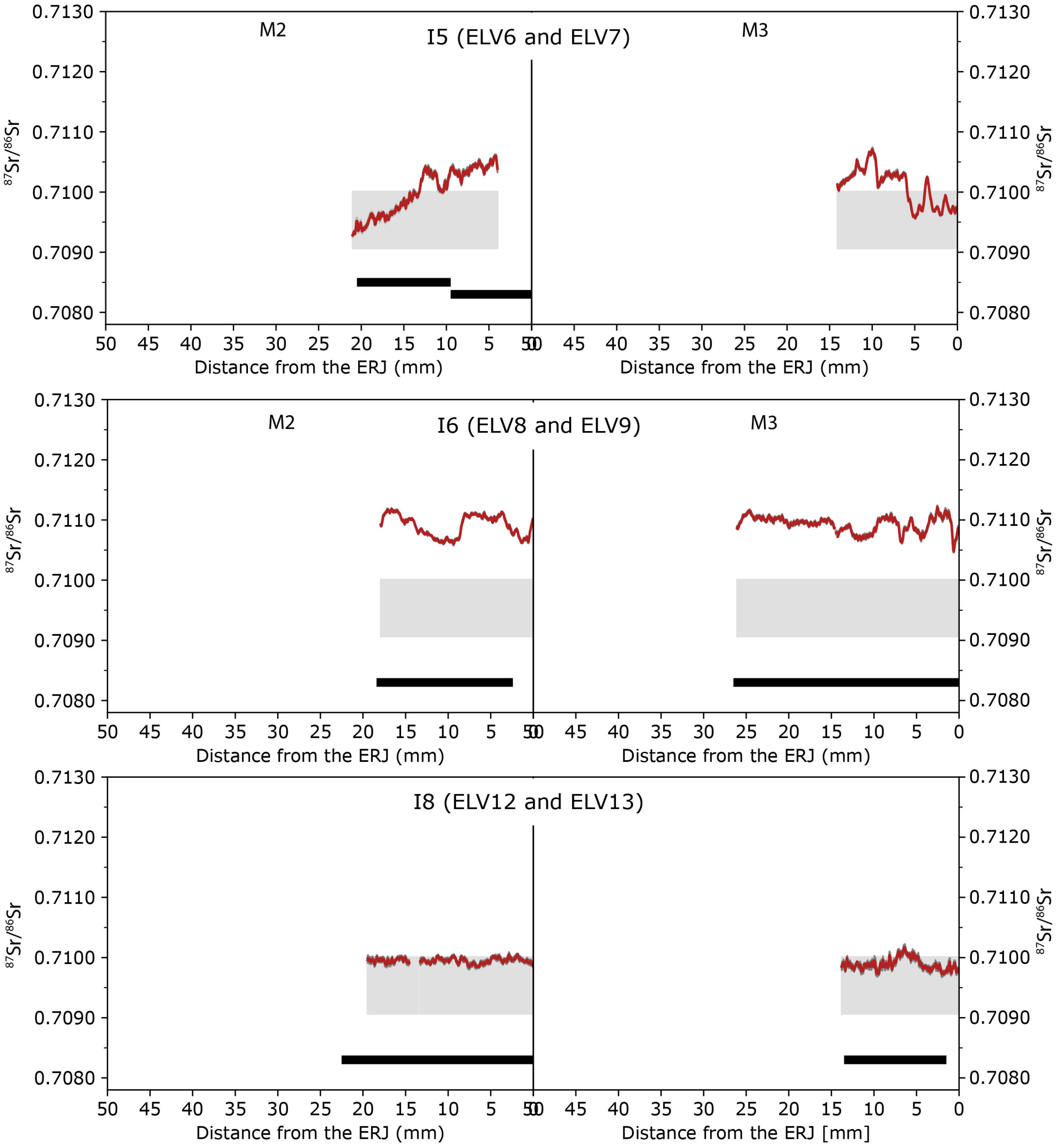

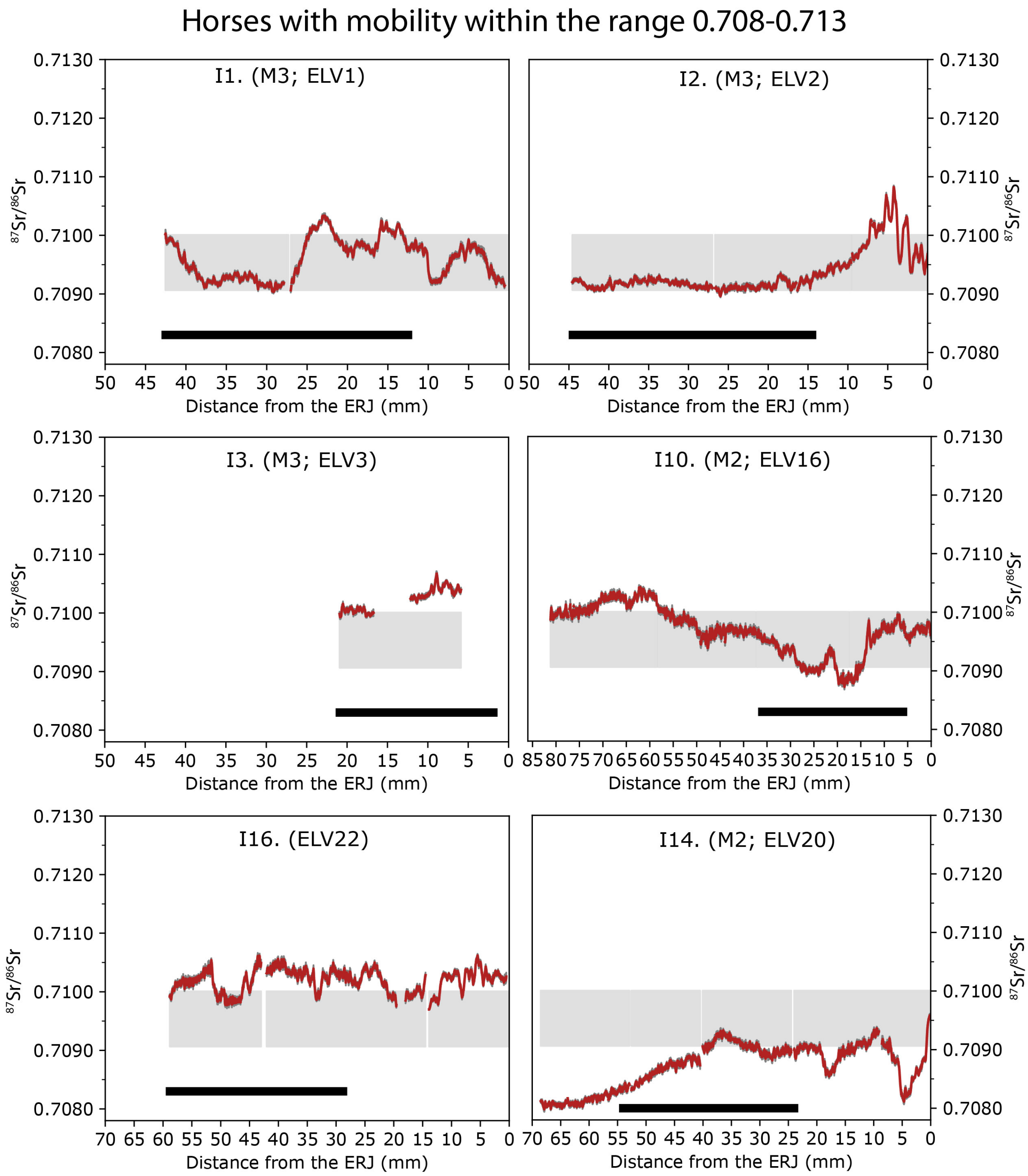

Horses with mobility within the range 0.708-0.713
I1. (M3; ELV1)
I2. (M3; ELV2)
I3. (M3; ELV3)
I10. (M2; ELV16)
I16. (ELV22)
I14. (M2; ELV20)
$^{87}Sr/^{86}Sr$
Distance from the ERJ (mm)
0.7130
0.7120
0.7110
0.7100
0.7090
0.7080

Similar mobility profiles of I7 M2-M3 tooth pairs

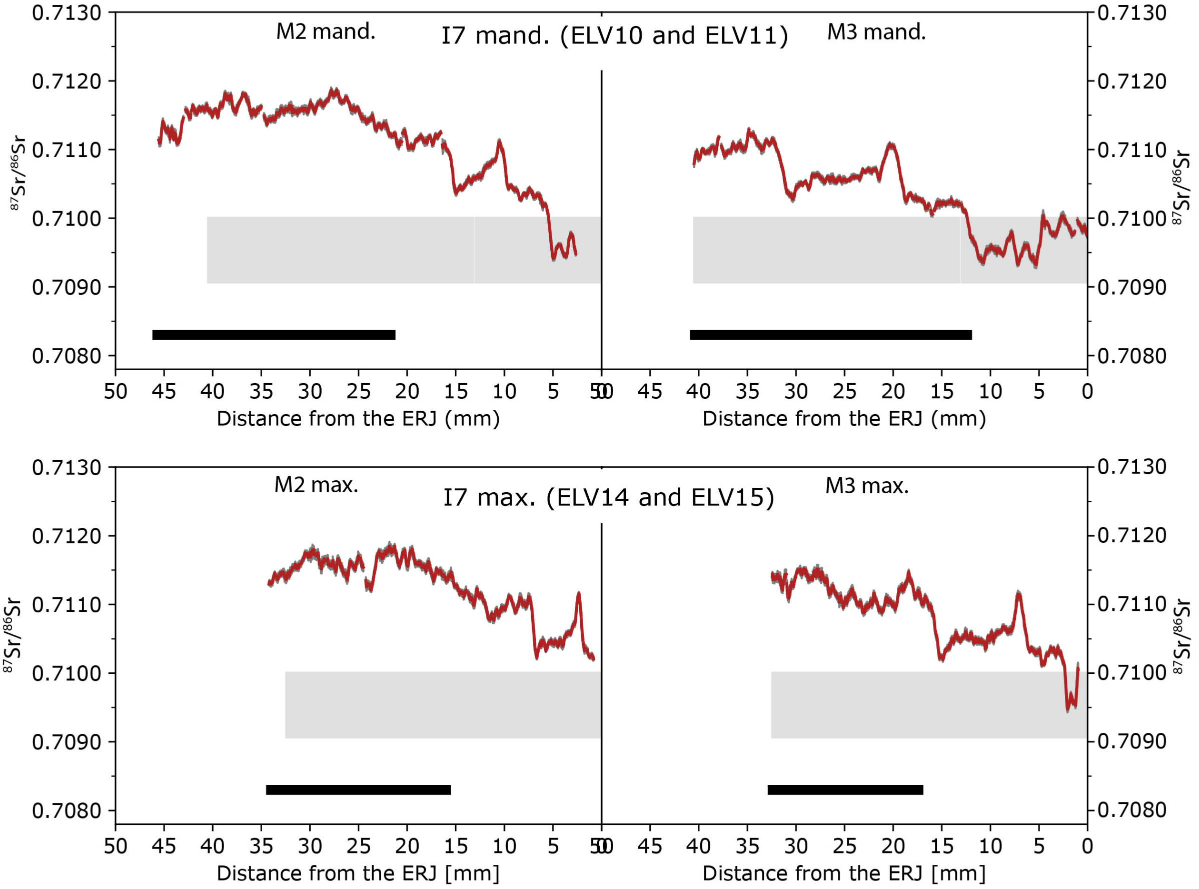

Data S3: Graphs showing output from laser ablation analyses per tooth (^{88}Sr, $^{87}Sr/^{86}Sr$, $^{84}Sr/^{86}Sr$, $^{85}Rb/^{86}Sr$, ^{89}Y)

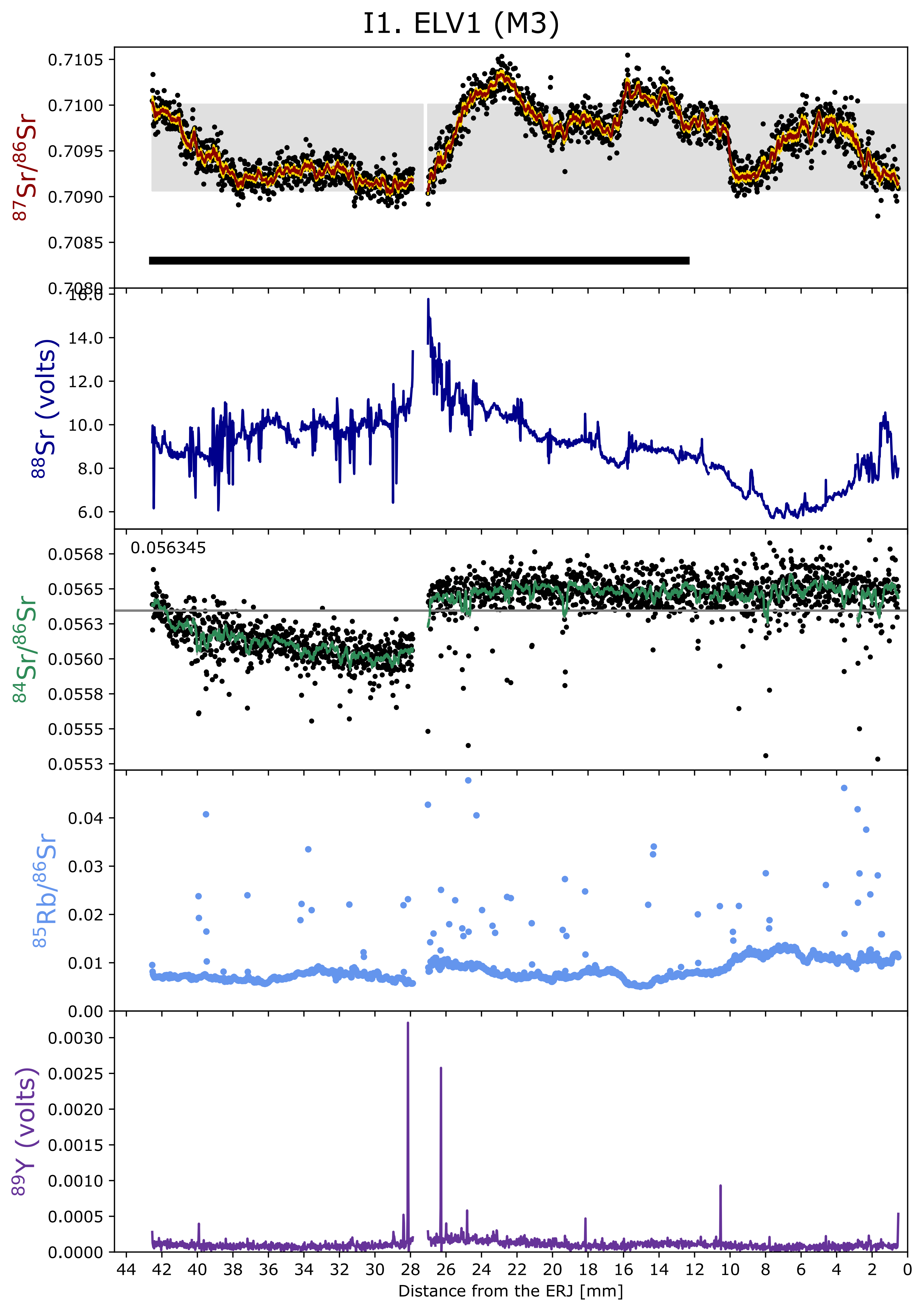

I1. ELV1 (M3)
^{87}Sr/^{86}Sr
0.7105
0.7100
0.7095
0.7090
0.7085
0.7080
^{88}Sr (volts)
16.0
14.0
12.0
10.0
8.0
6.0
0.056345
^{84}Sr/^{86}Sr
0.5568
0.0565
0.0563
0.0560
0.0558
0.0555
0.0553
^{85}Rb/^{86}Sr
0.04
0.03
0.02
0.01
0.00
^{89}Y (volts)
0.0030
0.0025
0.0020
0.0015
0.0010
0.0005
0.0000
44 42 40 38 36 34 32 30 28 26 24 22 20 18 16 14 12 10 8 6 4 2 0
Distance from the ERJ [mm]

I2. ELV2 (M3)

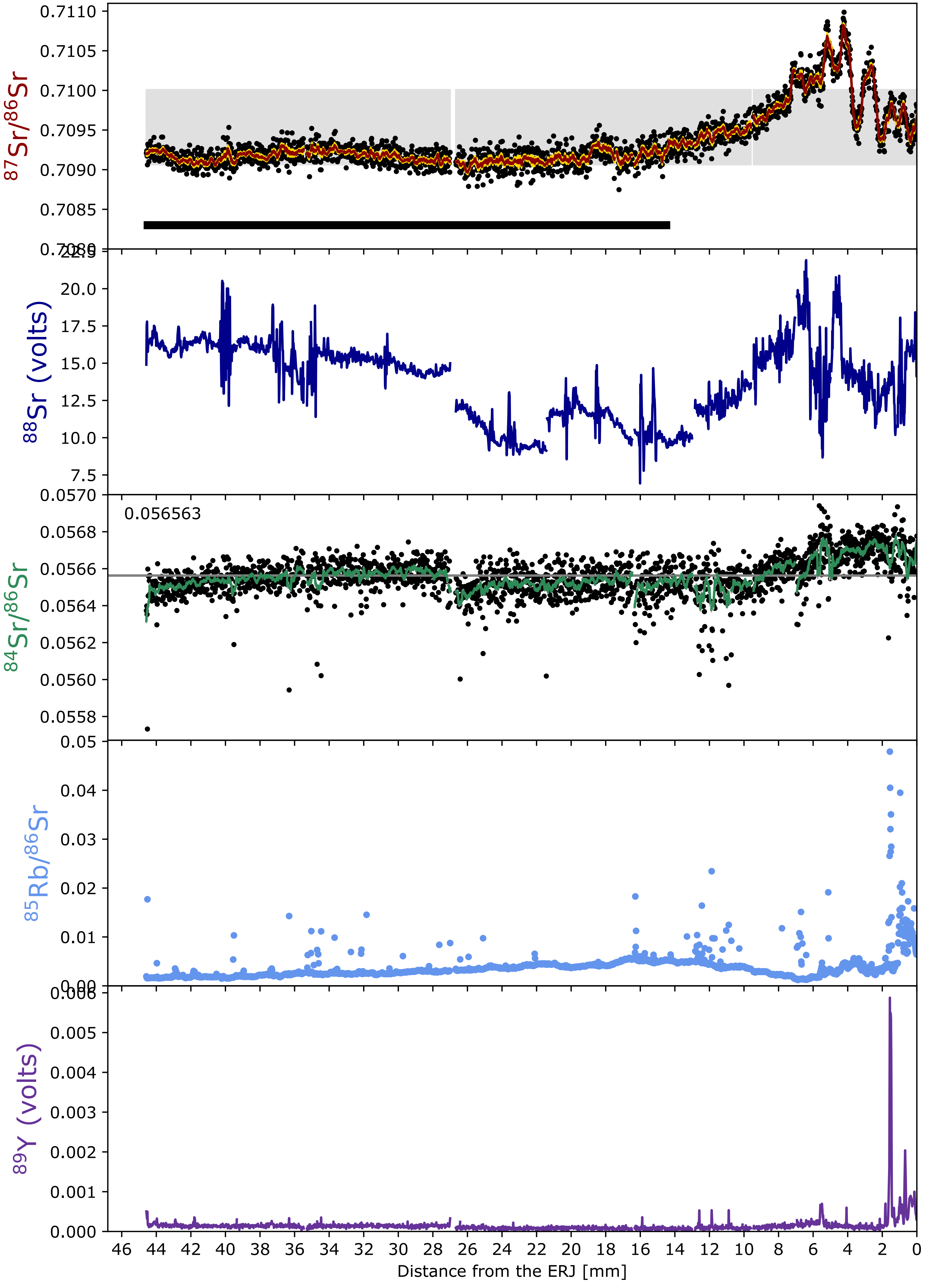

$^{87}Sr/^{86}Sr$
0.7110
0.7105
0.7100
0.7095
0.7090
0.7085
0.7080
^{88}Sr (volts)
22.5
20.0
17.5
15.0
12.5
10.0
7.5
$^{84}Sr/^{86}Sr$
0.0570
0.056563
0.0568
0.0566
0.0564
0.0562
0.0560
0.0558
$^{85}Rb/^{86}Sr$
0.05
0.04
0.03
0.02
0.01
0.00
^{89}Y (volts)
0.006
0.005
0.004
0.003
0.002
0.001
0.000
46 44 42 40 38 36 34 32 30 28 26 24 22 20 18 16 14 12 10 8 6 4 2 0
Distance from the ERJ [mm]

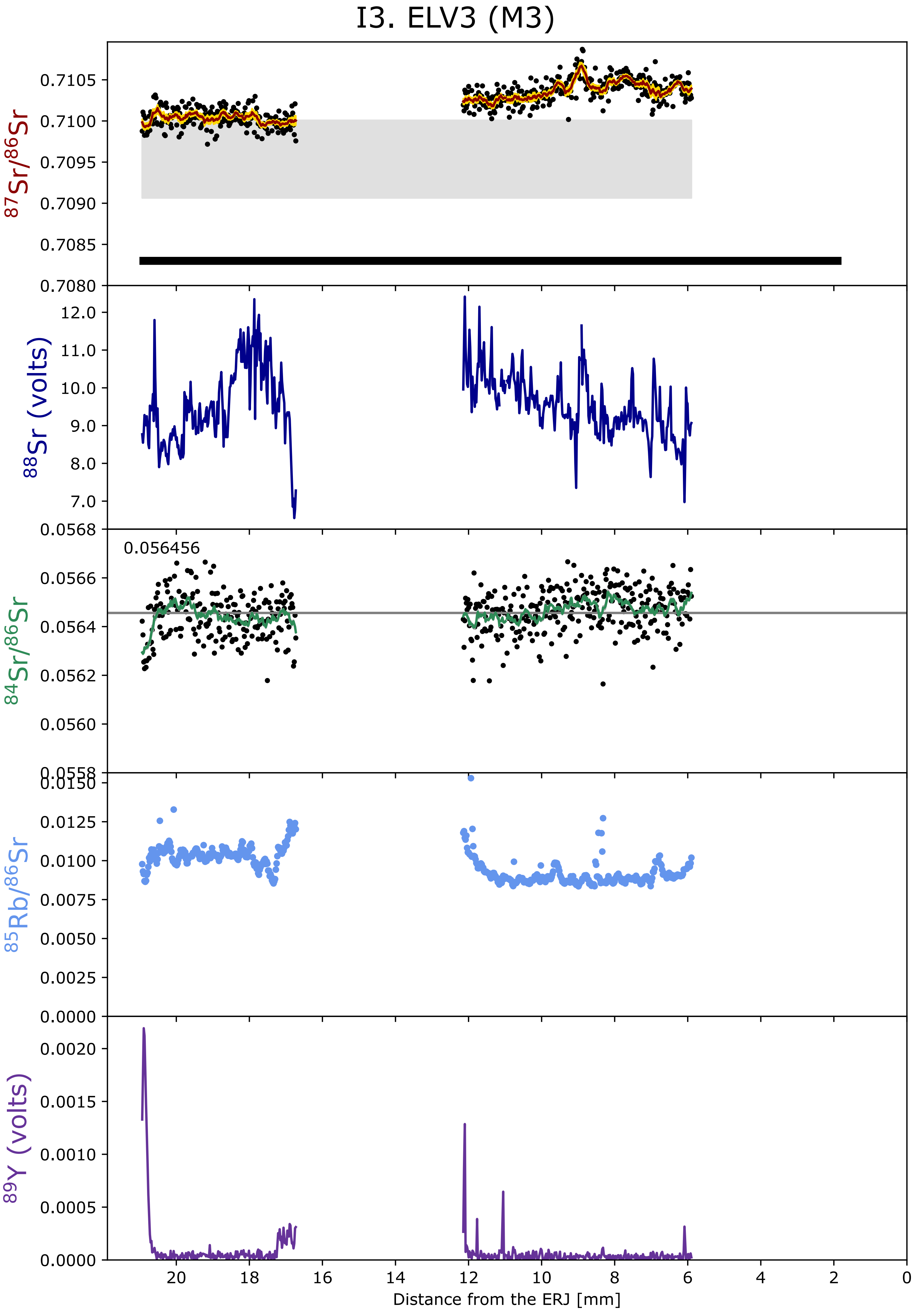

I3. ELV3 (M3)
^{87}Sr/^{86}Sr
0.7105
0.7100
0.7095
0.7090
0.7085
0.7080
^{88}Sr (volts)
12.0
11.0
10.0
9.0
8.0
7.0
0.0568
0.056456
0.0566
0.0564
0.0562
0.0560
0.0558
^{84}Sr/^{86}Sr
0.0150
0.0125
0.0100
0.0075
0.0050
0.0025
0.0000
^{85}Rb/^{86}Sr
0.0020
0.0015
0.0010
0.0005
0.0000
^{89}Y (volts)
20
18
16
14
12
10
8
6
4
2
0
Distance from the ERJ [mm]

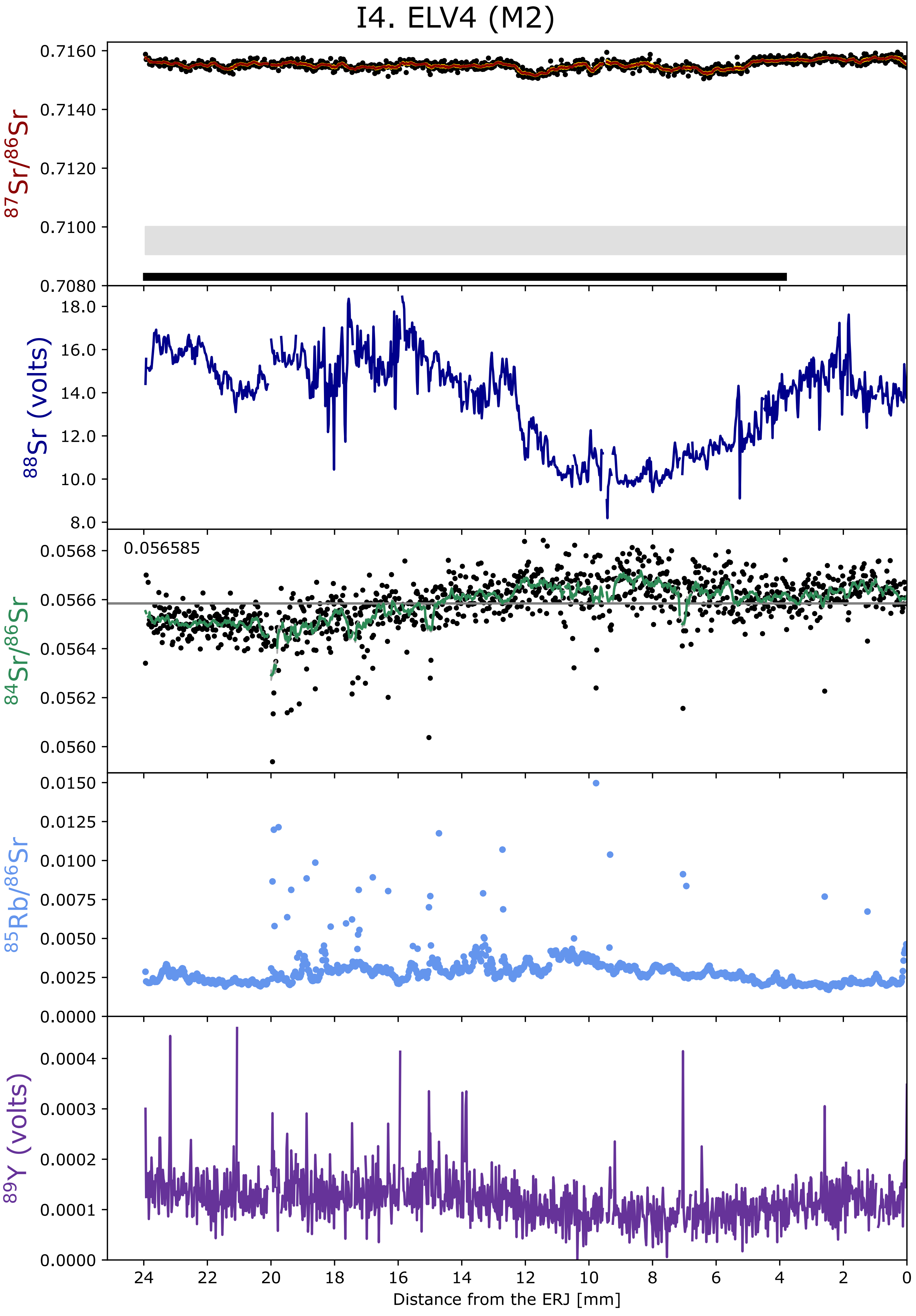

I4. ELV4 (M2)
$^{87}Sr/^{86}Sr$
0.7160
0.7140
0.7120
0.7100
0.7080
^{88}Sr (volts)
18.0
16.0
14.0
12.0
10.0
8.0
$^{84}Sr/^{86}Sr$
0.056585
0.0568
0.0566
0.0564
0.0562
0.0560
$^{85}Rb/^{86}Sr$
0.0150
0.0125
0.0100
0.0075
0.0050
0.0025
0.0000
^{89}Y (volts)
0.0004
0.0003
0.0002
0.0001
0.0000
24
22
20
18
16
14
12
10
8
6
4
2
0
Distance from the ERJ [mm]

I4. ELV5 (M3)

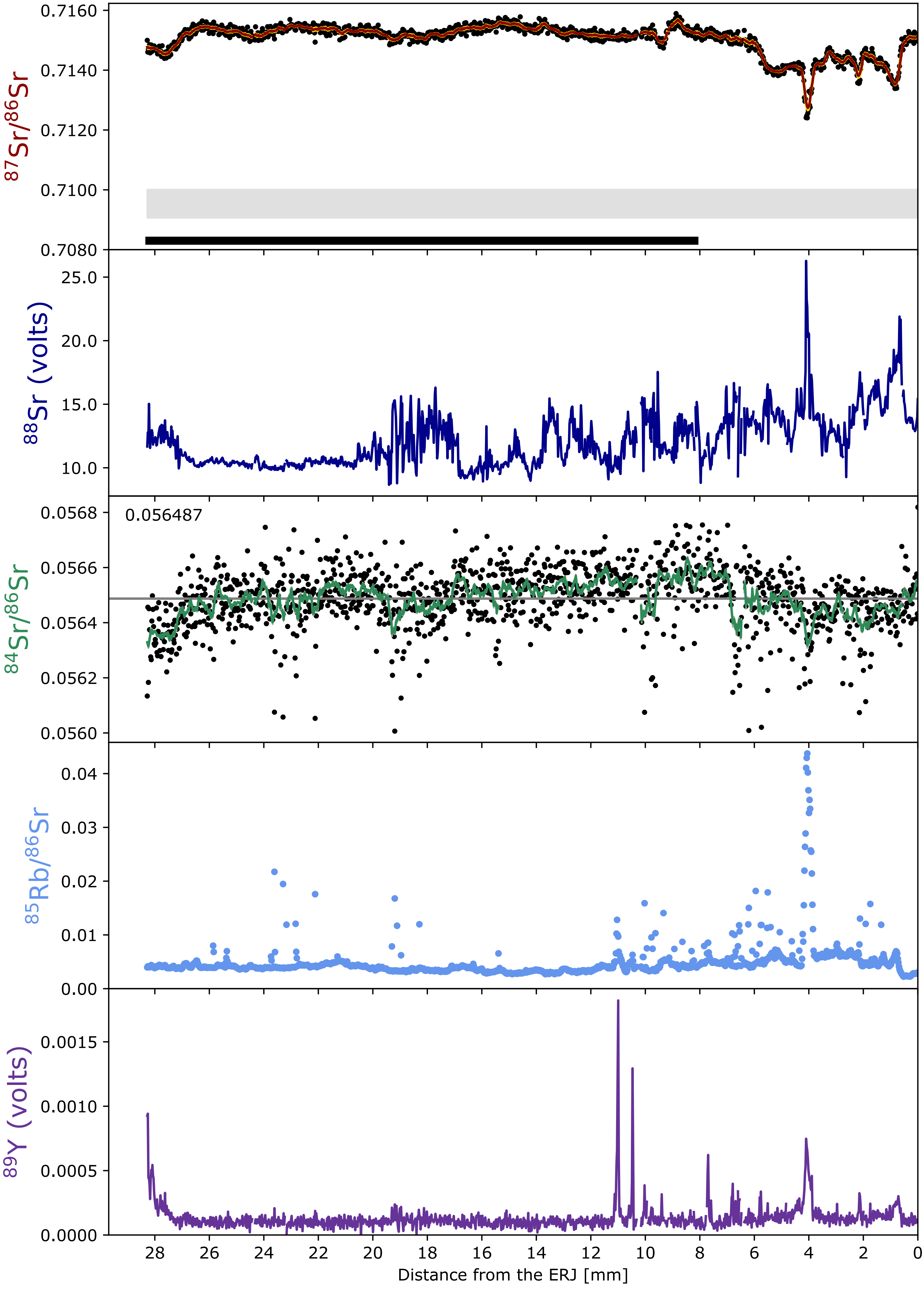

0.7160
0.7140
0.7120
0.7100
0.7080
$^{87}Sr/^{86}Sr$
25.0
20.0
15.0
10.0
^{88}Sr (volts)
0.0568
0.0566
0.0564
0.0562
0.0560
0.056487
$^{84}Sr/^{86}Sr$
0.04
0.03
0.02
0.01
0.00
$^{85}Rb/^{86}Sr$
0.0015
0.0010
0.0005
0.0000
^{89}Y (volts)
28
26
24
22
20
18
16
14
12
10
8
6
4
2
0
Distance from the ERJ [mm]

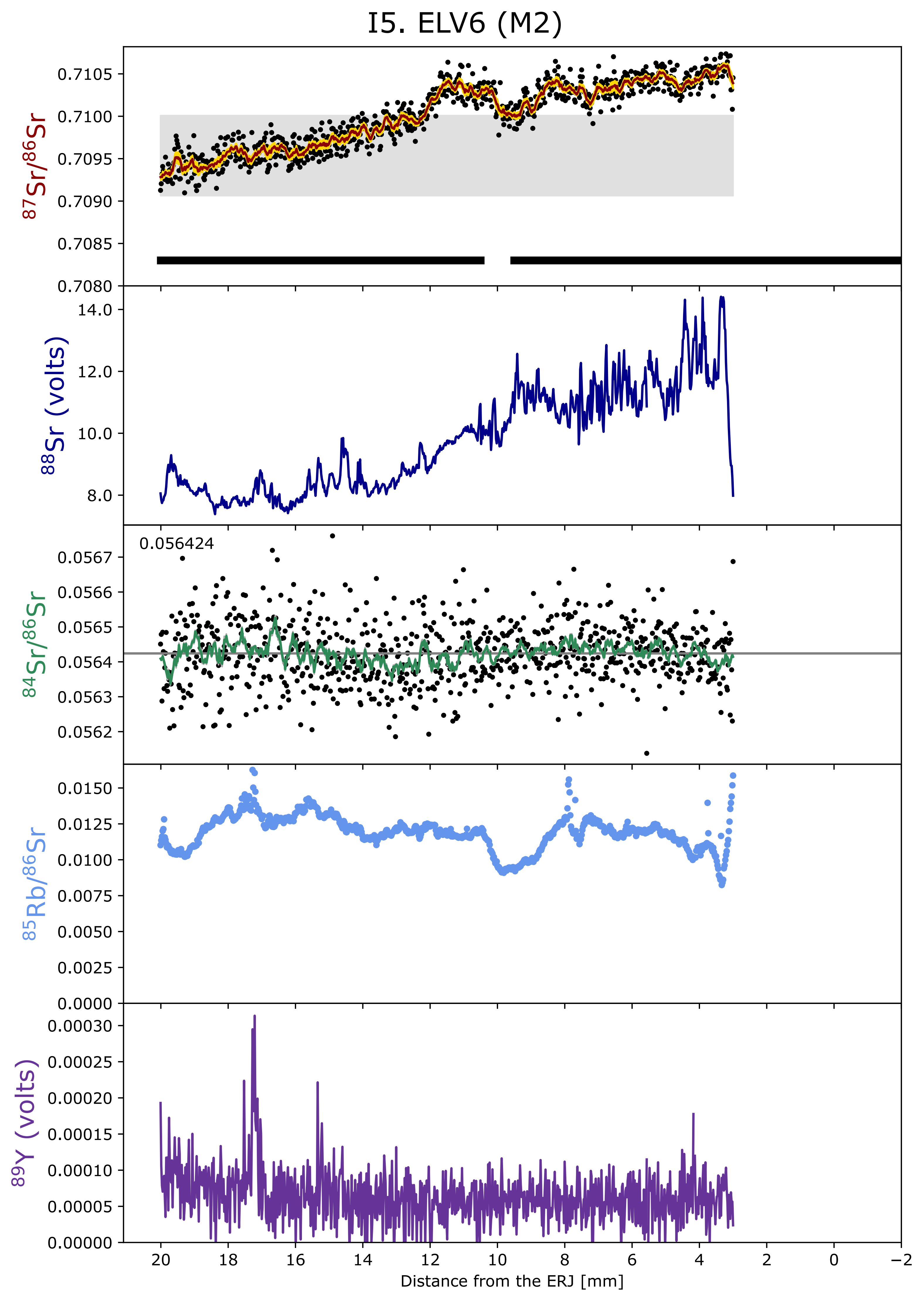

I5. ELV6 (M2)
$^{87}Sr/^{86}Sr$
^{88}Sr (volts)
$^{84}Sr/^{86}Sr$
0.056424
$^{85}Rb/^{86}Sr$
^{89}Y (volts)
Distance from the ERJ [mm]

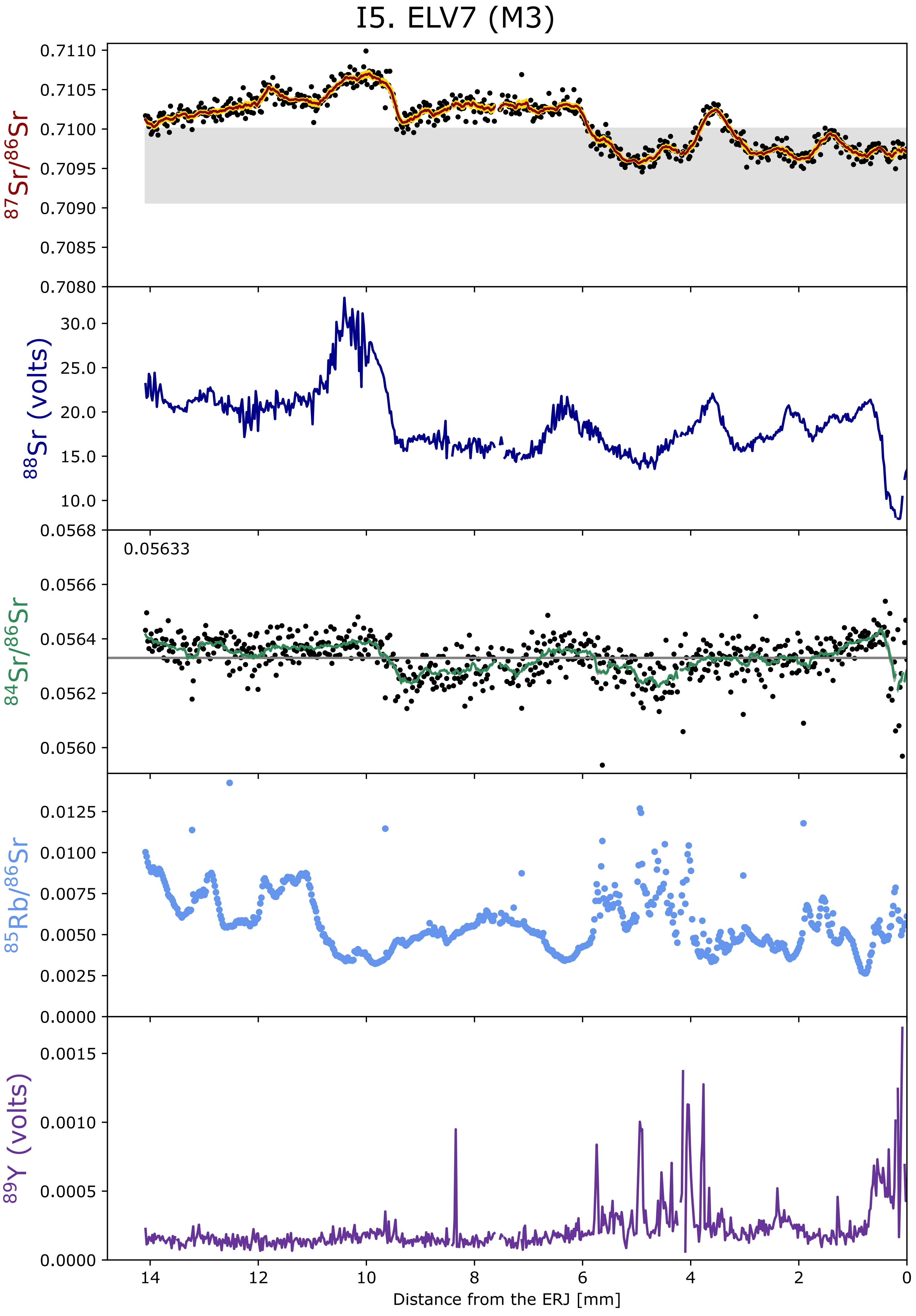
I5. ELV7 (M3)
$^{87}Sr/^{86}Sr$
0.7110
0.7105
0.7100
0.7095
0.7090
0.7085
0.7080
^{88}Sr (volts)
30.0
25.0
20.0
15.0
10.0
0.0568
0.05633
$^{84}Sr/^{86}Sr$
0.0566
0.0564
0.0562
0.0560
$^{85}Rb/^{86}Sr$
0.0125
0.0100
0.0075
0.0050
0.0025
0.0000
^{89}Y (volts)
0.0015
0.0010
0.0005
0.0000
14
12
10
8
6
4
2
0
Distance from the ERJ [mm]

I6. ELV8 (M2)

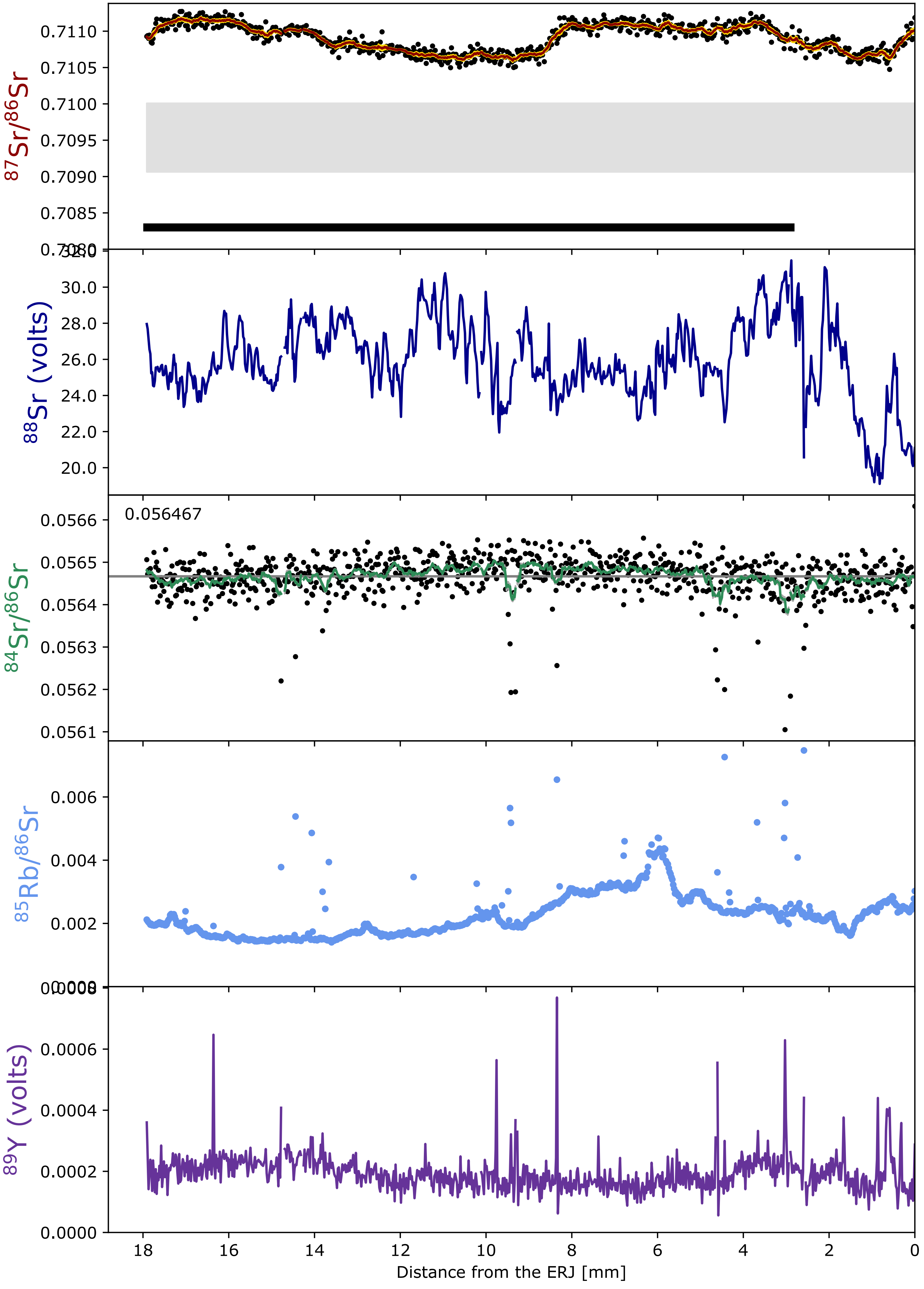

0.7110
0.7105
0.7100
0.7095
0.7090
0.7085
0.7080
$^{87}Sr/^{86}Sr$
32.0
30.0
28.0
26.0
24.0
22.0
20.0
^{88}Sr (volts)
0.056467
0.0566
0.0565
0.0564
0.0563
0.0562
0.0561
$^{84}Sr/^{86}Sr$
0.006
0.004
0.002
0.000
$^{85}Rb/^{86}Sr$
0.0008
0.0006
0.0004
0.0002
0.0000
^{89}Y (volts)
18
16
14
12
10
8
6
4
2
0
Distance from the ERJ [mm]

I6. ELV9 (M3)

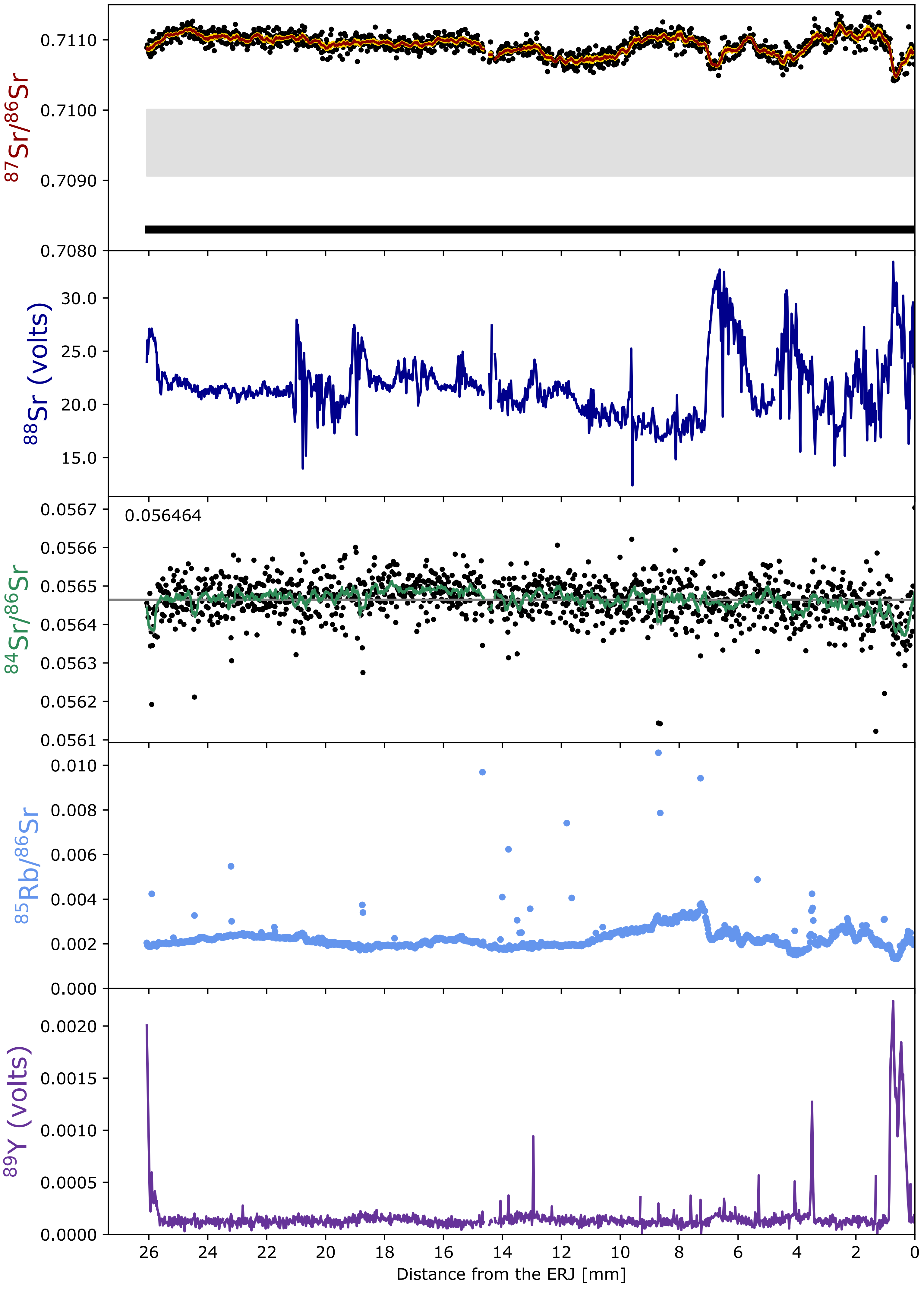

$^{87}Sr/^{86}Sr$
0.7110
0.7100
0.7090
0.7080
^{88}Sr (volts)
30.0
25.0
20.0
15.0
$^{84}Sr/^{86}Sr$
0.056464
0.0567
0.0566
0.0565
0.0564
0.0563
0.0562
0.0561
$^{85}Rb/^{86}Sr$
0.010
0.008
0.006
0.004
0.002
0.000
^{89}Y (volts)
0.0020
0.0015
0.0010
0.0005
0.0000
26
24
22
20
18
16
14
12
10
8
6
4
2
0
Distance from the ERJ [mm]

I7. ELV10 (M2)

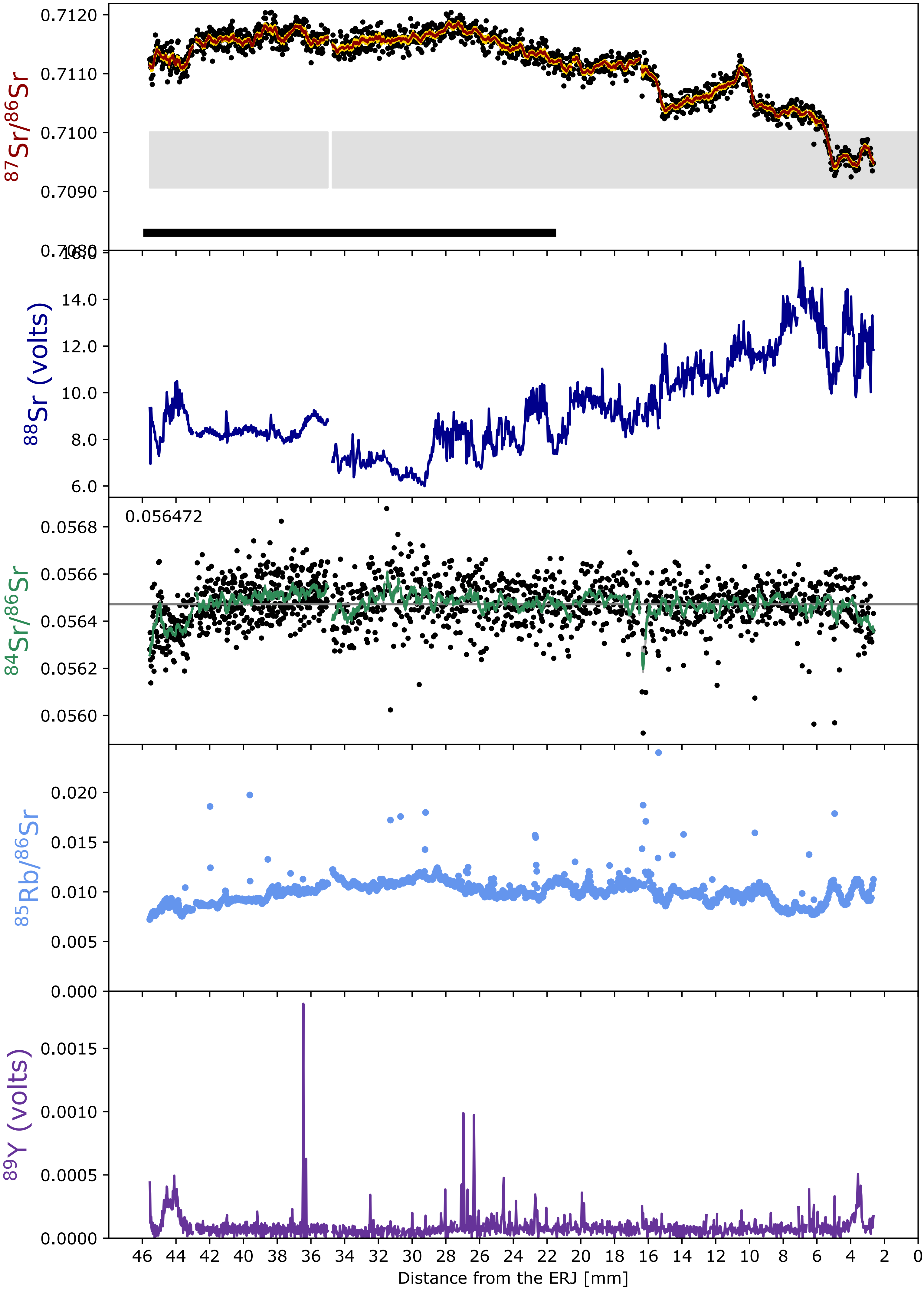

$^{87}Sr/^{86}Sr$
0.7120
0.7110
0.7100
0.7090
0.7080
^{88}Sr (volts)
16.0
14.0
12.0
10.0
8.0
6.0
$^{84}Sr/^{86}Sr$
0.056472
0.0568
0.0566
0.0564
0.0562
0.0560
$^{85}Rb/^{86}Sr$
0.020
0.015
0.010
0.005
0.000
^{89}Y (volts)
0.0015
0.0010
0.0005
0.0000
46 44 42 40 38 36 34 32 30 28 26 24 22 20 18 16 14 12 10 8 6 4 2 0
Distance from the ERJ [mm]

I7. ELV11 (M3)

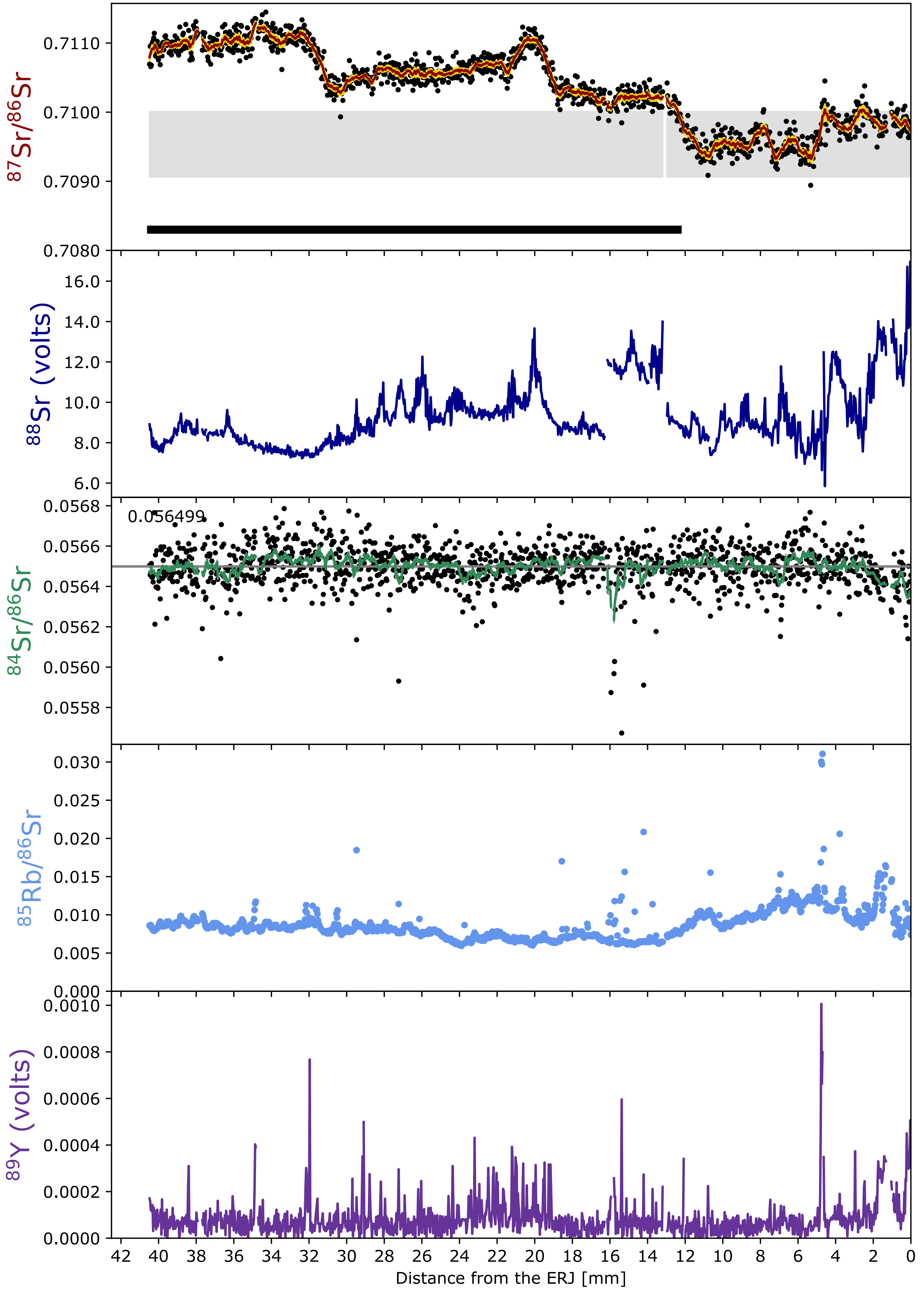

$^{87}Sr/^{86}Sr$
0.7110
0.7100
0.7090
0.7080
^{88}Sr (volts)
16.0
14.0
12.0
10.0
8.0
6.0
$^{84}Sr/^{86}Sr$
0.0568
0.0566
0.0564
0.0562
0.0560
0.0558
0.056499
$^{85}Rb/^{86}Sr$
0.030
0.025
0.020
0.015
0.010
0.005
0.000
^{89}Y (volts)
0.0010
0.0008
0.0006
0.0004
0.0002
0.0000
42 40 38 36 34 32 30 28 26 24 22 20 18 16 14 12 10 8 6 4 2 0
Distance from the ERJ [mm]

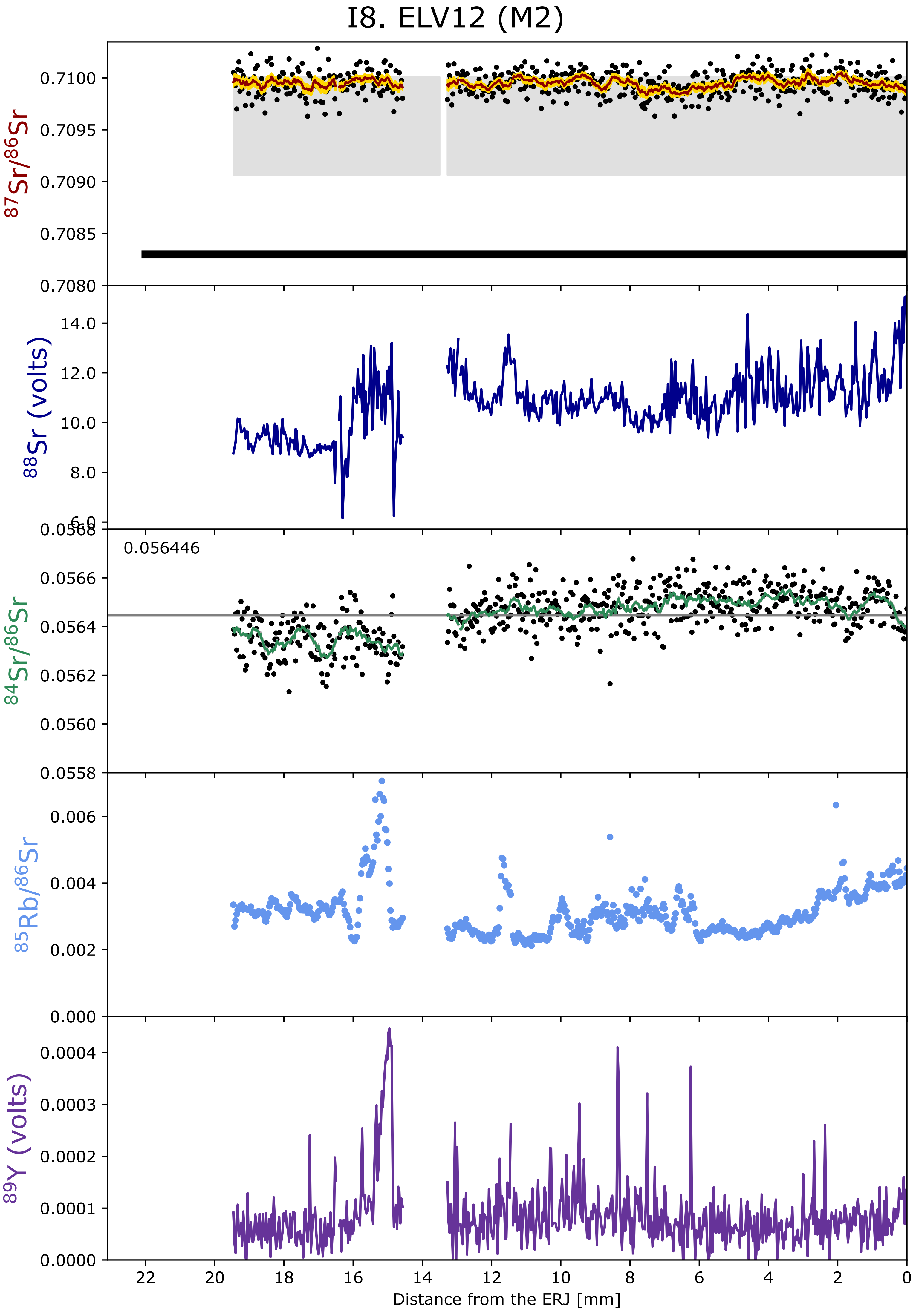
I8. ELV12 (M2)
$^{87}Sr/^{86}Sr$
^{88}Sr (volts)
$^{84}Sr/^{86}Sr$
0.056446
$^{85}Rb/^{86}Sr$
^{89}Y (volts)
Distance from the ERJ [mm]

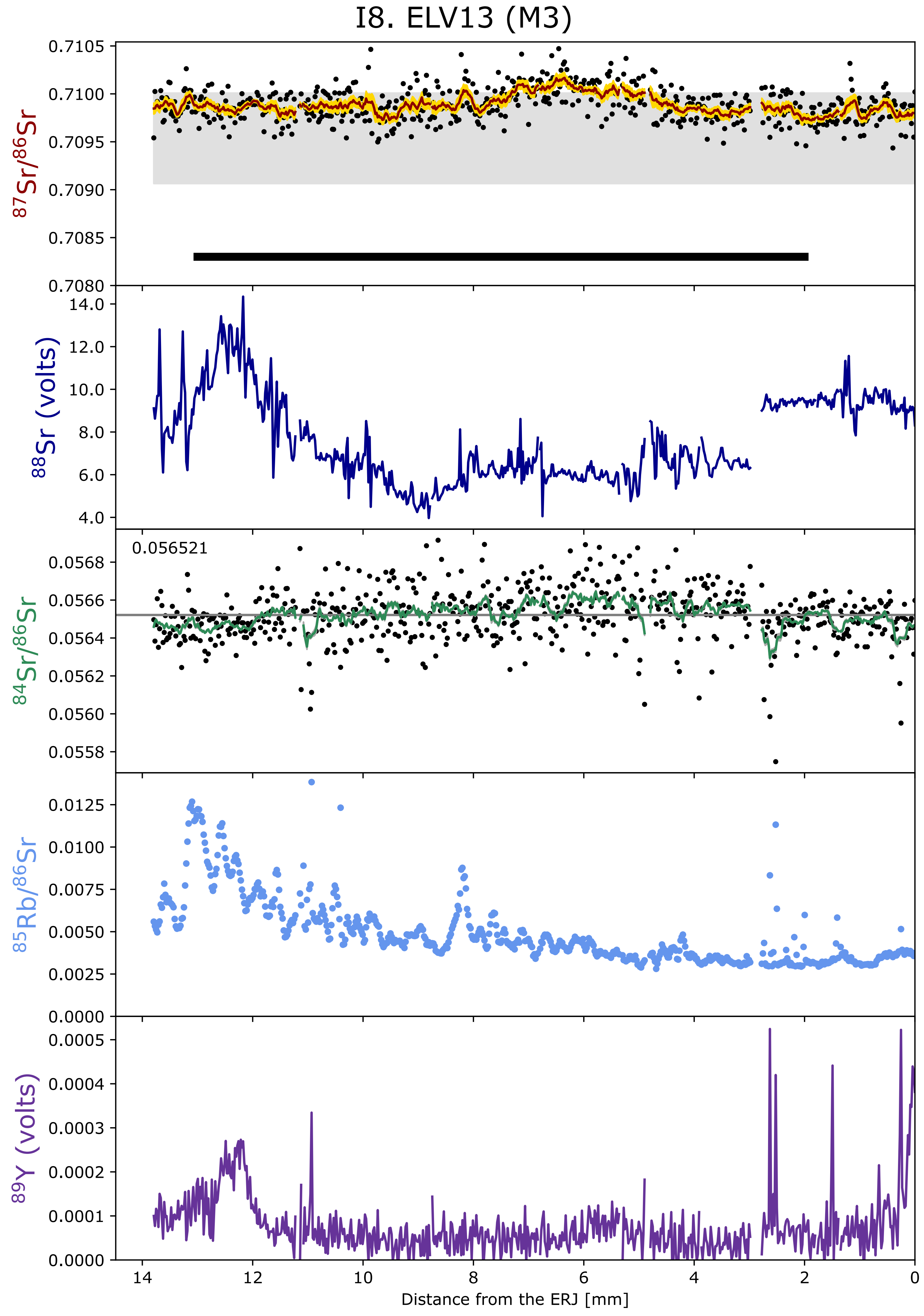
I8. ELV13 (M3)
^{87}Sr/^{86}Sr
0.7105
0.7100
0.7095
0.7090
0.7085
0.7080
^{88}Sr (volts)
14.0
12.0
10.0
8.0
6.0
4.0
0.056521
^{84}Sr/^{86}Sr
0.5668
0.0566
0.0564
0.0562
0.0560
0.0558
^{85}Rb/^{86}Sr
0.0125
0.0100
0.0075
0.0050
0.0025
0.0000
^{89}Y (volts)
0.0005
0.0004
0.0003
0.0002
0.0001
0.0000
14
12
10
8
6
4
2
0
Distance from the ERJ [mm]

I9. ELV14 (M2)

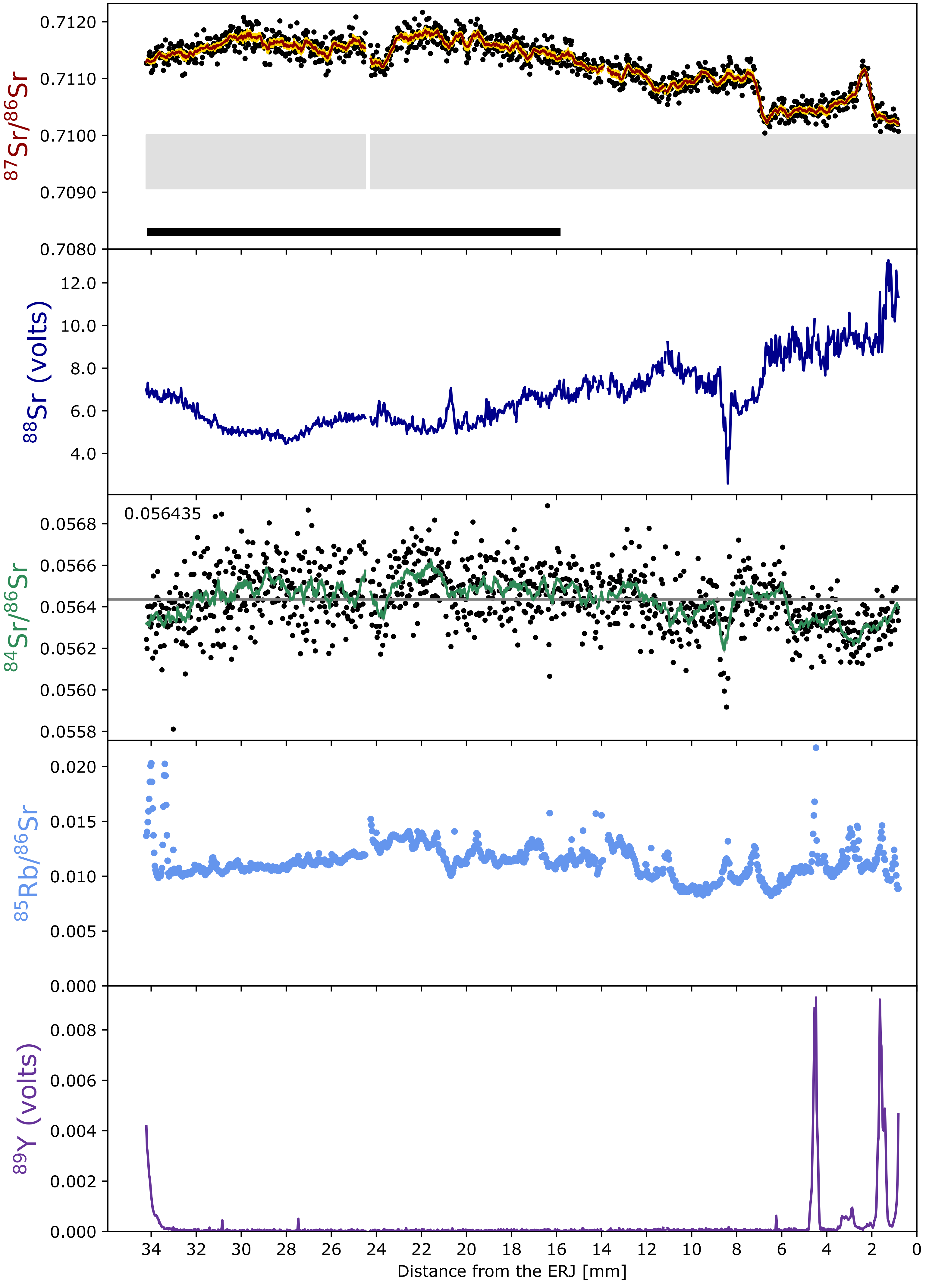

0.7120
0.7110
0.7100
0.7090
0.7080
^{87}Sr/^{86}Sr
12.0
10.0
8.0
6.0
4.0
^{88}Sr (volts)
0.056435
0.5568
0.0566
0.0564
0.0562
0.0560
0.0558
^{84}Sr/^{86}Sr
0.020
0.015
0.010
0.005
0.000
^{85}Rb/^{86}Sr
0.008
0.006
0.004
0.002
0.000
^{89}Y (volts)
34 32 30 28 26 24 22 20 18 16 14 12 10 8 6 4 2 0
Distance from the ERJ [mm]

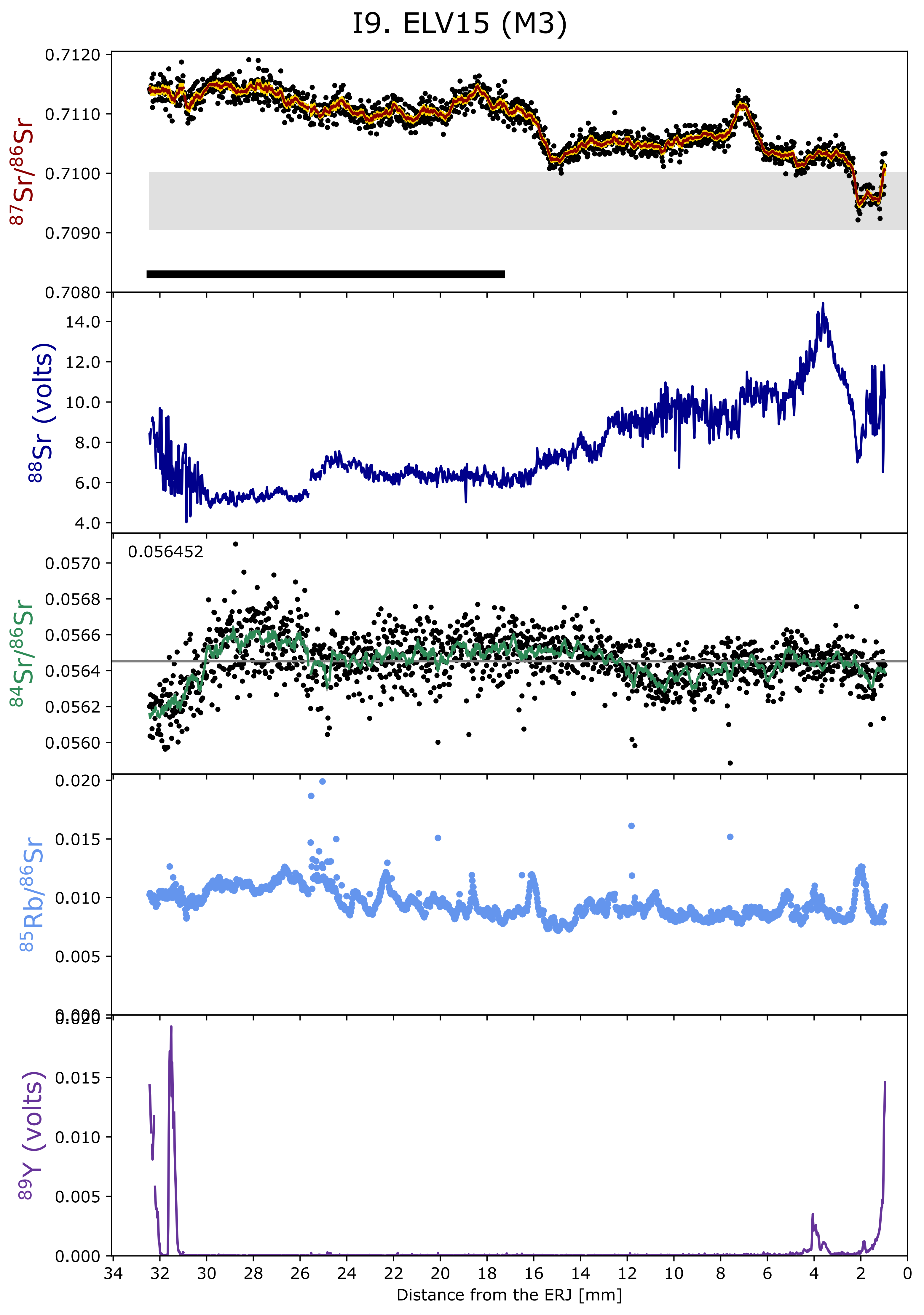
I9. ELV15 (M3)
$^{87}Sr/^{86}Sr$
0.7120
0.7110
0.7100
0.7090
0.7080
^{88}Sr (volts)
14.0
12.0
10.0
8.0
6.0
4.0
$^{84}Sr/^{86}Sr$
0.056452
0.0570
0.0568
0.0566
0.0564
0.0562
0.0560
$^{85}Rb/^{86}Sr$
0.020
0.015
0.010
0.005
0.000
^{89}Y (volts)
0.020
0.015
0.010
0.005
0.000
34
32
30
28
26
24
22
20
18
16
14
12
10
8
6
4
2
0
Distance from the ERJ [mm]

I10. ELV16 (M2)

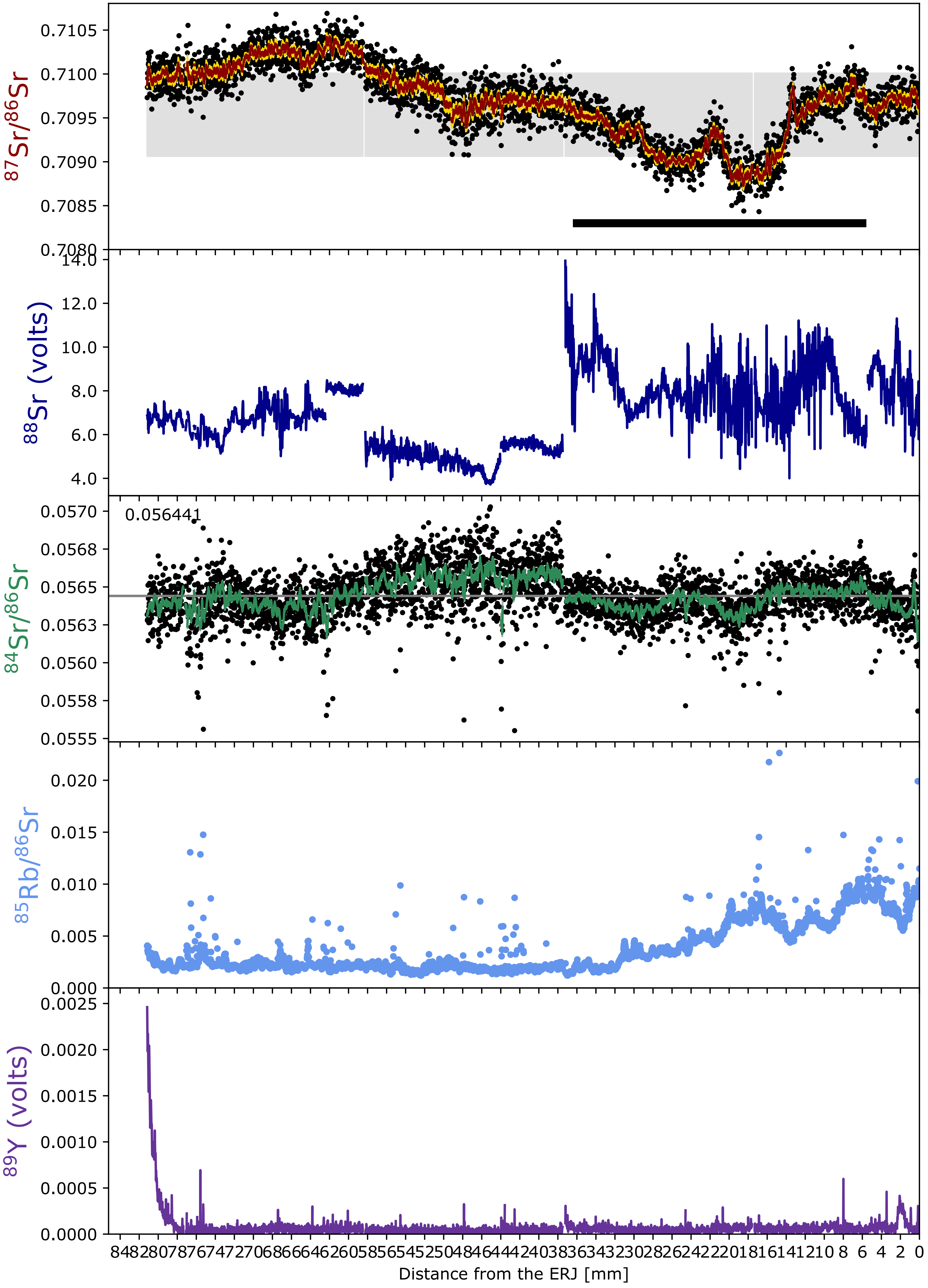

$^{87}Sr/^{86}Sr$
^{88}Sr (volts)
$^{84}Sr/^{86}Sr$
0.056441
$^{85}Rb/^{86}Sr$
^{89}Y (volts)
Distance from the ERJ [mm]

I11. ELV17 (M3)

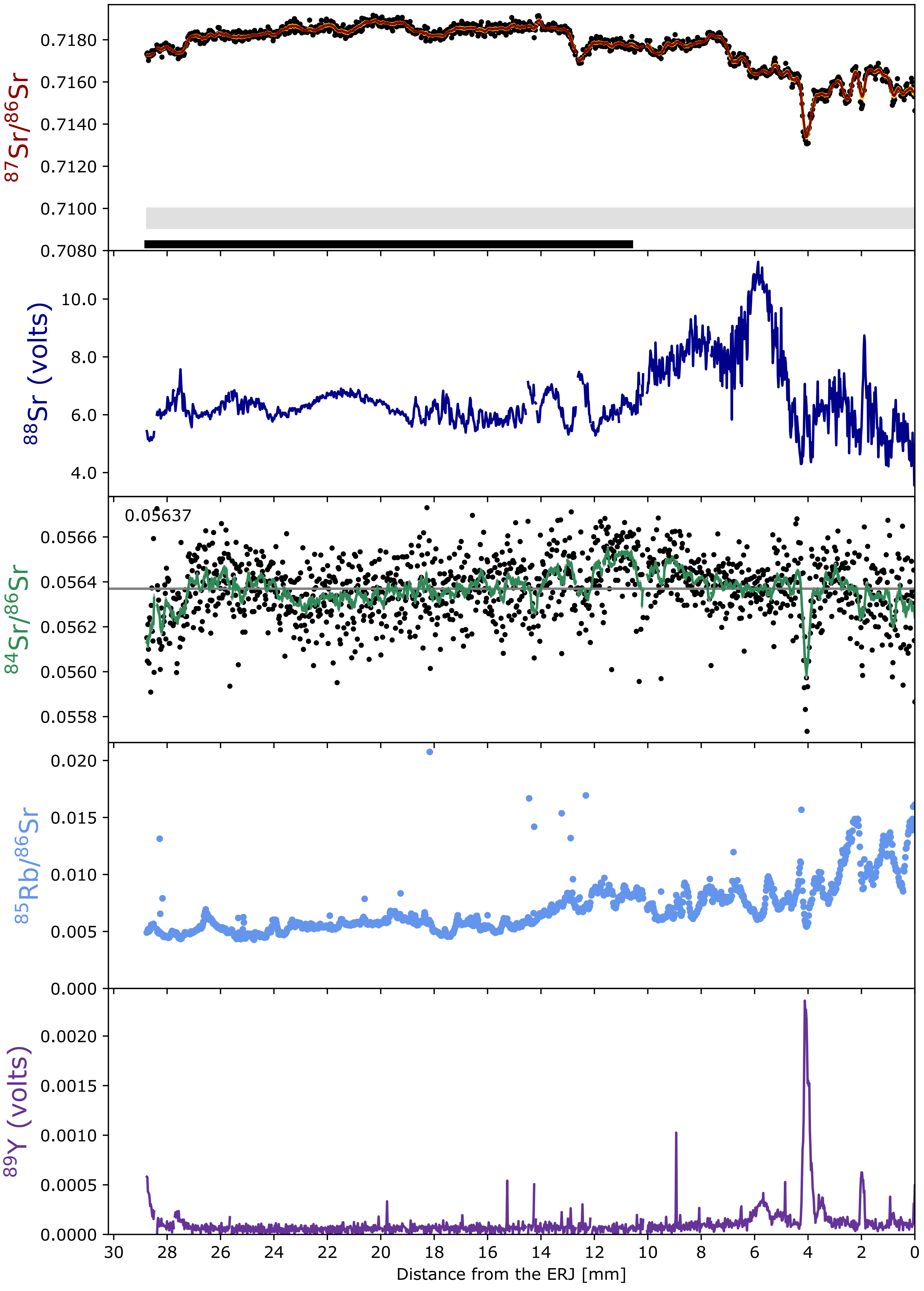

$^{87}Sr/^{86}Sr$
0.7180
0.7160
0.7140
0.7120
0.7100
0.7080
^{88}Sr (volts)
10.0
8.0
6.0
4.0
$^{84}Sr/^{86}Sr$
0.05637
0.0566
0.0564
0.0562
0.0560
0.0558
$^{85}Rb/^{86}Sr$
0.020
0.015
0.010
0.005
0.000
^{89}Y (volts)
0.0020
0.0015
0.0010
0.0005
0.0000
30 28 26 24 22 20 18 16 14 12 10 8 6 4 2 0
Distance from the ERJ [mm]

I12. ELV18 (M3)

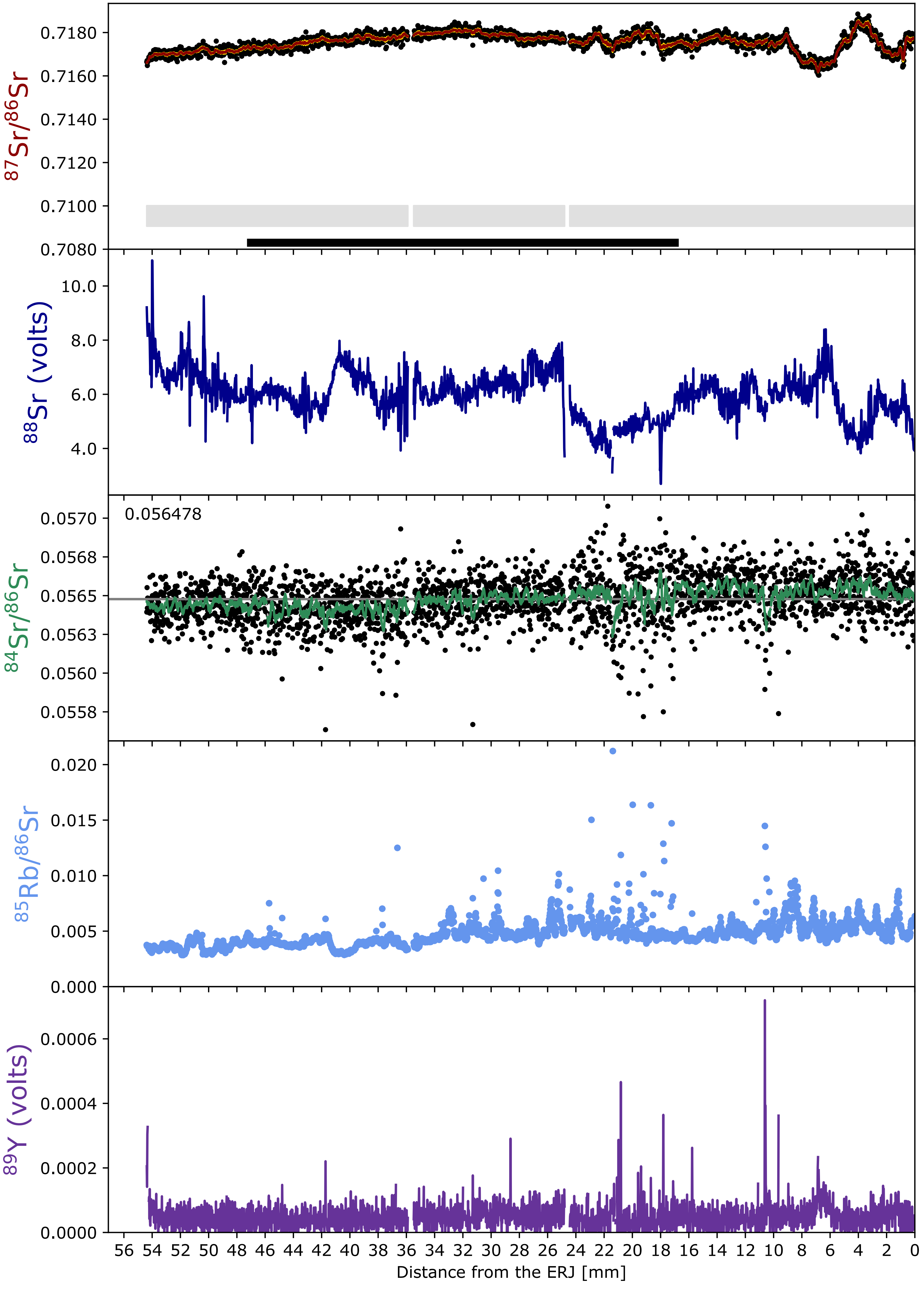

$^{87}Sr/^{86}Sr$
0.7180
0.7160
0.7140
0.7120
0.7100
0.7080
^{88}Sr (volts)
10.0
8.0
6.0
4.0
$^{84}Sr/^{86}Sr$
0.056478
0.0570
0.0568
0.0565
0.0563
0.0560
0.0558
$^{85}Rb/^{86}Sr$
0.020
0.015
0.010
0.005
0.000
^{89}Y (volts)
0.0006
0.0004
0.0002
0.0000
56 54 52 50 48 46 44 42 40 38 36 34 32 30 28 26 24 22 20 18 16 14 12 10 8 6 4 2 0
Distance from the ERJ [mm]

I13. ELV19 (M2)

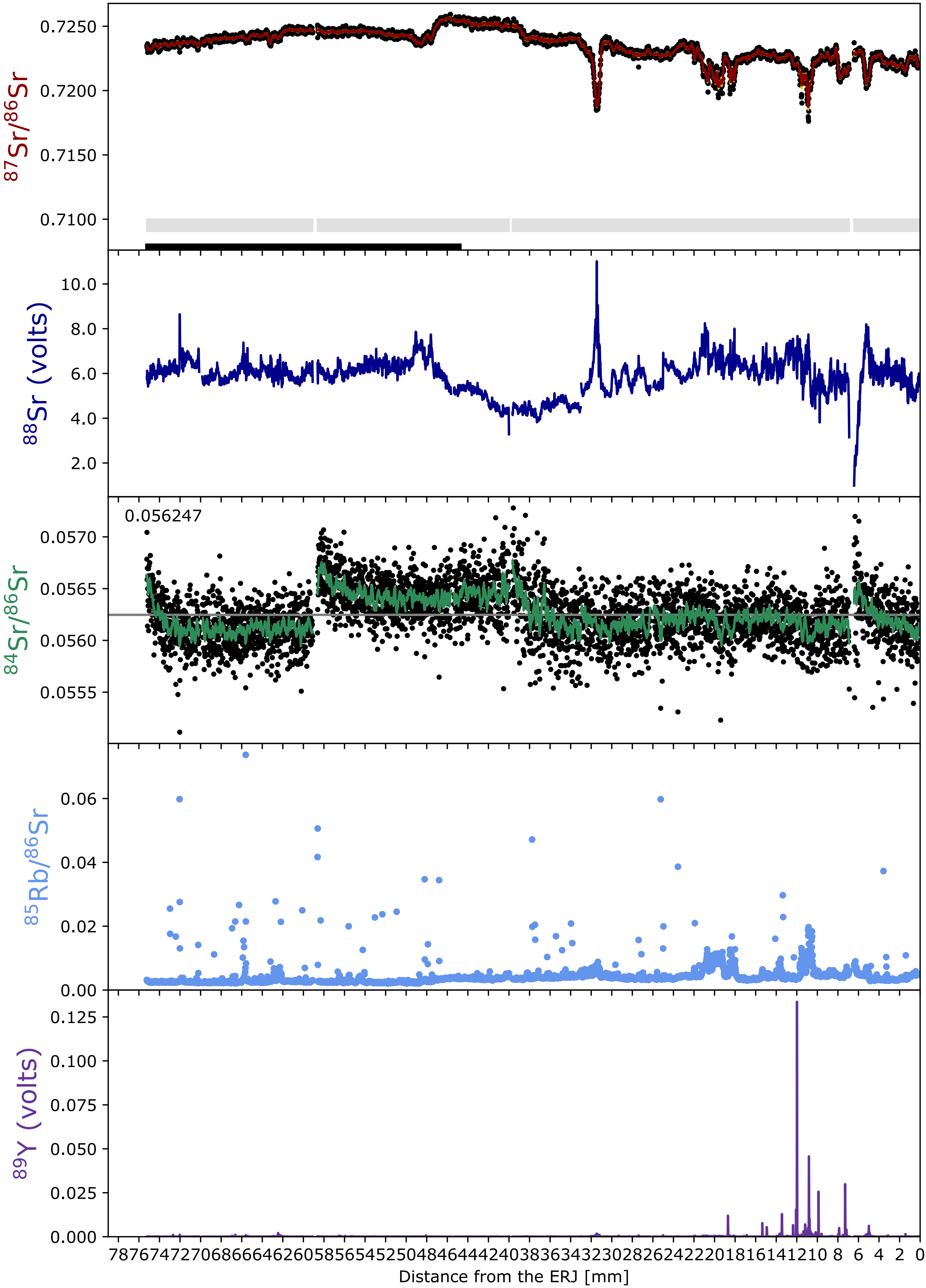

^{87}Sr/^{86}Sr
0.7250
0.7200
0.7150
0.7100
^{88}Sr (volts)
10.0
8.0
6.0
4.0
2.0
^{84}Sr/^{86}Sr
0.056247
0.0570
0.0565
0.0560
0.0555
^{85}Rb/^{86}Sr
0.06
0.04
0.02
0.00
^{89}Y (volts)
0.125
0.100
0.075
0.050
0.025
0.000
78 76 74 72 70 68 66 64 62 60 58 56 54 52 50 48 46 44 42 40 38 36 34 32 30 28 26 24 22 20 18 16 14 12 10 8 6 4 2 0
Distance from the ERJ [mm]

I14. ELV20 (M2)

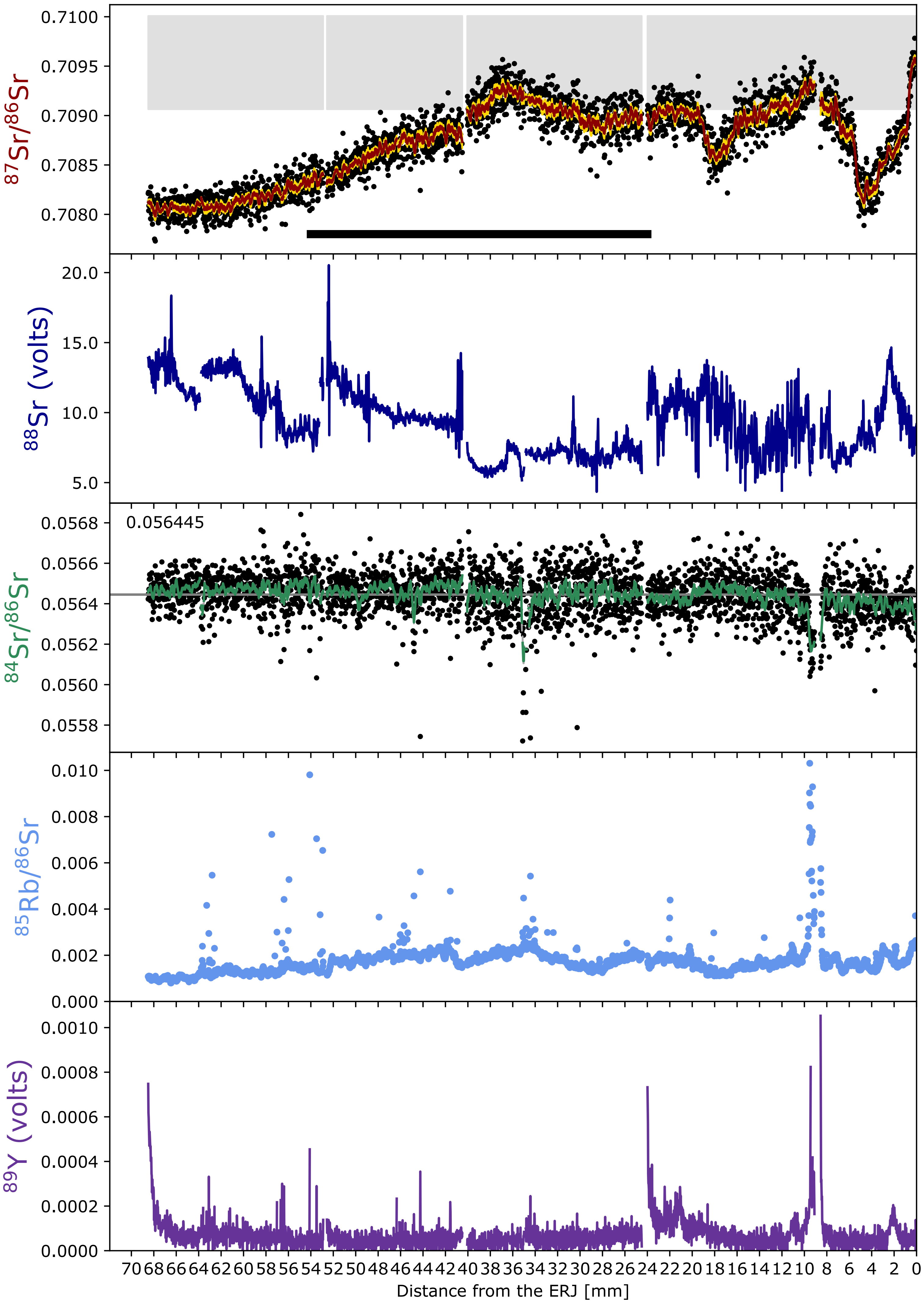

0.7100
0.7095
0.7090
0.7085
0.7080
87Sr/86Sr
20.0
15.0
10.0
5.0
88Sr (volts)
0.056445
0.0568
0.0566
0.0564
0.0562
0.0560
0.0558
84Sr/86Sr
0.010
0.008
0.006
0.004
0.002
0.000
85Rb/86Sr
0.0010
0.0008
0.0006
0.0004
0.0002
0.0000
89Y (volts)
70 68 66 64 62 60 58 56 54 52 50 48 46 44 42 40 38 36 34 32 30 28 26 24 22 20 18 16 14 12 10 8 6 4 2 0
Distance from the ERJ [mm]

I15. ELV21 (M2)

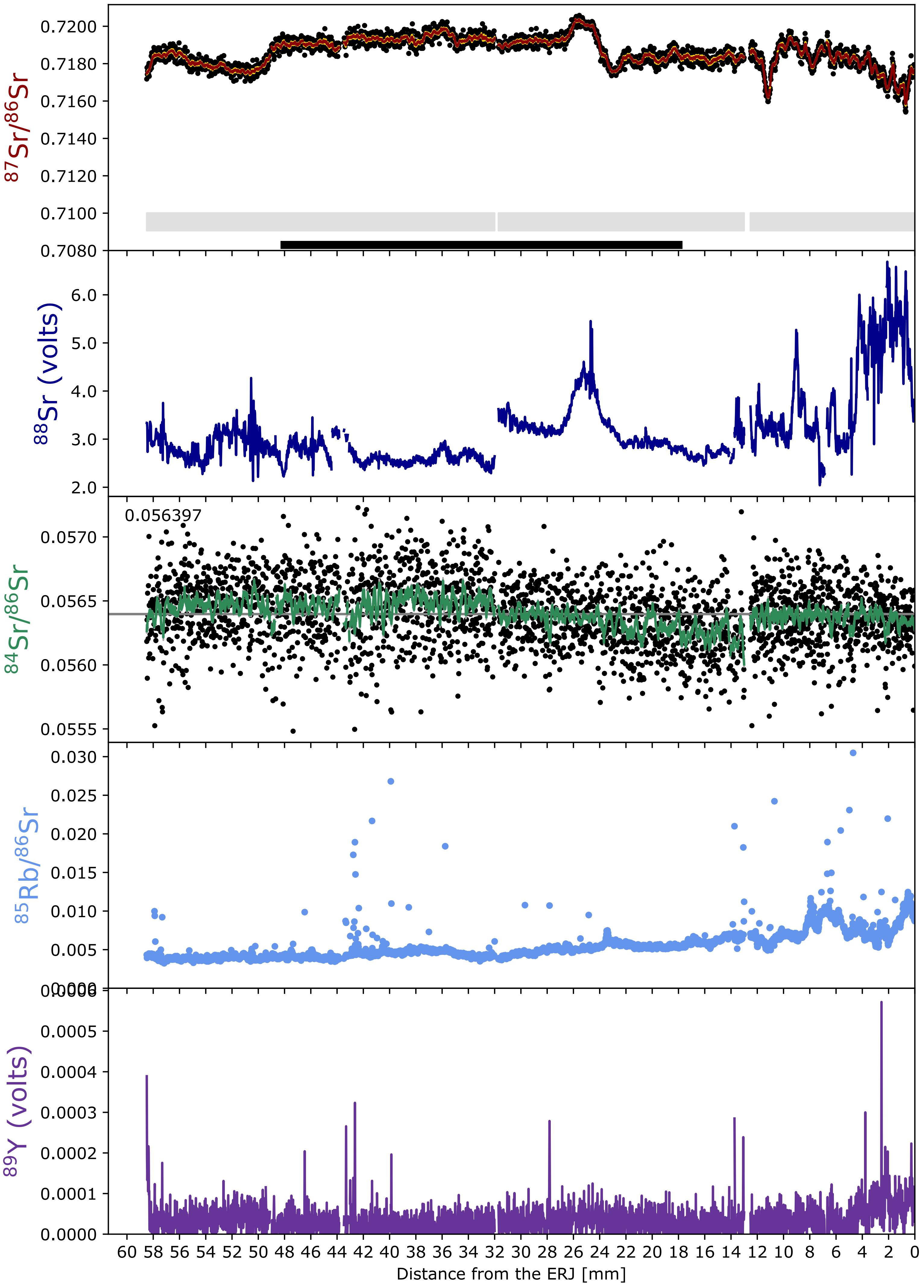

$^{87}Sr/^{86}Sr$
0.7200
0.7180
0.7160
0.7140
0.7120
0.7100
0.7080
^{88}Sr (volts)
6.0
5.0
4.0
3.0
2.0
$^{84}Sr/^{86}Sr$
0.056397
0.0570
0.0565
0.0560
0.0555
$^{85}Rb/^{86}Sr$
0.030
0.025
0.020
0.015
0.010
0.005
0.000
^{89}Y (volts)
0.0006
0.0005
0.0004
0.0003
0.0002
0.0001
0.0000
60 58 56 54 52 50 48 46 44 42 40 38 36 34 32 30 28 26 24 22 20 18 16 14 12 10 8 6 4 2 0
Distance from the ERJ [mm]

I16. ELV22 (M3)

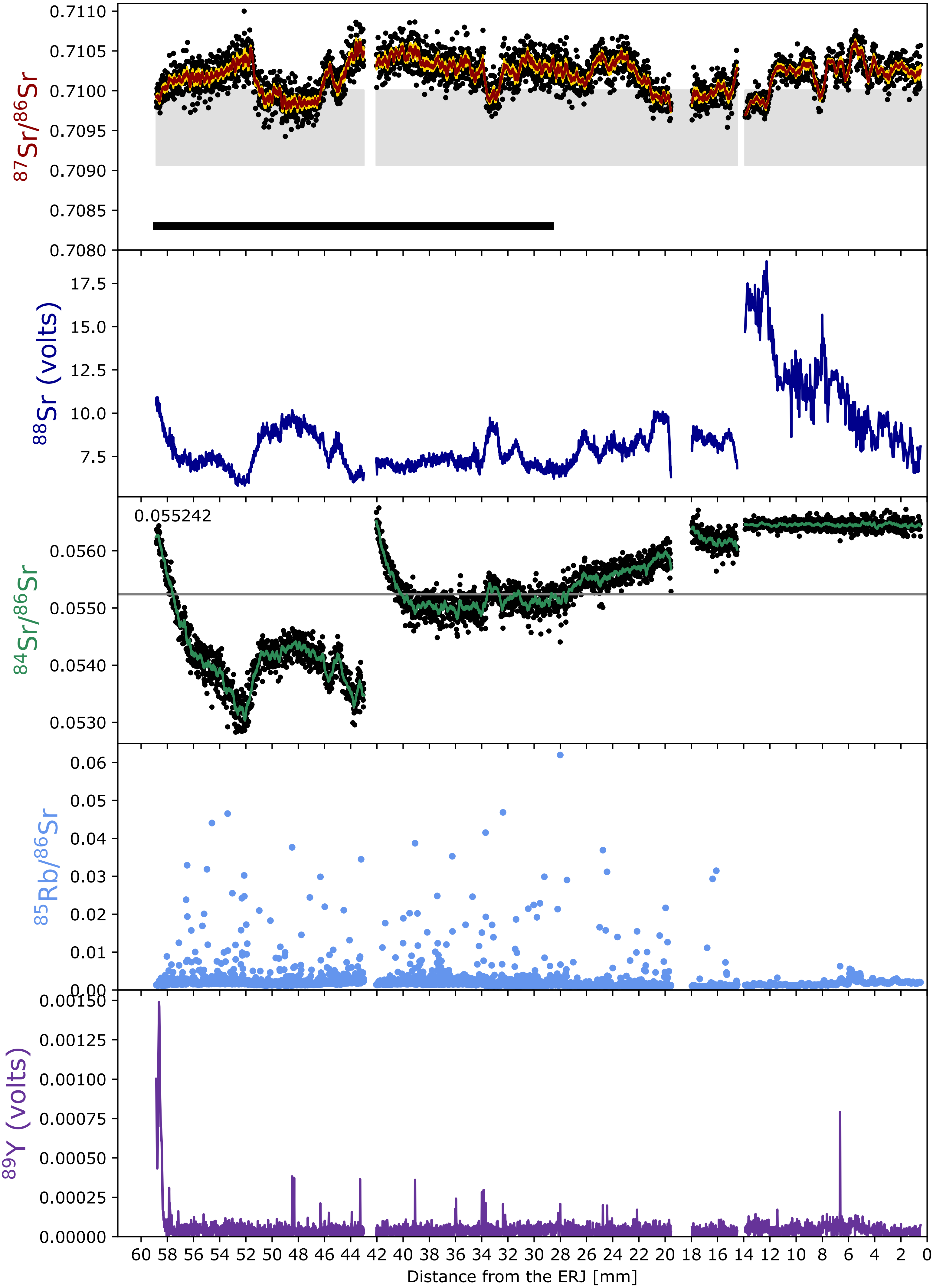

$^{87}Sr/^{86}Sr$
^{88}Sr (volts)
0.055242
$^{84}Sr/^{86}Sr$
$^{85}Rb/^{86}Sr$
^{89}Y (volts)
Distance from the ERJ [mm]

Further supplemental auxiliary files

Data S1 – Tables and isotope data

REFERENCES AND NOTES

1. R. H. C. Davis, The Medieval Warhorse. (Thames and Hudson, 1989).

2. P. Edwards, The Horse Trade of Tudor and Stuart England. (Cambridge Univ. Press, 2009).

3. A. Hyland, The Medieval Warhorse: From Byzantium to the Crusades. (Grange Books, 1994).

4. C. Ameen, H. Benkert, T. Fraser, R. Gordon, M. Holmes, W. Johnson, M. Lauritsen, M. Maltby, K. Rapp, T. Townend, G. P. Baker, L. M. Jones, C. Vo van Qui, R. Webley, R. Liddiard, N. Sykes, O. H. Creighton, R. Thomas, A. K. Outram, In search of the ‘great horse’: A zooarchaeological assessment of horses from England (AD 300–1650). Int. J. Osteoarchaeol. 31, 1247–1257 (2021).

5. J. W. Hawkes, M. J. Heaton, “A closed shaft garderobe and associated medieval structures at Jennings Yard, Windsor, Berkshire” (Wessex Archaeology Reports, no. 3, Trust for Wessex Archaeology, Salisbury, 1993).

6. R. Cowie, A. Pipe, J. Clark, J. Pearce, A late medieval and tudor horse burial ground: Excavations at Elverton Street, Westminster, Archaeol. J. 155, 226–251 (1998).

7. P. Miller, 1 Elverton Street, London SW1, City of Westminster: An archaeological excavation. (Museum of London Archaeology Service, 1994).

8. J. Clark, The Medieval Horse and Its Equipment, c.1150-c.1450. (The Boydell Press, 1995).

9. C. Bronk Ramsey, Bayesian analysis of radiocarbon dates. Radiocarbon 51, 337–360 (2009).

10. P. J. Reimer, W. E. N. Austin, E. Bard, A. Bayliss, P. G. Blackwell, C. Bronk Ramsey, M. Butzin, H. Cheng, R. L. Edwards, M. Friedrich, P. M. Grootes, T. P. Guilderson, I. Hajdas, T. J. Heaton, A. G. Hogg, K. A. Hughen, B. Kromer, S. W. Manning, R. Muscheler, J. G. Palmer, C. Pearson, J. van der Plicht, R. W. Reimer, D. A. Richards, E. M. Scott, J. R. Southon, C. S. M. Turney, L. Wacker, F. Adolphi, U. Büntgen, M. Capano, S. M. Fahrni, A. Fogtmann-Schulz, R. Friedrich, P. Köhler, S. Kudsk, F. Miyake, J. Olsen, F. Reinig, M. Sakamoto, A. Sookdeo, S. Talamo, The IntCal20 northern hemisphere radiocarbon age calibration curve (0–55 cal kBP). Radiocarbon 62, 725–757 (2020).

11. BGS Online Portal, https://geologyviewer.bgs.ac.uk [accessed January 2023].

12. J. A. Evans, J. Montgomery, G. Wildman, N. Boulton, Spatial variations in biosphere 87Sr/86Sr in Britain. J. Geol. Soc. London 167, 1–4 (2010.

13. E. J. Kendall, J. Montgomery, J. A. Evans, C. Stantis, V. Mueller, Mobility, mortality, and the middle ages: Identification of migrant individuals in a 14th century black death cemetery population. Am. J. Phys. Anthropol. 150, 210–222 (2013).

14. H. Shaw, J. Montgomery, R. Redfern, R. Gowland, J. Evans, Identifying migrants in Roman London using lead and strontium stable isotopes. J. Archaeol. Sci. 66, 57–68 (2016).
15. J. A. Evans, C. A. Chenery, K. Mee, C. E. Cartwright, K. A. Lee, A. P. Marchant, L. Hannaford, Biosphere Isotope Domains GB (V1): Interactive Website (British Geological Survey, Interactive Resource, 2018); https://doi.org/10.5285/3b141dce-76fc-4c54-96fa-c232e98010ea.
16. C. P. Bataille, B. E. Crowley, M. J. Wooller, G. J. Bowen, Advances in global bioavailable strontium isoscapes. Palaeogeogr. 555, 109849 (2020).
17. R. Bendrey, D. Vella, A. Zazzo, M. Balasse, S. Lepetz, Exponentially decreasing tooth growth rate in horse teeth: Implications for isotopic analyses. Archaeometry 57, 1104–1124 (2015).
18. K. Hoppe, S. Stover, J. Pascoe, R. Amundson, Tooth enamel biomineralization in extant horses: Implications for isotopic microsampling. Palaeogeogr 206, 355–365 (2004).
19. L. J. Johnson, "Finding radiogenic Sr-isotope biospheres: Can a home in Britain be found for people with high 87Sr/86Sr?" thesis, Durham University (2018).
20. T. Löffelmann, C. Snoeck, J. D. Richards, L. J. Johnson, P. Claeys, J. Montgomery, Sr analyses from only known Scandinavian cremation cemetery in Britain illuminate early Viking journey with horse and dog across the North Sea. PLOS ONE 18, e0280589 (2023).
21. C. Chenery, G. Müldner, J. Evans, H. Eckardt, M. Lewis, Strontium and stable isotope evidence for diet and mobility in Roman Gloucester, UK, J. Archaeol. Sci. 37, 150–163 (2010).
22. R. Madgwick, A. Lamb, H. Sloane, A. Nederbragt, U. Albarella, M. Parker Pearson, J. Evans, A veritable confusion: Use and abuse of isotope analysis in archaeology. Archaeol. J. 178, 361–385 (2021).
23. R. Madgwick, J. Lewis, V. Grimes, P. Guest, On the hoof: Exploring the supply of animals to the Roman legionary fortress at Caerleon using strontium (87Sr/86Sr) isotope analysis. Archaeol. Anthropol. Sci. 11, 223–235 (2019).
24. G. Müldner, D. Frémondeau, J. Evans, A. Jordan, S. Rippon, Putting South-West England on the (strontium isotope) map: A possible origin for highly radiogenic 87Sr/86Sr values from southern Britain. J. Archaeol. Sci. 144, 105628 (2022).
25. L. Johnson, J. Evans, J. Montgomery, C. Chenery, The forest effect: Biosphere 87Sr/86Sr shifts due to changing land use and the implications for migration studies. Sci. Total Environ. 839, 156083 (2022).

26. J. A. Evans, C. A. Chenery, J. Montgomery, A summary of strontium and oxygen isotope variation in archaeological human tooth enamel excavated from Britain. J. Anal. At. Spectrom 27, 754–764 (2012).
27. S. Leggett, A. Rose, E. Praet, P. Le Roux, Multi-tissue and multi-isotope (δ13C, δ15N, δ18O and87/86Sr) data for early medieval human and animal palaeoecology. Ecology 102, e03349 (2021).
28. R. Madgwick, A. L. Lamb, H. Sloane, A. J. Nederbragt, U. Albarella, M. P. Pearson, J. A. Evans, Multi-isotope analysis reveals that feasts in the Stonehenge environs and across Wessex drew people and animals from throughout Britain. Sci. Adv. 5, eaau6078 (2019).
29. A. Delgado Huertas, P. Iacumin, B. Stenni, B. Sánchez Chillón, A. Longinelli, Oxygen isotope variations of phosphate in mammalian bone and tooth enamel. Geochim. Cosmochim. Acta 59, 4299–4305 (1995).
30. S. Pederzani, K. Britton, V. Aldeias, N. Bourgon, H. Fewlass, T. Lauer, S. P. McPherron, Z. Rezek, N. Sirakov, G. M. Smith, R. Spasov, N. H. Tran, T. Tsanova, J. J. Hublin, Subarctic climate for the earliest Homo sapiens in Europe. Sci. Adv. 7, eabi4642 (2021).
31. G. J. Bowen. Gridded maps of the isotopic composition of meteoric waters; www.waterisotopes.org (2023).
32. International Atomic Energy AgencyWorld Meteorological Organization, Global Network of Isotopes in Precipitation. The GNIP Database (International Atomic Energy AgencyWorld Meteorological Organizatio, 2023); https://nucleus.iaea.org/wiser.
33. G. J. Bowen, J. Revenaugh, Interpolating the isotopic composition of modern meteoric precipitation. Water Resour. Res. 39, 1299 (2003).
34. International Atomic Energy AgencyWorld Meteorological Organization, Global Network of Isotopes in Precipitation. The GNIP Database (International Atomic Energy AgencyWorld Meteorological Organization, 2015); https://nucleus.iaea.org/wiser.
35. W. G. Darling, A. H. Bath, J. C. Talbot, The O and H stable isotope composition of freshwaters in the British Isles. 2. Surface waters and groundwater. HESS 2003, 183–195 (2003).
36. J. A. Matthews, K. R. Briffa, The ‘little ice age’: Re-evaluation of an evolving concept. Geogr. Ann. Ser. B 87, 17–36 (2005).
37. T. W. D. Edwards, D. Hammarlund, B. W. Newton, J. Sjolte, H. Linderson, C. Sturm, N. A. St. Amour, J. N. L. Bailey, A. L. Nilsson, Seasonal variability in northern hemisphere atmospheric

circulation during the medieval climate anomaly and the little ice age. Quaternary Sci Rev 165, 102–110 (2017).
38. S. Pederzani, K. Britton, Oxygen isotopes in bioarchaeology: Principles and applications, challenges and opportunities. Earth-Sci. Rev. 188, 77–107 (2019).
39. S. L. Ralston. (MSD Veterinary Manual, 2022), vol. 2023.
40. J. Evans, N. Stoodley, C. Chenery, A strontium and oxygen isotope assessment of a possible fourth century immigrant population in a Hampshire cemetery, southern England. J. Archaeol. Sci. 33, 265–272 (2006).
41. M. Pellegrini, J. Pouncett, M. Jay, M. P. Pearson, M. P. Richards, Tooth enamel oxygen “isoscapes” show a high degree of human mobility in prehistoric Britain. Sci. Rep. 6, 34986 (2016).
42. J. Moore A. Rose, S. Anderson, J. Evans, G. Nowell, D. R. Gröcke, V. Pashley, M. Kirby, J. Montgomery, A multi-isotope (C, N, O, Sr, Pb) study of Iron Age and Roman period skeletons from east Edinburgh, Scotland exploring the relationship between decapitation burials and geographical origins. J. Archaeol. Sci. Rep. 29, 102075 (2020).
43. J. Scorrer K. E. Faillace, A. Hildred, A. J. Nederbragt, M. B. Andersen, M.-A. Millet, A. L. Lamb, R. Madgwick, Diversity aboard a Tudor warship: Investigating the origins of the Mary Rose crew using multi-isotope analysis. R. Soc. Open Sci. 8, 202106 (2021).
44. R. C. Redfern, D. R. Gröcke, A. R. Millard, V. Ridgeway, L. Johnson, J. T. Hefner, Going south of the river: A multidisciplinary analysis of ancestry, mobility and diet in a population from Roman Southwark, London, J. Archaeol. Sci. 74, 11–22 (2016).
45. E. Lightfoot, T. C. O’Connell, On the use of biomineral oxygen isotope data to identify human migrants in the archaeological record: Intra-sample variation, statistical methods and geographical considerations. PLOS One 11, e0153850 (2016).
46. A. J. E. Pryor, R. E. Stevens, T. C. O'Connell, J. R. Lister, Quantification and propagation of errors when converting vertebrate biomineral oxygen isotope data to temperature for palaeoclimate reconstruction. Palaeogeog. Palaeoclimatol. Palaeoecol. 412, 99–107 (2014).
47. M. Willmes, C. P. Bataille, H. F. James, I. Moffat, L. McMorrow, L. Kinsley, R. A. Armstrong, S. Eggins, R. Grün, Mapping of bioavailable strontium isotope ratios in France for archaeological provenance studies. Appl. Geochem. 90, 75–86 (2018).
48. F. Lugli, A. Cipriani, L. Bruno, F. Ronchetti, C. Cavazzuti, S. Benazzi, A strontium isoscape of Italy for provenance studies. Chem. Geol. 587, 120624 (2022).

49. R. Frei, K. M. Frei, The geographic distribution of Sr isotopes from surface waters and soil extracts over the island of Bornholm (Denmark) – A base for provenance studies in archaeology and agriculture. Appl. Geochem. 38, 147–160 (2013).
50. G. Piličiauskienė L. Kurila, E. Simčenka, M. Kielman-Schmitt, K. Lidén, E. Kooijman, G. Piličiauskas, The origin of Late Roman period–post-migration period Lithuanian horses. Heritage. 5, 332–352 (2022).
51. S. Viranta, K. Mannermaa, Mesowear analysis on Finnish medieval horses. Ann. Zool. 51, 119–122 (2014).
52. J. Hoogewerff, W. Papesch, M. Kralik, M. Berner, P. Vroon, H. Miesbauer, O. Gaber, K. H. Künzel, J. Kleinjans, The last domicile of the iceman from hauslabjoch: A geochemical approach using sr, c and o isotopes and trace element signatures. J. Archaeol. Sci. 28, 983–989 (2001).
53. F. Morenzoni, L'achat et la vente de chevaux d'apres les registres des notaires fribourgeois de la fin du Moyen Age. Rivista Storica Svizzera 48, 131–148 (1998).
54. G. Chiesi, Venire cum equis ad partes Lumbardie. Mercanti confederati alle fiere prealpine nella seconda metà del XV secolo. Rivista Storica Svizzera 44, 252–265 (1994).
55. T. Frei, Einsiedeln als Pferdezentrum der Innerschweiz. Schwyzer Hefte 102, 15–26 (2015).
56. J. Jobst, The Liminal Horse, A. Ropa, R. Maguire, Eds. (Trivent Publishing, 2021), pp. 13–49.
57. S. Harrison, How to make a warhorse: Violence and behavioural control in late medieval hippiatric treatises. J. Mediev. Hist. 48, 347–367 (2022).
58. C. Ameen, G. P. Baker, H. Benkert, C. Vo van Qui, R. Webley, R. Liddiard, A. K. Outram, O. H. Creighton, Interdisciplinary approaches to the medieval warhorse. Cheiron: The International Journal of Equine and Equestrian 1, 84–103 (2021).
59. M. C. B. Dawes, Register of Edward the Black Prince. Part 1 (HMSO, 1930), pp. 1346–1348.
60. T. Almeroth-Williams, The story of Smithfield market. Lond. J. 36, 72–76 (2011).
61. British Library, Library additional manuscript 46348. Inventory of the jewels, Plate and other effects of Henry VIII.XX.
62. H. Roberts, W. H. Godfrey, Lambeth bridge and its predecessor the horseferry. Survey of London, 23, Lambeth: South Bank and Vauxhall 118–121 (1951).
63. TNA E372/189; TNA E101/102/39. 1343-44. Account of William de Fremelesworth, Keeper of the Great Horses (at Westminster).

64. TNA E372/192mm 46r-46v. 1346-47. Account of William le Ferrour, Keeper of the Great Horses of the King South of the Trent).
65. A. Young, Tudor and Jacobean Tournaments. (George Philip, 1987).
66. H. M. Colvin, J. Summerson, M. Biddle, H. R. Hale, M. Merriman, The History of the King's Works. Volume IV (Part II) (HMSO, 1982), pp. 1485–1660.
67. E. Levitt, Loyalty to the Monarchy in Late Medieval and Early Modern Britain, c. 1400–1688, M. Ward, M. Hefferan, Eds. (Palgrave Macmillan, 2020), pp. 15–35.
68. A. von den Driesch, A Guide to the Measurement of Animal Bones from Archaeological Sites. (Peabody Museum, 1976).
69. E. May, Widerristhöhe und Langknochenmaße bei Pferden – ein immer noch aktuelles Problem. Zeitschrift für Säugetierkunde 50, 368–382 (1985).
70. L. Forster, Echoing Hooves: Studies on Horses and Their Effects on Medieval Societies, A. Ropa, T. Dawson, Eds. (Brill, 2022), pp. 223–245.
71. J. Clark, in The Horse in Premodern European Culture, A. Ropa, T. Dawson, Eds. (Medieval Institute Publications, 2020), pp. 197–192.
72. S. Lepetz B. Clavel, D. Alioğlu, L. Chauvey, S. Schiavinato, L. Tonasso-Calvière, X. Liu, A. Fages, N. Khan, A. Seguin-Orlando, C. D. Sarkissian, P. Clavel, O. Estrada, C. Gaunitz, J.-M. Aury, M. Barme, N. Boulbes, A. Bourgois, F. Decanter, S. Foucras, S. Frère, A. Gardeisen, G. Jouanin, C. Méla, N. Morand, A. N. Espinet, A. Perdereau, O. Putelat, J. Rivière, O. Robin, M. Salin, S. Valenzuela-Lamas, C. Vallet, J.-H. Yvinec, P. Wincker, L. Orlando, Historical management of equine resources in France from the Iron Age to the Modern Period. J. Archaeol. Sci. Rep. 40, 103250 (2021).
73. M. Schubert, M. Mashkour, C. Gaunitz, A. Fages, A. Seguin-Orlando, S. Sheikhi, A. H. Alfarhan, S. A. Alquraishi, K. A. S. al-Rasheid, R. Chuang, L. Ermini, C. Gamba, J. Weinstock, O. Vedat, L. Orlando, Zonkey: A simple, accurate and sensitive pipeline to genetically identify equine F1-hybrids in archaeological assemblages. J. Archaeol. Sci. 78, 147–157 (2017).
74. A. J. E. Pryor, T. Insoll, L. Evis, Laser ablation strontium isotope analysis of human remains from Harlaa and Sofi, eastern Ethiopia, and the implications for Islamisation and mobility. STAR: Science & Technology of Archaeological Research 6, 113–136 (2020).
75. J. Lewis, C. D. Coath, A. W. G. Pike, An improved protocol for 87Sr/86Sr by laser ablation multi-collector inductively coupled plasma mass spectrometry using oxide reduction and a customised plasma interface. Chem. Geol. 390, 173–181 (2014).

76. W. A. Russell, D. A. Papanastassiou, T. A. Tombrello, Ca isotope fractionation on the Earth and other solar system materials. Geochim. Cosmochim. Acta 42, 1075–1090 (1978).
77. J. Woodhead, S. Swearer, J. Hergt, R. Maas, In situ Sr-isotope analysis of carbonates by LA-MC-ICP-MS: Interference corrections, high spatial resolution and an example from otolith studies. J. Anal. At. Spectrom 20, 22–27 (2005).
78. J. R. de Laeter J. K. Böhlke, P. De Bièvre, H. Hidaka, H. S. Peiser, K. J. R. Rosman, P. D. P. Taylor, Atomic weights of the elements. Review 2000 (IUPAC Technical Report). Pure Appl. Chem. 75, 683–800 (2003).
79. M. Pellegrini, C. Snoeck, Comparing bioapatite carbonate pre-treatments for isotopic measurements: Part 2 — Impact on carbon and oxygen isotope compositions. Chem. Geol. 420, 88–96 (2016).
80. M. Levine, Ageing and Sexing Animal Bones from Archaeological Sites, B. Wilson, C. Grigson, S. Payne, Eds. (BAR Publishing, 1982), pp. 223–225.
81. M. Levine, K. E. Whitwell, L. B. Jeffcot, Abnormal thoracic vertebrae and the evolution of horse husbandry. Archaeofauna 14, 93–109 (2005).
82. D. J. Rackham, The Medieval Horse and Its Equipment, J. Clark, Ed. (The Boydell Press with the Museum of London, 2004), pp. 169–174.
83. R. Bendrey, New methods for the identification of evidence for bitting on horse remains from archaeological sites. J. Archaeol. Sci. 34, 1036–1050 (2007).
84. M. F. Thirlwall, Long-term reproducibility of multicollector Sr and Nd isotope ratio analysis. Chem. Geol.: Isot. Geosci. Sect. 94, 85–104 (1991).
85. S.-T. Kim, T. B. Coplen, J. Horita, Normalization of stable isotope data for carbonate minerals: Implementation of IUPAC guidelines. Geochim. Cosmochim. Acta 158, 276–289 (2015).
86. P. Iacumin, H. Bocherens, A. Mariotti, A. Longinelli, Oxygen isotope analyses of co-existing carbonate and phosphate in biogenic apatite: A way to monitor diagenetic alteration of bone phosphate? Earth Planet Sc. Lett. 142, 1–6 (1996).
87. S. A. Blumenthal, T. E. Cerling, T. M. Smiley, C. E. Badgley, T. W. Plummer, Isotopic records of climate seasonality in equid teeth. Geochim. Cosmochim. Acta 260, 329–348 (2019).
88. K. A. Hoppe, R. Amundson, M. Vavra, M. P. McClaran, D. L. Anderson, Isotopic analysis of tooth enamel carbonate from modern North American feral Horses: Implications for paleoenvironmental reconstructions. Palaeogeogr. Palaeoclimatol. Palaeoecol. 203, 299–311 (2004).

89. N. Miraglia, M. Saastamoinen, W. Martin-Rosset, Nutrition and Feeding of the Broodmare. EAAP Scientific Series 120, N. Miraglia, W. Martin-Rosset, Eds. (Wageningen Academic Publishers, 2006), pp. 279–297.
